# Sustainable Synthesis of Silicon Precursors Coupled with Hydrogen Delivery Based on Circular Economy via Molecular Cobalt-Based Catalysts

**DOI:** 10.1021/acssuschemeng.2c04444

**Published:** 2022-12-08

**Authors:** Silvia Gutiérrez-Tarriño, Sergio Rojas-Buzo, Manuel A. Ortuño, Pascual Oña-Burgos

**Affiliations:** † Instituto de Tecnología Química, Universitat Politècnica de València-Consejo Superior de Investigaciones Científicas (UPV-CSIC), Avda. de los Naranjos s/n, Valencia 46022, Spain; ‡ Department of Chemistry, NIS and INSTM Reference Centre, Università di Torino, Torino 10125, Italy; § Centro Singular de Investigación en Química Biolóxica e Materiais Moleculares (CIQUS), Universidade de Santiago de Compostela, Santiago de Compostela 15782, Spain; ∥ Department of Chemistry and Physics, Research Centre CIAIMBITAL, University of Almería, Ctra. Sacramento, s/n, Almería 04120, Spain

**Keywords:** hydrogen delivery, green hydrogen, alkene hydrosilylation, dehydrogenative coupling, cobalt complex, homogeneous catalysis

## Abstract

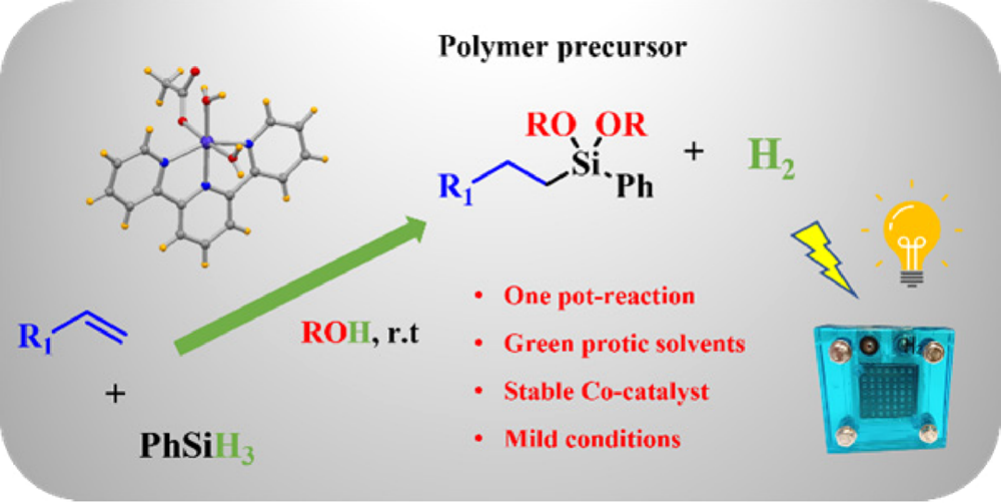

The development of a circular economy is a key target to reduce our dependence on fossil fuels and create more sustainable processes. Concerning hydrogen as an energy vector, the use of liquid organic hydrogen carriers is a promising strategy, but most of them present limitations for hydrogen release, such as harsh reaction conditions, poor recyclability, and low-value byproducts. Herein, we present a novel sustainable methodology to produce value-added silicon precursors and concomitant hydrogen via dehydrogenative coupling by using an air- and water-stable cobalt-based catalyst synthesized from cheap and commercially available starting materials. This methodology is applied to the one-pot synthesis of a wide range of alkoxy-substituted silanes using different hydrosilanes and terminal alkenes as reactants in alcohols as green solvents under mild reaction conditions (room temperature and 0.1 mol % cobalt loading). We also demonstrate that the selectivity toward hydrosilylation/hydroalkoxysilylation can be fully controlled by varying the alcohol/water ratio. This implies the development of a circular approach for hydrosilylation/hydroalkoxysilylation reactions, which is unprecedented in this research field up to date. Kinetic and in situ spectroscopic studies (electron paramagnetic resonance, nuclear magnetic resonance, and electrospray ionization mass spectrometry), together with density functional theory simulations, further provide a detailed mechanistic picture of the dehydrogenative coupling and subsequent hydrosilylation. Finally, we illustrate the application of our catalytic system in the synthesis of an industrially relevant polymer precursor coupled with the production of green hydrogen on demand.

## Abstract 2

Sustainable methodology for the one-pot synthesis of alkoxysilanes coupled with hydrogen delivery using a cobalt-based catalyst under mild reaction conditions.

## Introduction

The combination of hydrogen and oxygen to produce green energy and water as the only byproduct is one of the most promising alternatives to actual carbon-based fuels.^1^ However, hydrogen storage is still the principal limitation of using hydrogen to obtain clean energy.^2^ In this sense, the use of liquid organic hydrogen carriers (LOHCs) is a promising strategy, since the energy storage density and tractability are similar to petroleum-derived fuels.^3−5^ Actually, several types of LOHCs, such as cycloalkanes,^6^ N-heterocycles,^7,8^ formic acid,^9−12^ or ammonia borane,^13−15^ are already employed for that purpose. Nevertheless, these LOHCs still present some drawbacks such as the high temperature needed for hydrogen release,^16^ production of CO_2_,^17−19^ or difficult regeneration processes.^9,20,21^ All these approaches share a common weakness: low sustainability according to a circular economy perspective.

**Scheme 1:**
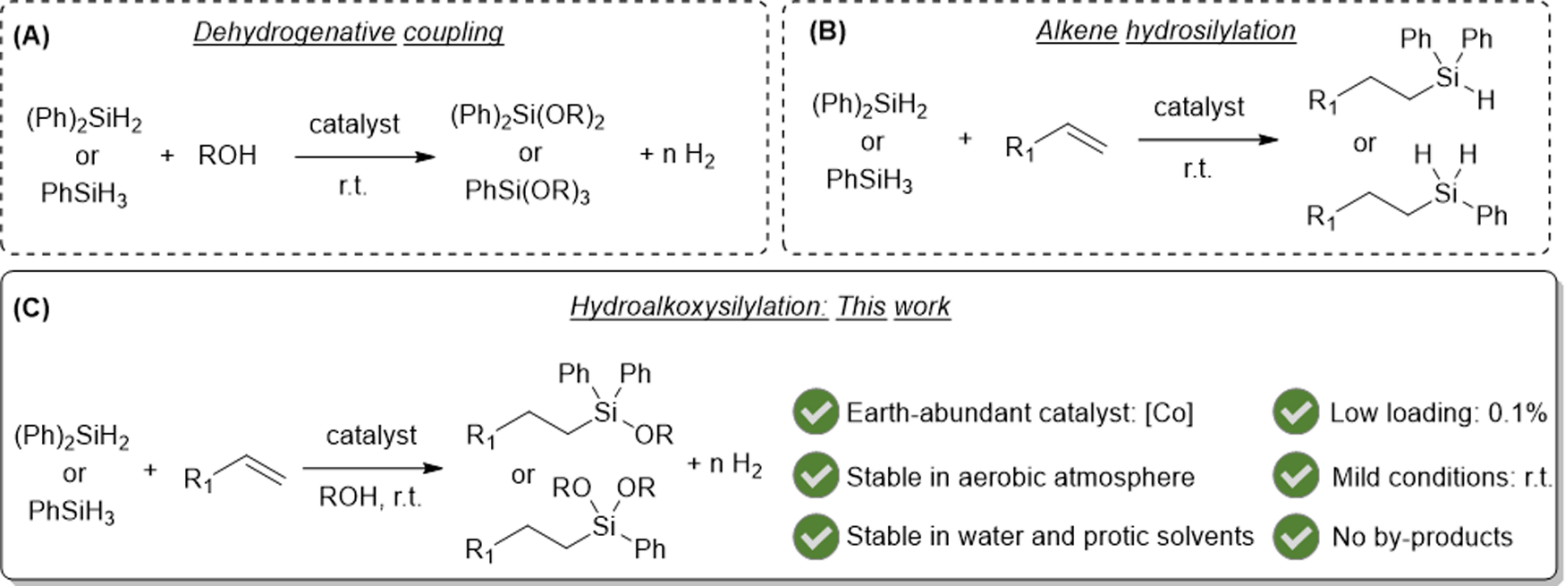
Strategies for the Synthesis of Alkoxysilanes: (A) Dehydrogenative Coupling, (B) Alkene Hydrosilylation, and (C) Hydroalkoxysilylation

As an alternative approach to address these current limitations, we turn to the dehydrogenative coupling of hydrosilanes with alcohols, which produces silyl ethers and hydrogen at low reaction temperatures, even at 0 °C^22−24^ (Scheme 1A). Such silyl ethers are value-added chemical intermediates in the synthesis of silicones and hybrid organic–inorganic materials. For example, the resulting product of the hydrosilylation reaction of *n*-octene with triethoxysilane, (*n*-octyl)Si(OEt)_3_, is produced in large scale (>6000 tons per year) for the construction industry.^25^ In this sense, alkene hydrosilylation is an atom-economic reaction employed to synthesize monomers for the synthesis of silicon-based materials including biomedical sensors, pressure-sensitive adhesives, and chromatography stationary phases and molds.^26−29^ Although the most common industrial method for the production of these organosilanes uses Pt-catalysts based on Speier^30^ and Karstedt^31^ complexes, different strategies based on Earth-abundant and environmentally benign transition metals (Fe, Co, or Ni) have been developed to enable the state-of-the-art of alkene hydrosilylation reactions (Scheme 1B).^32^ Unfortunately, the extreme air and moisture sensitivities of the catalyst precursors prevent their widespread implementation.^33−35^ In that line, we have recently demonstrated the efficiency of an aerobic-stable cobalt catalyst consisting in commercial available precursors for the selective alkene anti-Markovnikov hydrosilylation reaction avoiding the use of external activators.^36^ In addition, sustainable synthesis of silicon precursors via hydrosilylation employing green protic solvents has not been achieved up to date.

In the alkene hydrosilylation process, both alkoxysilanes and hydrosilanes could be used as starting materials. However, alkoxysilanes present the drawback of being more expensive than hydrosilanes and can polymerize easily, so inert and low temperature conditions are demanded in their storage to prevent side reactions. For these reasons, the in situ production of these alkoxysilanes from hydrosilanes coupled with the hydrosilylation process could be tentatively employed to produce value-added polymer intermediates (Scheme 1C). Nevertheless, only one example for the selective anti-Markovnikov hydroalkoxysilylation reaction has been reported. In that study, homogeneous Pd catalysts and dichloromethane solvent are required, and subsequently, corrosive hydrogen halides are produced as byproducts.^37^

Having this target in mind, we have designed a new methodology (Scheme 1C) which consists in the one-pot synthesis of alkoxy-substituted silanes coupled with hydrogen delivery using stable hydrosilanes and terminal alkenes, green protic solvents, and the cobalt-based catalyst [Co(OAc)(H_2_O)_2_(tpy)]OAc (**cat-1**).^36^ The presence of water molecules in the coordination sphere makes this cobalt complex stable in aqueous media and soluble in protic solvents. This provides an immediate advantage over other cobalt precatalysts reported by Thomas,^34^ Chirik,^25,38,39^ or Nagashima,^40^ in which the presence of alkyl ligands makes them air and moisture sensitive, and therefore, they must be handled in an inert atmosphere using anhydrous reagents and solvents. In situ spectroscopic experiments (electron paramagnetic resonance (EPR), nuclear magnetic resonance (NMR), and Raman) together with computational calculations were employed to better understand the general mechanism for the hydroalkoxysilylation reaction. These results reveal that this one-pot reaction proceeds first with the formation of alkoxysilanes via dehydrogenative coupling.^24,41,42^ Then, the generated alkoxysilane reacts with the alkene via hydrosilylation. The reaction proceeds with high selectivity to the anti-Markovnikov product under very mild conditions (0 °C to room temperature and 0.1 mol % metal loading). Moreover, we can control the selectivity toward hydrosilylation or alkoxysilylation processes by varying the solvent mixture. Finally, the Co-based system presented in this work could be applied to the synthesis of an industrially relevant polymer precursor and to produce clean energy.

## Results and Discussion

### Synthesis of the Catalyst

Cobalt(II) acetate tetrahydrate and 2,2′:6′,2″-terpyridine (tpy) were employed as the cobalt source and organic ligand, respectively, to generate the known complex [Co(OAc)(H_2_O)_2_(tpy)]OAc (**cat-1**) (for more details, see the Supporting Information and Figures S1 and S2).

### Catalytic Alkoxylation Reaction of Phenylsilane with Methanol

We initially tested the catalytic dehydrogenative coupling reaction of phenylsilane and methanol (Table 1). When using the cobalt catalyst **cat-1**, an immediate color change from orange to dark brown and an increment of the pressure were observed when the silane was added to the solution of the catalyst in methanol, which indicates the formation of the active species and the generation of hydrogen. Complete phenylsilane conversion to the trimethoxysilane product **C** was achieved at room temperature after 1 h (entry 1).

**Table 1:**
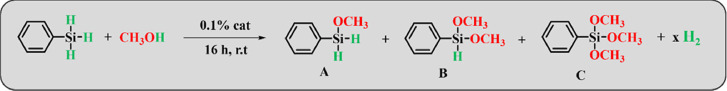
Evaluation of Different Catalysts for the Catalytic Dehydrogenative Coupling of PhSiH_3_ with Methanol^a^

entry	catalyst^b^	conversion (%)^b^	**A** (%)	**B** (%)	**C** (%)
1	[Co(OAc)(H_2_O)_2_(tpy)]OAc **(cat-1)**^c^	>99			>99
2	Co(OAc)_2_·4H_2_O	>99			>99
3	Co(acac)_2_·H_2_O	>99			>99
4	Co(NO_3_)_2_·6H_2_O	33		14.3	18.7
5	Fe(OAc)_2_ + tpy	>99			>99
6	Fe(OAc)_2_	>99			>99
7	Ni(OAc)_2_ + tpy	>99		34	66
8	Ni(OAc)_2_·4H_2_O	>99	5	10	85
9	Tpy	0			
10^d^	Karstedt catalyst^d^	>99			>99

a All reactions were performed with 0.89 mmol of phenylsilane and 0.1% of catalyst in 0.2 mL of methanol under aerobic conditions.

b Conversions were determined by ^1^H-NMR analysis of the crude reaction mixture using 1,4-dinitrobenzene as the standard.

c 1 h of reaction time.

d Pt_2_[(Me_2_SiCH=CH_2_)_2_O]_3_.

Additional homogeneous cobalt catalysts were tested for comparison. Both acetate and acetylacetonate precursors are active in the dehydrogenative coupling reaction (entries 2 and 3). In the case of cobalt nitrate, a mixture of di- and tri-methoxylated products, **B** and **C**, respectively, was obtained (entry 4). Similar iron and nickel complexes and their corresponding precursors were next evaluated. In these cases, iron catalysts yield complete conversion to the trimethoxylated product **C** (entries 5 and 6), while nickel catalysts give a mixture of products **A**, **B**, and **C** (entries 7 and 8). A blank reaction with only the tpy ligand confirmed that the activity is due to the metal center of the catalyst (entry 9). Finally, the Karstedt catalyst, the most common one in the hydrosilylation reaction, also performed well (entry 10). These data indicate that most catalysts present high activity and selectivity for the dehydrogenative process. However, the benefits of **cat-1** with respect to other systems will become clear when coupling this step to the hydrosilylation reaction.

### One-Pot Synthesis of Alkoxysilanes from Phenylsilane and Styrene: Evaluation of the Catalytic Activity

Once the catalytic activity of **cat-1** has been studied for the dehydrogenative coupling of PhSiH_3_ with methanol, and taking into account its catalytic activity in the hydrosilylation of alkenes,^36^ we next pursued the dehydrogenative coupling of silanes with alcohols coupled with hydrosilylation reaction of alkenes to produce alkoxy-substituted silanes and hydrogen in an one-pot process (Table 2). **cat-1** produced the dimethoxy hydrosilylated product **F** with 99% conversion and 95% selectivity (entry 1). Similar to Table 1, other catalysts were also examined. Although cobalt acetate, cobalt acetylacetonate, and cobalt nitrate were active for the catalytic dehydrogenative coupling reaction, they are not for the hydrosilylation process (entries 2, 3, and 4). The same behavior is observed for iron species, which form exclusively product **C** (entries 5 and 6).

**Table 2:**
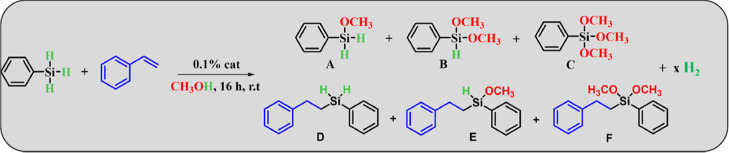
Evaluation of Different Catalysts for the One-Pot Hydrosilylation of Styrene with PhSiH_3_ Coupled with Catalytic Dehydrogenative Coupling of PhSiH_3_ with Methanol^a^

entry	catalyst^b^	conv. (%)^b^	**A** (%)	**B** (%)	**C** (%)	**D** (%)	**E** (%)	**F** (%)
1	[Co(OAc)(H_2_O)_2_(tpy)]OAc (**cat-1**)^c^	>99				5		95
2	Co(OAc)_2_·4H_2_O	>99			>99			
3	Co(acac)_2_·H_2_O	>99			>99			
4	Co(NO_3_)_2_·6H_2_O	33		14.3	18.7			
5	Fe(OAc)_2_ + tpy	>99			>99			
6	Fe(OAc)_2_·9H_2_O	>99			>99			
7	Ni(OAc)_2_ + tpy	>99	4.4	26.3	51.8		17.5	
8	Ni(OAc)_2_·4H_2_O	>99		41.4	36		22.6	
9	tpy	0						
10	Karstedt catalyst^d^	>99			34	66		

a All reactions were performed with 0.89 mmol of phenylsilane, 0.89 mmol of styrene, and 0.1% of catalyst in 0.2 mL of methanol under aerobic conditions.

b Disappearance of phenylsilane determined by ^1^H-NMR analysis of the crude reaction mixture using 1,4-dinitrobenzene as the standard.

c 6 h of reaction time.

d Pt_2_[(Me_2_SiCH=CH_2_)_2_O]_3_.

On the other hand, the nickel salt and the complex with tpy (entries 7 and 8, respectively) present low activity, where an appreciable amount of styrene remains unreacted due to the formation of the products from the dehydrogenative coupling reaction. Another blank reaction again confirmed the catalytic role of the metal in both dehydrogenative and hydrosilylation steps (entry 9). Finally, for the Karstedt catalyst, both reactions compete, and a mixture of products **C** and **D** is collected (entry 10). These results demonstrate the superior performance of **cat-1** with respect to other systems for the one-pot reaction, both in terms of activity and selectivity.

### Substrate Screening for the One-Pot Catalytic Reaction

With these promising results obtained for **cat-1**, we next explored the scope of alkene substrates and solvents (Table 3). When the one-pot reaction was performed with different alkenes and primary hydrosilanes, such as phenylsilane, in methanol or ethanol, the alkoxysilylated products (dimethoxy or diethoxy, respectively) were observed as the main products in all cases. The catalyst is highly active for this one-pot process under mild aerobic conditions (room temperature and 0.1% metal loading), leading to complete conversion with high selectivity in only 6 h using aromatic and aliphatic alkenes and being selective to terminal versus internal alkenes. Interestingly, when performing the reaction in a mixture of one of these alcohols and water in the proportion 1:1, hydrosilylation products were obtained with high selectivity while retaining anti-Markovnikov configuration.

**Table 3:**
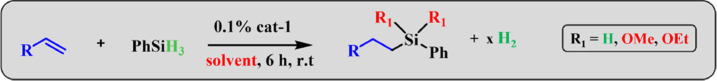

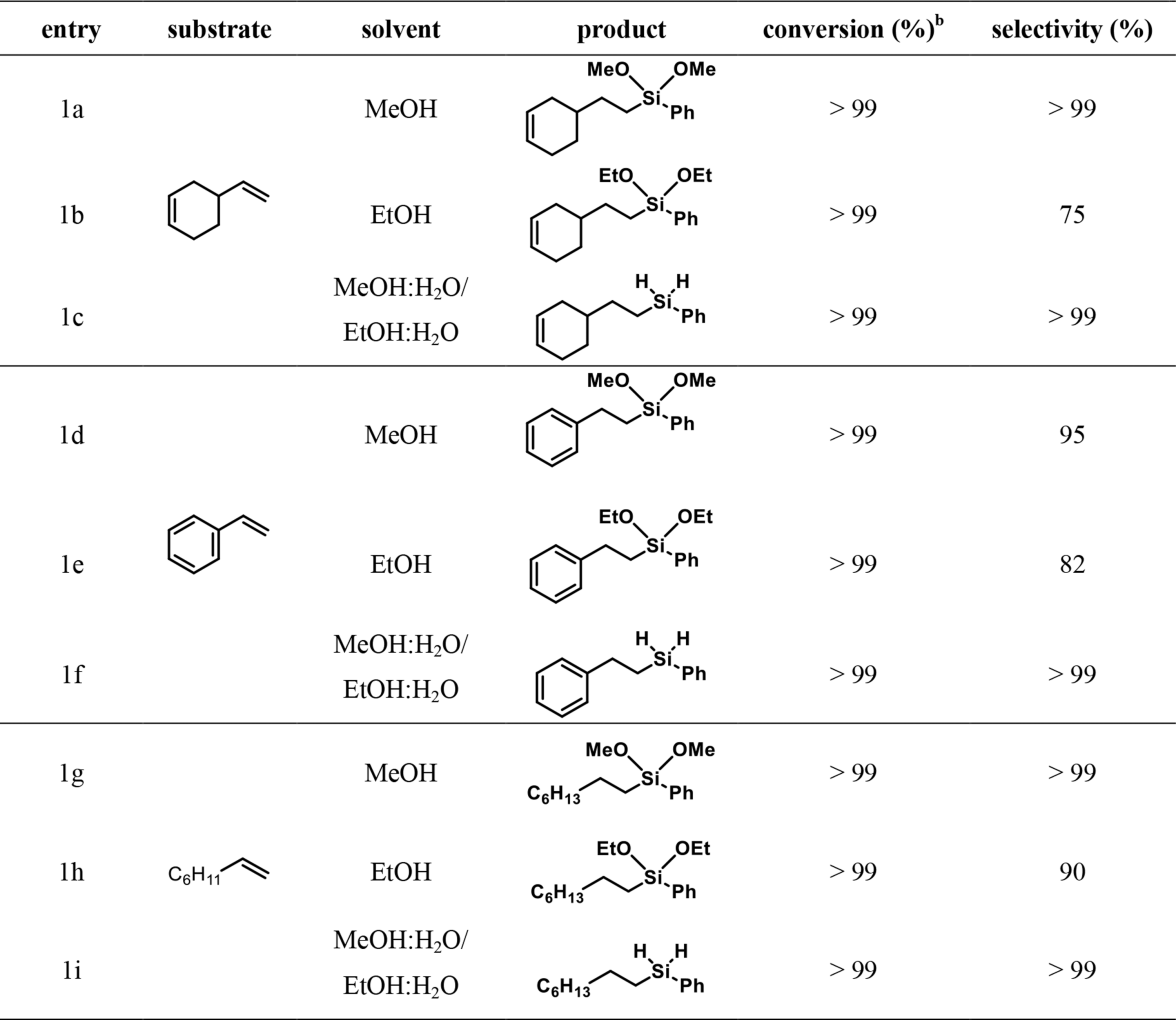
Evaluation of **cat-1** for the Hydrosilylation/Alkoxysilylation of Alkenes with PhSiH_3_^a^

a All reactions were performed on a 0.89 mmol scale using a 1:1 silane/olefin mixture under aerobic conditions.

b Conversion of phenylsilane was determined by ^1^H-NMR analysis of the crude reaction mixture using 1,4-dinitrobenzene as the standard.

The same results were obtained for secondary hydrosilanes such as diphenylsilane: alkoxysilylated products are formed in methanol and ethanol, while hydrosilanes are formed in a mixture of alcohol/water in proportion 1:1 (Table S1). The only difference was that, due to the steric impediments generated by the aromatic rings, the reactivity with diphenylsilane is considerably lower than that with phenylsilane, so the reaction time was incremented to 24 h. Nevertheless, complete conversion and high selectivity for the desired products were obtained under the same mild aerobic conditions.

### Unraveling the Nature of Intermediates and Catalytic Active Sites: Kinetic, In Situ, and Computational Studies

**Figure 1:**
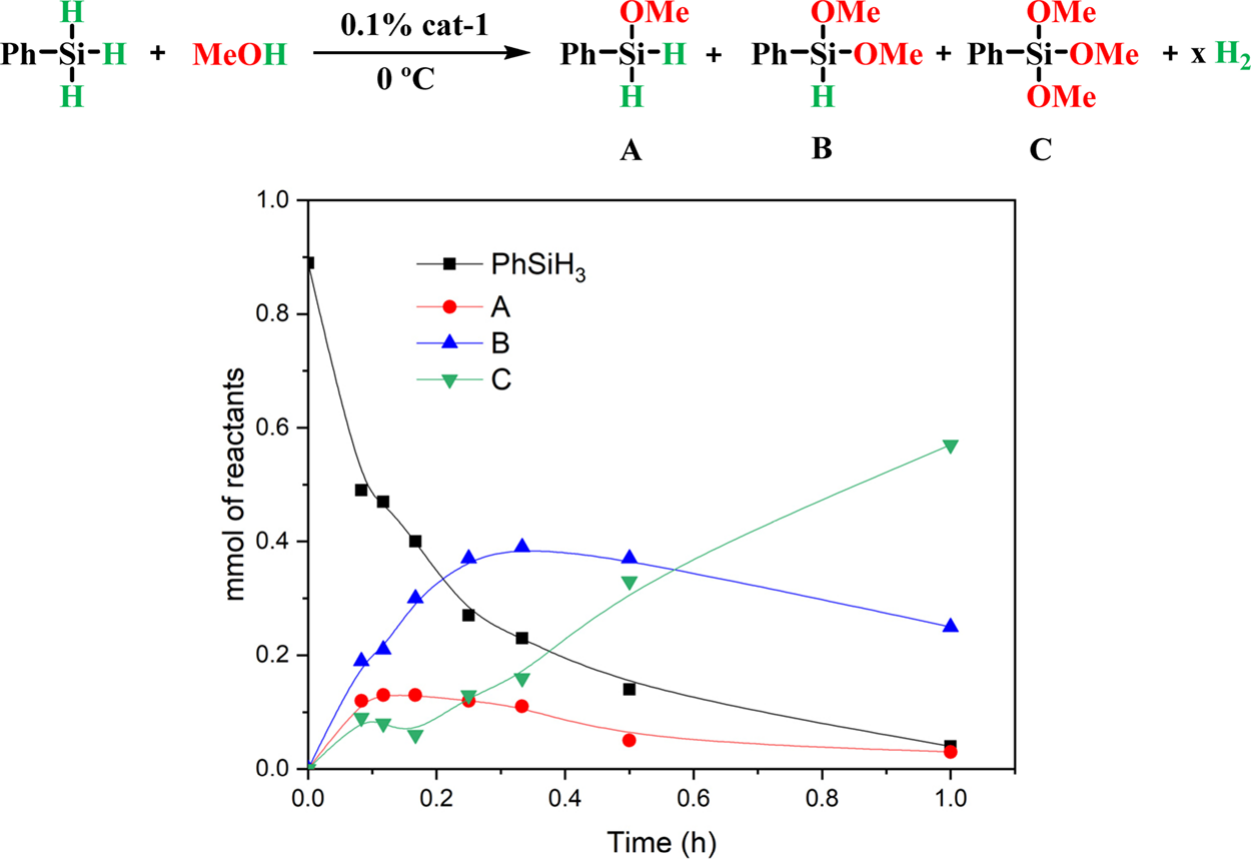
Alkoxysilylation reaction of phenylsilane (black) with methanol at 0 °C catalyzed by **cat-1** to give products **A** (red), **B** (blue), and **C** (green).

In order to elucidate the nature of the intermediates in the one-pot catalytic reaction, different kinetic experiments have been carried out. First, the alkoxysilylation reaction of phenylsilane with methanol has been followed over time using **cat-1** (0.1 mol % Co). Since the phenylsilane conversion is ∼70% after only 5 min at room temperature, the catalytic reaction was monitored at 0 °C to better identify the reaction intermediates. Under these optimized reaction conditions, ∼95% PhSiH_3_ conversion was obtained after only 1 h (Figure 1). Moreover, the yield of the hydromethoxysilane intermediates **A** and **B** increases exponentially until 15 min, time from which both begin to be converted to trimethoxysilane product **C** (Figure 1). These data indicate that the inclusion of one or two MeO groups in the silane increases its reactivity.

In a similar way, the alkoxysilylation reaction of diphenylsilane with methanol has been studied kinetically (Figure S3). However, due to more steric impediments, the amount of dimethoxydiphenylsilane detected is considerably lower even at longer reaction times. Finally, the influence of the isotopic exchange in methanol has been studied by NMR spectroscopy for the diphenylsilane substrate. First, the initial amount of CD_3_OH (6%) in the CD_3_OD solvent was measured using toluene as the internal standard. Subsequently, after the addition of 10 equivalents of diphenylsilane, the calculated ratio of HD/H_2_ generated (Figure S4) was 3/1.3, confirming an isotopic effect *k*_H_/*k*_D_ = 5 in this alkoxylation reaction. This fact corroborates that the released hydrogen gas comes from the methanol and the silane reactant.

**Scheme 2:**
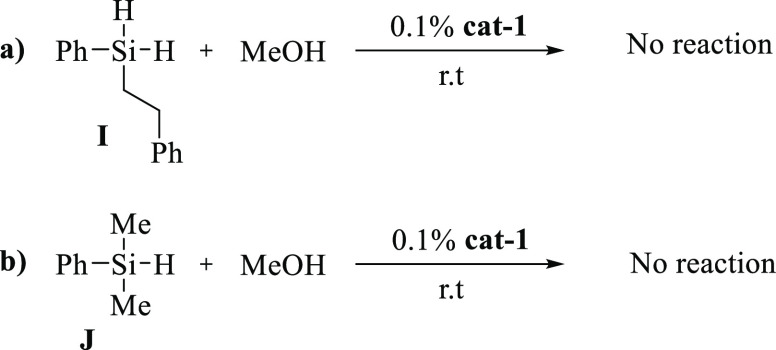
Alkoxysilylation Reaction of Aliphatic-Substituted Phenylsilane: (a) Phenethyl(phenyl)silane and (b) Dimethylphenylsilane with Methanol at 25 °C Catalyzed by **cat-1**

Since the hydroalkoxysilylation reaction of phenylsilane and styrene in methanol generates appreciable amounts of the hydrosilylation products (Table 2, entry 1), additional NMR studies have been performed. First, the hydrosilylation product **I** was isolated and next tested in the alkoxysilylation reaction in methanol with the cobalt **cat-1** (Scheme 2a). After 16 h, the alkoxylated product was not detected by NMR spectroscopy. This fact means that the alkoxysilanes are first generated via dehydrogenative coupling catalytic reaction between phenylsilane and methanol and then converted via hydrosilylation reaction to the corresponding hydroalkoxysilanes. The poor reactivity of **I** has been tentatively assigned to the presence of the deactivating −CH_2_– group. To confirm this hypothesis, the catalytic dehydrogenative coupling reaction of the commercial dimethylphenylsilane **J** and methanol was carried out at 25 °C with **cat-1**. The dimethylphenylsilane was not converted to the alkoxysilylated product under these reaction conditions after 16 h, corroborating that any aliphatic substitution in the phenylsilane reduces considerably its reactivity (Scheme 2b).

**Scheme 3:**
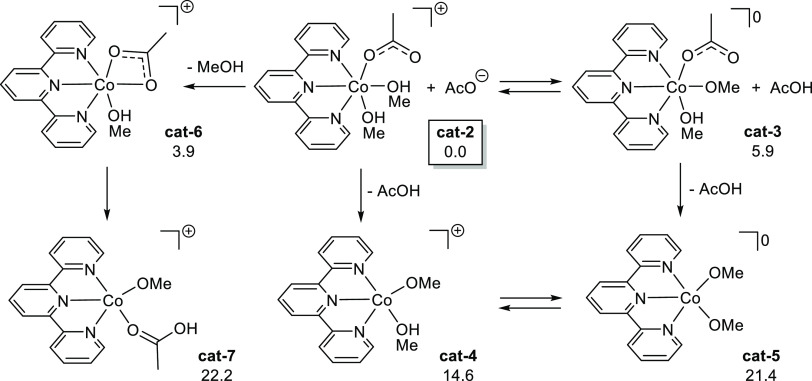
Speciation of Catalyst **cat-1** in Methanol Δ*G*(MeOH) in kcal/mol.

To corroborate the activity trends of silanes, we rely on density functional theory simulations at the B3LYP level with D3 dispersion corrections. From now on, we report Gibbs free energies in methanol in kcal/mol. First, we inspect the speciation of **cat-1** (Scheme 3). In methanol solution, we assume a facile conversion of **cat-1** into **cat-2**, which is then taken as the energy reference. Cationic **cat-2** can convert into neutral **cat-3** at 5.9 kcal/mol via proton transfer. However, these species present six-coordinate Co centers, and an open coordination site is necessary for them to enter in the catalytic cycle. The removal of AcOH from their coordination sphere generates cationic **cat-4** at 14.6 kcal/mol and neutral **cat-5** at 21.4 kcal/mol. For both coordination arrangements, the cationic species are favored over the neutral ones by 6–7 kcal/mol. We further investigate the removal of one methanol molecule from **cat-2** to form the six-coordinate **cat-6** followed by an intramolecular deprotonation yielding the five-coordinate **cat-7**, with a Gibbs energy of 22.2 kcal/mol. Overall, looking at the energies of five-coordinate species, we consider **cat-4** as the most feasible active species, and we will use it for further calculations.

**Figure 2:**
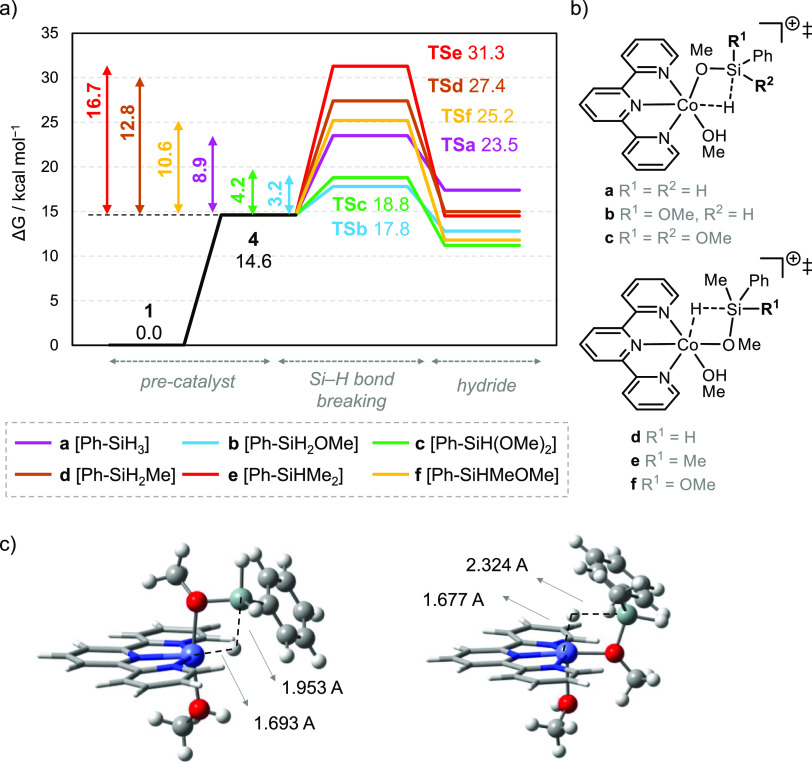
(a) Gibbs energy profile of hydride formation, (b) transition states of the Si–H bond breaking step for several phenylsilane substrates, and (c) 3D models for **TSa** and **TSd**.

Once the potential catalytic species **cat-4** is identified, we now explore its reactivity toward several phenylsilanes. For low-valent metals, the oxidative cleavage of the Si–H bond is feasible.^43,44^ However, for our Co(II) complex, all attempts to find such a process were unsuccessful.^24^ Instead, we propose the participation of five-coordinate MeO–[Si] – transition states that activate the Si–H bond.^45,46^ The intermediate evolves toward the corresponding Co–hydride, which is then quenched by the protic media and releases H_2_. Herein, we focus on the first part, the cleavage of the Si–H bond in different substrates: PhSiH_3_ (**a**), PhSiH_2_OMe (**b**), PhSiH(OMe)_2_ (**c**), PhSiH_2_Me (**d**), PhSiHMe_2_ (**e**), and PhSiHMe(OMe) (**f**). Figure 2 shows the Gibbs energy profile for each substrate and their corresponding transition states. We note that for **a**–**c**, the most stable transition states (TSs) are those where the hydrogen being transferred is *cis* to MeOH; however, these structures could not be found for **d**–**f**, and thus, the TSs present the transferred hydrogen *trans* to MeOH (Figure 2b). The relative activation barrier for the original silane **a** is 8.9 kcal/mol with an overall barrier of 23.5 kcal/mol.^47^ The presence of one and two MeO groups in the silane, **b** and **c**, decreases the relative barriers down to 3.2 and 4.2 kcal/mol, respectively. This is in line with the increased activity of methoxy-substituted phenylsilanes (Figure 1). The inclusion of one and two Me groups in **d** and **e** increases the relative barriers up to 12.8 and 16.7 kcal/mol, respectively. However, when considering the pre-catalyst activation step, these processes are quite demanding with an overall barrier of 27–30 kcal/mol. That is, access to the hydride is hindered and the reaction would not take place under mild conditions, in agreement with the lack of reactivity of aliphatic-substituted substrates (Scheme 2). Finally, the presence of both Me and MeO is also considered in **f**, resulting in a relative barrier of 10.6 kcal/mol and an overall barrier of 25.2 kcal/mol. Substrate **f** seems slightly more reactive than methylated **d** and **e** but still significantly less reactive than methoxylated **b** and **c**.

Once we understand the relative activity trends of silanes, we next target the identification of the cobalt active species by means of EPR, NMR, and Raman spectroscopy. In the EPR at 100 K, **cat-1** was measured in methanol solution and after successive addition of different silane molecules (PhSiH_3_, Ph_2_SiH_2_, (EtO)_3_SiH, and Me_2_PhSiH) over time. The observed g value for **cat-1** in methanol (*g* = 2.09) is consistent with a low spin homoleptic complex [Co(tpy)_2_]^2+^ (*S* = 1/2)^48^ which is in equilibrium with the reported high spin cobalt complex [Co(tpy)(H_2_O)OAc]^+^ (*S* = 3/2), which is silent in EPR.^36,49,50^ When the silane molecule was added to the reaction media, the EPR signal was decreasing over time (Figure S5), indicating that a new high spin cobalt specie *S* = 3/2 (EPR silent) is being formed. This time depends on the silane nature, PhSiH_3_ being the fastest and Me_2_PhSiH the slowest, which needs more than 24 h to start reacting, even in stoichiometric amounts.

This fact has been corroborated by ^1^H-NMR where, in a first instance, it could be seen both groups of signals, corresponding to [Co(tpy)_2_]^2+^ and [Co(tpy)(H_2_O)OAc]^+^ in methanol solution (Figure S1). Starting with Ph_2_SiH_2_, when 1 equiv of this molecule is added to the catalyst solution, the signals assigned to homoleptic complex disappear, and some new signals start to appear (Figure S6). When another equivalent of Ph_2_SiH_2_ is added to the reaction media, the new signals became higher and the ones corresponding to **cat-1** start to decrease. After that, when 2 more equivalents of Ph_2_SiH_2_ are added to the reaction media, only the signals which correspond to the new cobalt species are present in the NMR spectrum (Figure S6). This new species remains in the reaction media after 30 min but, after 24 h, when the cobalt intermediate disappears, only the signals from **cat-1** are present in the NMR spectrum, and there is no trace of the signals belonging to the homoleptic complex (Figure S7). Moreover, acetic acid is detected in the NMR spectrum, which supports the speciation of catalyst **cat-1** in methanol shown in Scheme 3. Interestingly, the cobalt intermediate has been identified by electrospray ionization mass spectrometry (ESI-MS) as [Co(CH_3_O)(H_2_O)(Ph_2_SiH_2_)(tpy)]^+^ (*m/z* = 525.1216) when 10 equiv of Ph_2_SiH_2_ are added to **cat-1** solution in methanol (Figure S10). The same behavior is observed when (EtO)_3_SiH is added instead of Ph_2_SiH_2_ (Figure S8). However, for Me_2_PhSiH, we have to wait 24 h until the signals of the new cobalt species appear (Figure S9), which indicates that the Si–H bond is less activated when bearing alkyl groups.

In addition, in situ Raman spectroscopy was employed to study the intermediate Co species formed during the reaction between the catalyst and the corresponding silane. First, **cat-1** in methanol and different silanes were measured separately. Then, solutions of **cat-1** after the addition of different silanes were studied. After the addition of PhSiH_3_ or Ph_2_SiH_2_, bubbles are observed, corresponding to hydrogen formation. The catalytic reaction with these two silanes with 0.01% of catalyst is very fast, so, when the catalyst/silane ratio is 1:10, the process is more exothermic, in addition to heating due to the Raman laser irradiation. In order to make the process less exothermic and slower, (EtO)_3_SiH was used instead. Under these conditions, a strong band at 2256 cm^–1^ was detected (Figure S11 in green), and this band was clearly shifted with respect to the Si–H bond of the silane (Figure S11 in red). This signal was tentatively assigned to monodentate coordination of the silane to Co via OMe (see Scheme S1).^50,51^ The in situ experiment with Me_2_PhSiH results in a mixture of signals of **cat-1** and Me_2_PhSiH, and the intermediate species could not be detected due to the slowness of the reaction with this silane as has been proved through EPR and NMR spectroscopy (Figure S12).

### Proposed Mechanistic Pathway for the Hydroalkoxysilylation Reaction with **cat-1**

**Scheme 4:**
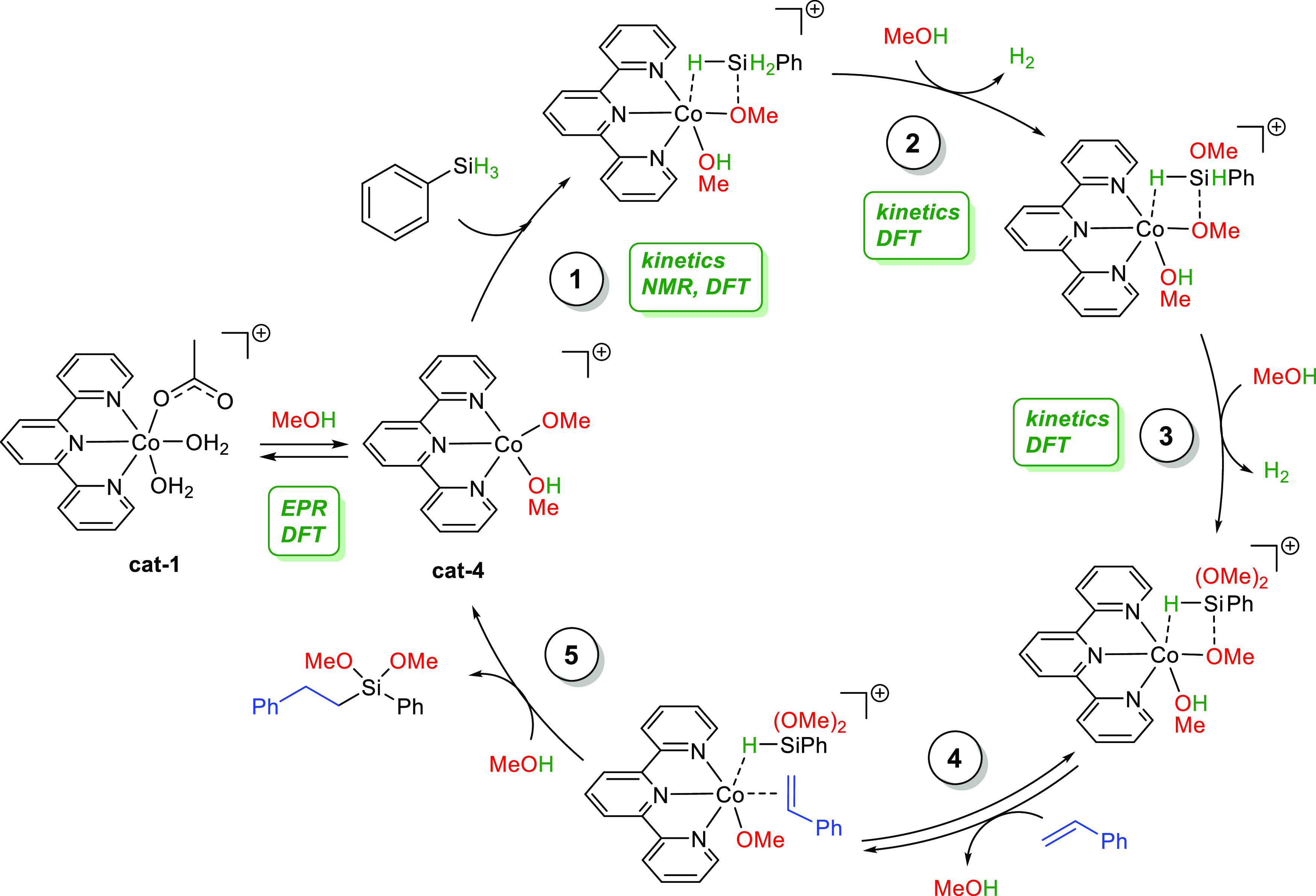
Hydroalkoxysilylation Reaction of Phenylsilane and Styrene in Methanol Catalyzed By **cat-1** (1) Activation of the Si–H, (2) protonation of the Co-hydride transition state and release of a hydrogen molecule, (3) production of a second hydrogen molecule, (4) coordination of alkene to the cobalt center, and (5) hydrosilylation reaction.

With all the information obtained from in situ and ex situ experiments and computational studies, we propose a feasible catalytic route for the hydroalkoxysilylation reaction of phenylsilane and styrene in methanol catalyzed by **cat-1** (Scheme 4): (1) The first step is the activation of the Si–H bond via a five-coordinated complex and its subsequent cleavage mediated by the Co-catalyst. (2) The Co-hydride transition state generated in the first step is protonated by MeOH, releasing a hydrogen molecule. At this point, another MeOH enters in the coordination sphere of the Co atom. (3) A second hydrogen molecule is produced in the same way as that in step 2. (4) The double bond of styrene is coordinated to the Co center. (5) Finally, the partially negative charge induced by the methoxy groups to the silicon facilitates the hydrosilylation reaction of the dimethoxyphenylsilane intermediates and the styrene. The appreciable detected amount of the hydrosilylation product of styrene and phenylsilane is due to the competition of the hydrosilylation and the alkoxysilylation reactions. Hydrosilylation could take place from the intermediate formed in step 2, with the consequent formation of the monomethoxylated product. However, according to simulations, the second methoxylation of phenylsilane is significantly faster than the first one (**a** vs **b** in Figure 2) and would likely occur before the hydrosilylation step.

### Coupled Production of Green Energy and an Industrially Relevant Silicon Precursor

**Scheme 5:**
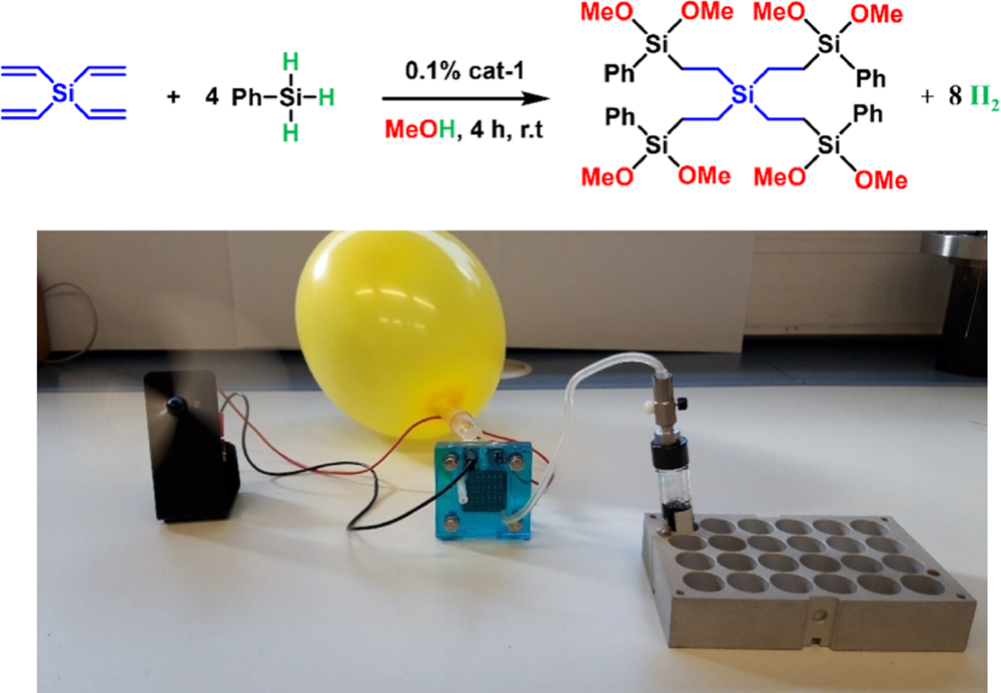
Hydroalkoxysilylation Reaction of Tetravinylsilane and Phenylsilane To Obtain a Silicon Precursor (Top) and a Picture of Energy Generation via Recombination of Hydrogen Obtained in the Hydroalkoxysilylation Reaction and Oxygen Balloon in a Fuel Cell (Bottom)

In order to check the real applicability of this process with **cat**-**1**, we perform the hydroalkoxysilylation of tetravinylsilane via one-pot catalytic dehydrogenative coupling of PhSiH_3_ with methanol. The resulting product is a potential precursor for the construction of silicon-based materials (Scheme 5). At the same time that the silicon precursor is being generated, H_2_ is being produced on demand at a high pressure. Therefore, from an atom-economy perspective, this is a circular approach where both products have powerful potential. In addition, from a sustainable point of view, this process has been achieved in green protic solvents. To demonstrate the added value of this hydrogen production, the catalytic reactor was connected to the anode of a fuel cell while an oxygen balloon was connected to the cathode. The green energy obtained from the combination of hydrogen and oxygen was able to move a fan motor (Scheme 5), although this is not its unique application. Altogether, this experiment shows that this sustainable methodology is a feasible and effective way to obtain a high-value product coupled with green energy following circular economy rules.

## Conclusions

In summary, we have developed an efficient Co-based catalytic system for the synthesis of alkoxysilanes coupled with the production of green hydrogen on demand under mild conditions and low metal loading. This air- and moisture-stable catalyst can be easily prepared from available starting materials at the industrial scale, which is pivotal when employing green solvents. This new sustainable methodology can be applied to the one-pot synthesis of alkoxysilanes in high yields, using different hydrosilanes and terminal alkenes as starting materials. The selectivity of the process, from hydroalkoxysilylation to hydrosilylation products, can be controlled by tuning the water content in the alcohol media. Experimental, in situ, and computational studies confirm that the one-pot reaction proceeded via dehydrogenative coupling reaction followed by hydrosilylation reaction between the alkene and the alkoxysilane intermediates generated in the first step. Further, EPR, NMR, Raman, and ESI-MS studies allowed us to detect a cobalt intermediate after the coordination of silane molecules. Overall, this work opens a new route to the synthesis of industrially relevant polymer precursors coupled with the hydrogen production at high pressures as byproducts, following circular economy rules and using cheap and stable Co-based catalysts.

## Supporting Information Available

Experimental procedures employed during this study, in situ, kinetic, and mechanistic study data and characterization of catalytic products (PDF)

## References

[ref1] Salehabadi A.; Umar M. F.; Ahmad A.; Ahmad M. I.; Ismail N.; Rafatullah M. Carbon-Based Nanocomposites in Solid-State Hydrogen Storage Technology: An Overview. Int. J. Energy Res. 2020, 44, 11044–11058. 10.1002/er.5674.

[ref2] Olabi A. G.; Bahri A. S.; Abdelghafar A. A.; Baroutaji A.; Sayed E. T.; Alami A. H.; Rezk H.; Abdelkareem M. A. Large-Vscale Hydrogen Production and Storage Technologies: Current Status and Future Directions. Int. J. Hydrogen Energy 2021, 46, 23498–23528. 10.1016/j.ijhydene.2020.10.110.

[ref3] Crabtree R. H. Hydrogen Storage in Liquid Organic Heterocycles. Energy Environ. Sci. 2008, 1, 134–138. 10.1039/b805644g.

[ref4] Yadav M.; Xu Q. Liquid-Phase Chemical Hydrogen Storage Materials. Energy Environ. Sci. 2012, 5, 9698–9725. 10.1039/c2ee22937d.

[ref5] He T.; Pei Q.; Chen P. Liquid Organic Hydrogen Carriers. J. Energy Chem. 2015, 24, 587–594. 10.1016/j.jechem.2015.08.007.

[ref6] Shukla A.; Karmakar S.; Biniwale R. B. Hydrogen Delivery through Liquid Organic Hydrides: Considerations for a Potential Technology. Int. J. Hydrogen Energy 2012, 37, 3719–3726. 10.1016/j.ijhydene.2011.04.107.

[ref7] Biniwale R. B.; Rayalu S.; Devotta S.; Ichikawa M. Chemical Hydrides: A Solution to High Capacity Hydrogen Storage and Supply. Int. J. Hydrogen Energy 2008, 33, 360–365. 10.1016/j.ijhydene.2007.07.028.

[ref8] Kariya N.; Fukuoka A.; Ichikawa M. Efficient Evolution of Hydrogen from Liquid Cycloalkanes over Pt-Containing Catalysts Supported on Active Carbons under “Wet-Dry Multiphase Conditions.”. Appl. Catal. A Gen. 2002, 233, 91–102. 10.1016/S0926-860X(02)00139-4.

[ref9] H-atom E.; C-n T.; Hcn T.; Sutton A. D.; Burrell A. K.; Dixon D. A.; Garner E. B.; Gordon J. C.; Nakagawa T.; Ott K. C.; Robinson J. P.; Vasiliu M. Regeneration of Ammonia Borane. Science 2011, 331, 1426–1429. 10.1126/science.1199003. 21415349

[ref10] Loges B.; Boddien A.; Gärtner F.; Junge H.; Beller M. Catalytic Generation of Hydrogen from Formic Acid and Its Derivatives: Useful Hydrogen Storage Materials. Top. Catal. 2010, 53, 902–914. 10.1007/s11244-010-9522-8.

[ref11] Joó F. Breakthroughs in Hydrogen Storage\-Formic Acid as a Sustainable Storage Material for Hydrogen. ChemSusChem 2008, 1, 805–808. 10.1002/cssc.200800133. 18781551

[ref12] Fellay C.; Dyson P. J.; Laurenczy G. A Viable Hydrogen-Storage System Based on Selective Formic Acid Decomposition with a Ruthenium Catalyst. Angew. Chem., Int. Ed. 2008, 47, 3966–3968. 10.1002/anie.200800320. 18393267

[ref13] Marder T. B. Will We Soon Be Fueling Our Automobiles with Ammonia-Borane?. Angew. Chem., Int. Ed. 2007, 46, 8116–8118. 10.1002/anie.200703150. 17886317

[ref14] Hamilton C. W.; Baker R. T.; Staubitz A.; Manners I. B–N Compounds for Chemical Hydrogen Storage. Chem. Soc. Rev. 2009, 38, 279–293. 10.1039/b800312m. 19088978

[ref15] Stephens F. H.; Pons V.; Tom Baker R. Ammonia–Borane: The Hydrogen Source Par Excellence?. Dalton Trans. 2007, 2, 2613–2626. 10.1039/b703053c. 17576485

[ref16] Clot E.; Eisenstein O.; Crabtree R. H. Computational Structure-Activity Relationships in H2 Storage: How Placement of N Atoms Affects Release Temperatures in Organic Liquid Storage Materials. Chem. Commun. 2007, 22, 2231–2233. 10.1039/b705037b. 17534500

[ref17] Grasemann M.; Laurenczy G. Formic Acid as a Hydrogen Source - Recent Developments and Future Trends. Energy Environ. Sci. 2012, 5, 8171–8181. 10.1039/c2ee21928j.

[ref18] Singh A. K.; Singh S.; Kumar A. Hydrogen Energy Future with Formic Acid: A Renewable Chemical Hydrogen Storage System. Catal. Sci. Technol. 2016, 6, 12–40. 10.1039/c5cy01276g.

[ref19] Mellmann D.; Sponholz P.; Junge H.; Beller M. Formic Acid as a Hydrogen Storage Material-Development of Homogeneous Catalysts for Selective Hydrogen Release. Chem. Soc. Rev. 2016, 45, 3954–3988. 10.1039/c5cs00618j. 27119123

[ref20] Aliaga-Lavrijsen M.; Iglesias M.; Cebollada A.; Garcés K.; García N.; Sanz Miguel P. J.; Fernández-Alvarez F. J.; Pérez-Torrente J. J.; Oro L. A. Hydrolysis and Methanolysis of Silanes Catalyzed by Iridium(III) Bis-N-Heterocyclic Carbene Complexes: Influence of the Wingtip Groups. Organometallics 2015, 34, 2378–2385. 10.1021/om5011726.

[ref21] Staubitz A.; Robertson A. P. M.; Manners I. Ammonia-Borane and Related Compounds as Dihydrogen Sources. Chem. Rev. 2010, 110, 4079–4124. 10.1021/cr100088b. 20672860

[ref22] Sorribes I.; Ventura-Espinosa D.; Assis M.; Martín S.; Concepción P.; Bettini J.; Longo E.; Mata J. A.; Andrés J. Unraveling a Biomass-Derived Multiphase Catalyst for the Dehydrogenative Coupling of Silanes with Alcohols under Aerobic Conditions. ACS Sustainable Chem. Eng. 2021, 9, 2912–2928. 10.1021/acssuschemeng.0c08953.

[ref23] Ventura-Espinosa D.; Sabater S.; Carretero-Cerdán A.; Baya M.; Mata J. A. High Production of Hydrogen on Demand from Silanes Catalyzed by Iridium Complexes as a Versatile Hydrogen Storage System. ACS Catal. 2018, 8, 2558–2566. 10.1021/acscatal.7b04479.

[ref24] Ventura-Espinosa D.; Carretero-Cerdán A.; Baya M.; García H.; Mata J. A. Catalytic Dehydrogenative Coupling of Hydrosilanes with Alcohols for the Production of Hydrogen On-Demand: Application of a Silane/Alcohol Pair as a Liquid Organic Hydrogen Carrier. Chem. Eur. J. 2017, 23, 10815–10821. 10.1002/chem.201700243. 28745407

[ref25] Schuster C. H.; Diao T.; Pappas I.; Chirik P. J. Bench-Stable, Substrate-Activated Cobalt Carboxylate Pre-Catalysts for Alkene Hydrosilylation with Tertiary Silanes. ACS Catal. 2016, 6, 2632–2636. 10.1021/acscatal.6b00304.

[ref26] Brook M. A. Silicon in Organic, Organometallic, and Polymer Chemistry; Wiley, 2000; 123.

[ref27] Ojima I. The Chemistry of Organic Silicon Compounds. In The Chemistry of Organic Silicon Compounds; John Wiley & Sons, Ltd, 1989, 1479.

[ref28] Eduok U.; Faye O.; Szpunar J. Recent Developments and Applications of Protective Silicone Coatings: A Review of PDMS Functional Materials. Prog. Org. Coat. 2017, 111, 124–163. 10.1016/j.porgcoat.2017.05.012.

[ref29] Yongzhao X.; Kundu X. H. S.; Nag A.; Afsarimanesh N.; Sapra S.; Mukhopadhyay S. C.; Silicon-Based T. H. Sensors for Biomedical Applications. Sensors 2019, 19, 2908–2930. 10.3390/s19132908. 31266148 PMC6651638

[ref30] Speier J. L.; Webster J. A.; Barnes G. H. The Addition of Silicon Hydrides to Olefinic Double Bonds. Part II. The Use of Group VIII Metal Catalysts. J. Am. Chem. Soc. 1957, 79, 974–979. 10.1021/ja01561a054.

[ref31] Karstedt B. D. Platinum-Vinylsiloxanes, 1973.

[ref32] Tamang S. R.; Findlater M. Emergence and Applications of Base Metals (Fe, Co, and Ni) in Hydroboration and Hydrosilylation. Molecules 2019, 24, 3194 10.3390/molecules24173194. 31484333 PMC6749197

[ref33] Atienza C. C. H.; Diao T.; Weller K. J.; Nye S. A.; Lewis K. M.; Delis J. G. P.; Boyer J. L.; Roy A. K.; Chirik P. J. Bis(Imino)Pyridine Cobalt-Catalyzed Dehydrogenative Silylation of Alkenes: Scope, Mechanism, and Origins of Selective Allylsilane Formation. J. Am. Chem. Soc. 2014, 136, 12108–12118. 10.1021/ja5060884. 25068530

[ref34] Docherty J. H.; Peng J.; Dominey A. P.; Thomas S. P. Activation and Discovery of Earth-Abundant Metal Catalysts Using Sodium Tert-Butoxide. Nat. Chem. 2017, 9, 595–600. 10.1038/nchem.2697. 28537588

[ref35] Chirik P. J.; Tondreau A. M.; Delis J. G. P.; Lewis K. M.; Weller K. J.; Nye S. A. In-Situ Activation of Metal Complexes Containing Terdentate Nitrogen Ligands Used as Hydrosilylation Catalysts. 2014.

[ref36] Gutiérrez-Tarriño S.; Concepción P.; Oña-Burgos P. Cobalt Catalysts for Alkene Hydrosilylation under Aerobic Conditions without Dry Solvents or Additives. Eur. J. Inorg. Chem. 2018, 2018, 4867–4874. 10.1002/ejic.201801068.

[ref37] Yuan W.; Orecchia P.; Oestreich M. Palladium-Catalyzed Three-Component Reaction of Dihydrosilanes and Vinyl Iodides in the Presence of Alcohols: Rapid Assembly of Silyl Ethers of Tertiary Silanes. Chem. Eur. J. 2018, 24, 19175–19178. 10.1002/chem.201805595. 30431674

[ref38] Obligacion J. V.; Chirik P. J. Earth-Abundant Transition Metal Catalysts for Alkene Hydrosilylation and Hydroboration. Nat. Rev. Chem. 2018, 2, 15–34. 10.1038/s41570-018-0001-2. 30740530 PMC6365001

[ref39] Pappas I.; Treacy S.; Chirik P. J. Alkene Hydrosilylation Using Tertiary Silanes with α-Diimine Nickel Catalysts. Redox-Active Ligands Promote a Distinct Mechanistic Pathway from Platinum Catalysts. ACS Catal. 2016, 6, 4105–4109. 10.1021/acscatal.6b01134.

[ref40] Noda D.; Tahara A.; Sunada Y.; Nagashima H. Non-Precious-Metal Catalytic Systems Involving Iron or Cobalt Carboxylates and Alkyl Isocyanides for Hydrosilylation of Alkenes with Hydrosiloxanes. J. Am. Chem. Soc. 2016, 138, 2480–2483. 10.1021/jacs.5b11311. 26760915

[ref41] Kim B. H.; Cho M. S.; Woo H. G. Si-Si/Si-C/Si-O/Si-N Coupling of Hydrosilanes to Useful Silicon-Containing Materials. Synlett 2004, 5, 761–772. 10.1055/s-2004-820016.

[ref42] Dong X.; Weickgenannt A.; Oestreich M. Broad-Spectrum Kinetic Resolution of Alcohols Enabled by Cu-H-Catalysed Dehydrogenative Coupling with Hydrosilanes. Nat. Commun. 2017, 8, 15547 10.1038/ncomms15547. 28569754 PMC5461486

[ref43] Gandon V.; Agenet N.; Vollhardt K. P. C.; Malacria M.; Aubert C. Silicon-Hydrogen Bond Activation and Hydrosilylation of Alkenes Mediated by CpCo Complexes: A Theoretical Study. J. Am. Chem. Soc. 2009, 131, 3007–3015. 10.1021/ja809100t. 19209851

[ref44] Ma Y.; Han Z. Computation Revealed Mechanistic Complexity of Low-Valent Cobalt-Catalyzed Markovnikov Hydrosilylation. J. Org. Chem. 2018, 83, 14646–14657. 10.1021/acs.joc.8b02455. 30418773

[ref45] Raya-Barón Á.; Ortuño M. A.; Oña-Burgos P.; Rodríguez-Diéguez A.; Langer R.; Cramer C. J.; Kuzu I.; Fernández I. Efficient Hydrosilylation of Acetophenone with a New Anthraquinonic Amide-Based Iron Precatalyst. Organometallics 2016, 35, 4083–4089. 10.1021/acs.organomet.6b00765.

[ref46] Voronova E. D.; Golub I. E.; Pavlov A.; Belkova N. V.; Filippov O. A.; Epstein L. M.; Shubina E. S. Dichotomous Si-H Bond Activation by Alkoxide and Alcohol in Base-Catalyzed Dehydrocoupling of Silanes. Inorg. Chem. 2020, 59, 12240–12251. 10.1021/acs.inorgchem.0c01293. 32805120

[ref47] NOTE: A value of 23.5 kcal/mol computed at the B3LYP-D3 level is relatively high for the experimental conditions. Calculations at the PBE0-D3 level indeed yield a lower barrier of 20.3 kcal/mol. Thus, although the present absolute values may be overestimated, the discussion on the relative energy barriers between silane substrates is still valid.

[ref48] Mizuno K.; Imamura S.; Lunsford J. H. EPR Study of [CoIIL2]2+, [CoIILL’]2+, and [CoIIILL’O2]2+ (L = 2,2’,2”-Terpyridine; L’ = 2,2’-Bypyridine) Complexes in Zeolite Y. Inorg. Chem. 1984, 23, 3510–3514. 10.1021/ic00190a015.

[ref49] Cibian M.; Hanan G. S. Geometry and Spin Change at the Heart of a Cobalt(II) Complex: A Special Case of Solvatomorphism. Chem. Eur. J. 2015, 21, 9474–9481. 10.1002/chem.201500852. 25899499

[ref50] Palma A.; Gallagher J. F.; Müller-Bunz H.; Wolowska J.; McInnes E. J. L.; O’Shea D. F. Co(II), Ni(II), Cu(II) and Zn(II) Complexes of Tetraphenylazadipyrromethene. Dalton Trans. 2009, 2, 273–279. 10.1039/b811764k. 19089007

[ref51] Sokrates G. Infrared and Raman Characteristic Group Frequencies: Tables and Charts; Wiley, 2004.

