# Supplementary material for: Sustainable Synthesis of Silicon Precursors Coupled with Hydrogen Delivery Based on Circular Economy via Molecular Cobalt-Based Catalysts
Source: ACS Sustain Chem Eng. 2022 Dec 8;10(50):16624–33. doi: 10.1021/acssuschemeng.2c04444 (PMC9940298; doi:10.1021/acssuschemeng.2c04444)
Supplement: Supplementary file 1 — sc2c04444_si_001.pdf [file sc2c04444_si_001.pdf]

*Supporting Information*

# Sustainable synthesis of silicon precursors coupled with hydrogen delivery based on circular economy via molecular cobalt-based catalysts


Silvia Gutiérrez-Tarriño,[a] Sergio Rojas-Buzo,[a,b] Manuel A. Ortuño[c] and Pascual Oña-Burgos[a,d]*

[a]*Instituto de Tecnología Química, Universitat Politècnica de València-Consejo Superior de Investigaciones Científicas (UPV-CSIC), Avda. de los Naranjos s/n, 46022 Valencia, Spain.*

[b]*Department of Chemistry, NIS and INSTM Reference Centre, Università di Torino, 10125, Italy.*

[c]*Centro Singular de Investigación en Química Biolóxica e Materiais Moleculares (CIQUS), Universidade de Santiago de Compostela, 15782 Santiago de Compostela, Spain.*

[d]*Department of Chemistry and Physics, Research Centre CIAIMBITAL, University of Almería, Ctra. Sacramento, s/n, 04120 Almería, Spain.*


Number of pages: 41

Number of figures: 51

Number of schemes: 1

Number of tables: 1

**Table of contents**

I. General Information .......... S3

II. Experimental procedures .......... S4

A. Synthesis and Characterization of Cobalt coordination compounds. .......... S4

B. General Procedure for Catalytic Hydrosilylation Reactions .......... S5

C. Substrate screening for the one-pot reaction. .......... S6

D. Synthesis and Characterization of Silane Products .......... S7

III. In situ, kinetic and mechanistic studies. .......... S11

A. Kinetic studies .......... S11

B. Isotopic effect: NMR detection of $H_2$/HD .......... S12

C. EPR Spectroscopy .......... S12

D. RMN Spectroscopy .......... S13

E. ESI-MS .......... S17

F. Raman Spectroscopy .......... S18

IV. NMR Spectra of Silane products. .......... S20

V. References .......... S40

## I. General Information

**Experimental details.** All reagents and solvents were purchased from commercial suppliers and used without further purification. New hydrosilylation products obtained were characterized by GC-MS, $^{1}$H, $^{13}$C-NMR and DEPT. When available, characterization given in the literature was used for comparison. Isolated catalyst was characterized by $^{1}$H-NMR, elemental analysis, ICP and ESI-MS and the obtained results were compared with the reported ones.[1] C, N and H contents of isolated catalysts were determined with a Carlo Erba 1106 elemental analyzer and cobalt content of isolated catalysts were determined with a Varian 715-ES by inductively coupled plasma spectroscopy. $^{1}$H and $^{13}$C NMR were recorded on a Bruker 300 spectrometer and the chemical shifts are reported in ppm relative to residual proton solvents signals. Data for $^{1}$H NMR spectra are reported as follows: chemical shift (δ, ppm), multiplicity (s = singlet, d = doublet, t = triplet, q = quartet, m = multiplet, dd = double doublets) and integration. Data for $^{13}$C NMR spectra are reported in chemical shift (δ, ppm). Exact mass values were determined by using a Waters ACQUITY™ XevoQToF spectrometer (Waters Corp.) connected to the UPLC system via electrospray ionization (ESI) interface. The ESI source was operated in positive ionization mode with the capillary voltage at 3.0 kV. The temperature of the source and desolvation was set at 120 °C and 400 °C, respectively. The cone and desolvation gas flows were 10 L $h^{-1}$ and 800 L $h^{-1}$, respectively. All data collected in Centroid mode were acquired using Masslynx™ software (Waters Corp.). Leucine-enkephalin was used as the lock mass generating an $[M+H]^{+}$ ion (*m/z* 556.2771) at a concentration of 500 pg/mL and a flow rate of 20 μL/min to ensure accuracy during the MS analysis. EPR spectra were recorded at 100 K on a Bruker EMX-12 instrument operating in X band at 9.5 GHz, modulation amplitude of 1 G and modulation frequency of 100 KHz. Raman spectra were recorded with 514 and 785 nm laser excitation on a Renishaw in via Raman Spectrometer (“Refelx”) equipped with a CCD detector. The laser power on the sample was between 5 and 50% and a total of 20 acquisitions were taken for each spectrum.

**Computational details.** All calculations were performed at density functional theory (DFT) level using the B3LYP density functional[2–4] and D3 dispersion corrections[5] as implemented in Gaussian 16. The continuum solvent model IEFPCM was used to describe the methanol medium.[6] Co atoms were described with an effective core potential SDD together with its associated double-ζ basis set[7] and a set of f-polarization functions[8]. The 6-31+G(d,p) was used for H atoms, 6-31+G(d) for Si, N, and C atoms, and 6-31+G(d) for O atoms (BS-1).[9,10] In these Co(II) complexes, the quartet spin state is typically more stable than the doublet one. Only for the cobalt–hydride species both spin states are similar in energy. Geometry optimizations were performed without any symmetry restrictions and analytical frequency calculations were computed to confirm

minima (intermediates) and maxima (transition states, one imaginary frequency). Gibbs energies were computed at 298.15 K and 1 M. All frequencies below 50 $cm^{-1}$ were replaced by 50 $cm^{-1}$ when computing vibrational partition functions[11] with the Goodvibes script.[12] Single point calculations were performed on previous optimized geometries using the larger basis set 6-311+G(d,p) for all atoms (BS-2).[13–15] Final Gibbs energies are obtained as follows:

$$\Delta G = \Delta E(BS\text{-}2) + [\Delta G(BS\text{-}1) - \Delta E(BS\text{-}1)]$$

All inputs and outputs can be obtained from the open access database ioChem-BD[16] in the following database.[17]

## II. Experimental procedures

### A. Synthesis and Characterization of Cobalt coordination compounds.

**Preparation of [Co(OAc)$_2$(tpy)] freshly.** 5.5 mg of terpyridine (0.022 mmol) and 5.2 mg of Cobalt (II) acetate tetrahydrate (0.022 mmol) was dissolved in 5 mL of methanol, ethanol or a mixture of one of these alcohols with water (1:1) and stirred for 1 hour.

This freshly solution is used for the catalytic reaction, but the complex has been also isolated in order to clearly characterized the formed complex, both in solid and in solution.

**Preparation of [Co(OAc)($H_2O$)$_2$(tpy)]OAc (cat-1).** 100 mg of terpyridine (0.428 mmol) and 106.6 mg of Co(II) acetate tetrahydrate (0.428 mmol) were dissolved in 50 mL of a mixture of acetonitrile:chloroform (1:1) with some drops of methanol according to the reported procedure.[1] An immediate color change to orange was observed. Single crystals were obtained by slow evaporation of the solvent. Anal. For $C_{19}H_{21}CoN_3O_6$: calc. = C, 51.13; H, 4.71; N, 9.42. Found = C, 51.048; H, 4.667; N, 9.543. The data are in accordance with the literature.[1]

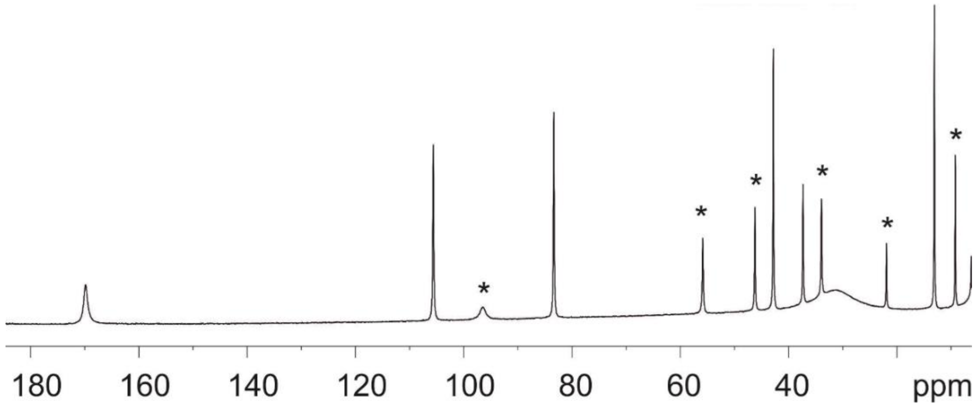


**Figure S1.** $^1$H-NMR of **[Co(OAc)$_2$(tpy)] freshly (cat-1)** where (*) represents the complex with two tpy ligands [Co(tpy)$_2$].

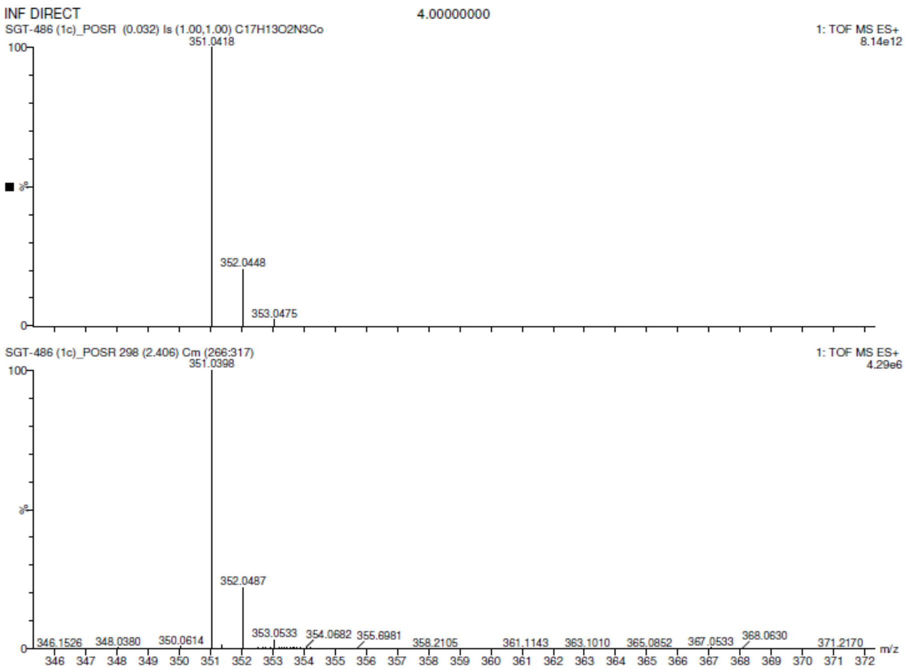


**Figure S2.** Theoric ESI spectrum for the formula $C_{17}H_{13}N_3O_2Co$ (above) and experimental ESI spectrum of isolated **cat-1** which correspond with the formula $C_{17}H_{13}N_3O_2Co$ (below).

## B. General Procedure for Catalytic Hydrosilylation Reactions

0.2 mL of freshly catalyst solution (0.001 equiv.) were taken and added to a vial equipped with a stir bar, followed by olefin (0.89 mmol, 1 equiv.) and silane (0.89 mmol, 1 equiv.) resulting in formation of a dark reaction mixture. The vial was sealed with a cap and stirred at room temperature. The catalyst was removed from the reaction media by precipitating it with hexane. The solvent was evaporated and an aliquot was analyzed by $^1$H NMR in $CDCl_3$. Attempts to purify some products, passing through a small column of silica gel, resulted in decomposition with the formation of dehydrogenated products.

The catalyst loading has been optimized. In this sense, 0.05% and 0.01% catalyst loadings have been tested. In both cases, the reaction takes place, but the reaction rate slows down. So, we have chosen 0.1% of catalyst as optimum catalyst loading.

## C. Substrate screening for the one-pot reaction.

**Table S1.** Evaluation of **cat-1** for the hydrosilylation/alkoxysilylation of alkenes with $Ph_2SiH_2$.[a]

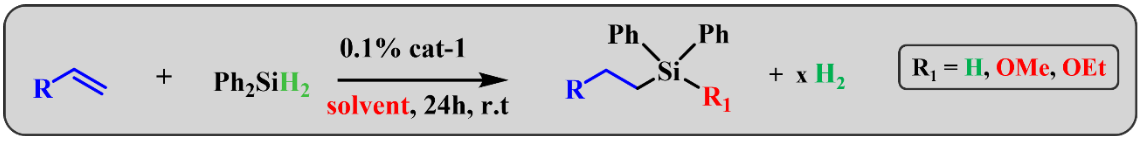


| Entry | Substrate | Solvent | Product | Conversion (%)[b] | Selectivity (%) |
|---|---|---|---|---|---|
| 2a | | MeOH | 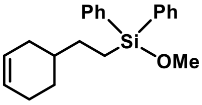 | > 99 | 95 |
| 2b | 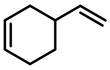 | EtOH | 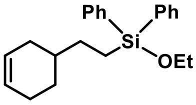 | > 99 | 63 |
| 2c | | MeOH:$H_2O$/ EtOH:$H_2O$ | 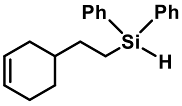 | > 99 | > 99 |
| 2d | | MeOH | 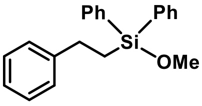 | > 99 | 87 |
| 2e | 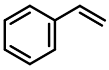 | EtOH | 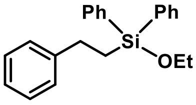 | > 99 | 77 |
| 2f | | MeOH:$H_2O$/ EtOH:$H_2O$ | 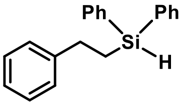 | > 99 | > 99 |
| 2g | | MeOH | 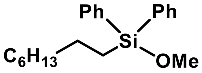 | > 99 | 75 |
| 2h | 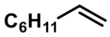 | EtOH | 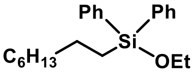 | > 99 | 72 |
| 2i | | MeOH:$H_2O$/ EtOH:$H_2O$ | 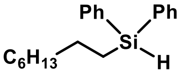 | > 99 | > 99 |

[a] All reactions were performed on 0.89 mmol scale using a 1:1 silane/olefin mixture under aerobic conditions. [b] Conversion of diphenylsilane was determined by $^1$H-NMR analysis of the crude reaction mixture.

**D. Synthesis and Characterization of Silane Products**

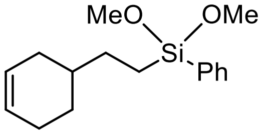


**(2-(cyclohex-3-en-1-yl)ethyl)dimethoxy(phenyl)silane (1a).** Prepared according to the general procedure, using 0.1% mol of catalyst dissolved in methanol, 4-vinylcyclohexene (96.3 mg, 0.89 mmol) and phenylsilane (96.3 mg, 0.89 mmol). The reaction was stirred for 6 hours at room temperature. $^{1}$H NMR (300 MHz, $CDCl_3$) δ = 7.71-7,64 (m, 2H), 7.45-7.40 (m, 3H), 5.68 (s, 2H), 3.67 (s, 6H) 2.05-0.89 (m, 11H). $^{13}$C NMR (75 MHz, $CDCl_3$) δ = 134.79, 134.32, 130.16, 127.96, 127.06, 126.60, 50.85, 36.38, 31.61, 31.52, 28.42, 25.34, 9.33.

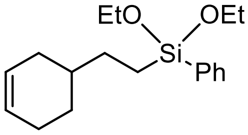


**(2-(cyclohex-3-en-1-yl)ethyl)diethoxy(phenyl)silane (1b).** Prepared according to the general procedure, using 0.1% mol of catalyst dissolved in ethanol, 4-vinylcyclohexene (96.3 mg, 0.89 mmol) and phenylsilane (96.3 mg, 0.89 mmol). The reaction was stirred for 6 hours at room temperature. $^{1}$H NMR (300 MHz, $CDCl_3$) δ = 7.73-7,62 (m, 2H), 7.47-7.42 (m, 3H), 5.69 (s, 2H), 3.96 (q, 4H), 2.07-0.90 (m, 17H). $^{13}$C NMR (75 MHz, $CDCl_3$) δ = 134.75, 134.30, 130.12, 127.93, 127.05, 126.58, 57.98, 36.35, 31.60, 31.48, 28.39, 25.32, 18.20, 9.31.

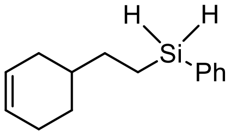


**(2-(cyclohex-3-en-1-yl)ethyl)(phenyl)silane (1c).** Prepared according to the general procedure, using 0.1% mol of catalyst dissolved in methanol/water (1:1) or ethanol/water (1:1), 4-vinylcyclohexene (96.3 mg, 0.89 mmol) and phenylsilane (96.3 mg, 0.89 mmol). The reaction was stirred for 6 hours at room temperature. $^{1}$H NMR (300 MHz, $CDCl_3$) δ = 7.51-7.48 (m, 2H), 7.31-7.28 (m, 3H), 5.58 (s, 2H), 4.22 (t, J = 3.6 Hz, 2H), 1.51-0.86 (m, 11H). $^{13}$C NMR (75 MHz, $CDCl_3$) δ = 135.20, 134.63, 129.51, 127.97, 127.06, 126.52, 36.10, 31.79, 31.52, 28.43, 25.25, 7.17. The spectra are in accordance with the literature.[1]

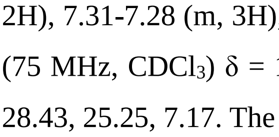


**Dimethoxy(phenethyl)(phenyl)silane (1d).** Prepared according to the general procedure, using 0.1% mol of catalyst dissolved in methanol, styrene (92.7 mg, 0.89 mmol) and phenylsilane (96.3 mg, 0.89 mmol). The reaction was stirred for 6 hours at room temperature. $^{1}$H NMR (300 MHz, $CDCl_3$) δ = 7.57-7.55 (m, 2H), 7.36-7.33 (m, 3H), 7.19-7.15 (m, 2H), 7.12-7.08 (m, 3H), 3.52 (s, 6H), 2.67-2.61 (m, 2H), 1.20-1.12 (m, 2H). $^{13}$C NMR (75 MHz, $CDCl_3$) δ = 144.52, 134.40, 132.80, 130.33, 128.38, 128.06, 127.83, 125.71, 50.75, 28.71, 14.33.

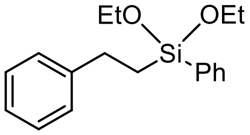


**Diethoxy(phenethyl)(phenyl)silane (1e).**

Prepared according to the general procedure, using 0.1% mol of catalyst dissolved in ethanol, styrene (92.7 mg, 0.89 mmol) and phenylsilane (96.3 mg, 0.89 mmol). The reaction was stirred for 6 hours at room temperature. $^{1}$H NMR (300 MHz, $CDCl_3$) δ = 7.61-7.58 (m, 2H), 7.42-7.38 (m, 3H), 7.23-7.19 (m, 2H), 7.15-7.10 (m, 3H), 3.97 (q, 4H), 2.68-2.63 (m, 2H), 1.36 (t, 6H) 1.24-1.14 (m, 2H). $^{13}$C NMR (75 MHz, $CDCl_3$) δ = 144.54, 134.43, 132.82, 130.36, 128.39, 128.08, 127.85, 125.75, 57.99, 28.73, 18.30, 14.36.

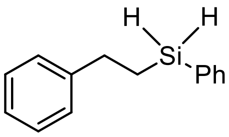


**Phenethyl(phenyl)silane (1f).**

Prepared according to the general procedure, using 0.1% mol of catalyst dissolved in methanol/water (1:1) or ethanol/water (1:1), styrene (92.7 mg, 0.89 mmol) and phenylsilane (96.3 mg, 0.89 mmol). The reaction was stirred for 6 hours at room temperature. $^{1}$H NMR (300 MHz, $CDCl_3$): δ = 7.65-7.52 (m, 2H), 7.45-7.42 (m, 3H), 7.34-7.31 (m, 2H), 7.26-7.24 (m, 3H), 4.39 (t, $J$ = 3.6 Hz, 2H), 2.87-2.81 (m, 2H), 1.40-1.34 (m, 2H). $^{13}$C NMR (75 MHz, $CDCl_3$) δ = 144.02, 135.30, 132.21, 129.69, 128.41, 128.09, 127.94, 125.85, 31.15, 12.13. The spectra are in accordance with the literature.[1]

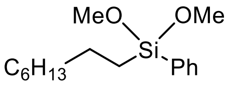


**Dimethoxy(octyl)(phenyl)silane (1g).**

Prepared according to the general procedure, using 0.1% mol of catalyst dissolved in methanol, 1-octene (100 mg, 0.89 mmol) and phenylsilane (96.3 mg, 0.89 mmol). The reaction was stirred for 6 hours at room temperature. $^{1}$H NMR (300 MHz, $CDCl_3$) δ = 7.71-7.68 (m, 2H), 7.45-7.40 (m, 3H), 3.36 (s, 6H), 1.28-0.87 (m, 17H). $^{13}$C NMR (75 MHz, $CDCl_3$) δ = 134.79, 134.31, 130.67, 128.02, 50.85, 33.27, 31.90, 29.25, 29.21, 24.52, 22.68, 14.11, 11.94.

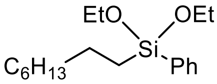


**Diethoxy(octyl)(phenyl)silane (1h).**

Prepared according to the general procedure, using 0.1% mol of catalyst dissolved in ethanol, 1-octene (100 mg, 0.89 mmol) and phenylsilane (96.3 mg, 0.89 mmol). The reaction was stirred for 6 hours at room temperature. $^{1}$H NMR (300 MHz, $CDCl_3$) δ = 7.65-7.60 (m, 2H), 7.43-7.37 (m, 3H), 3.96 (q, 4H), 1.39 (t, 6H), 1.23-0.84 (m, 17H). $^{13}$C NMR (75 MHz, $CDCl_3$) δ = 134.75, 134.27, 130.65, 128.01, 57.85, 33.27, 31.90, 29.25, 29.21, 24.52, 22.68, 18.21, 14.11, 11.94.

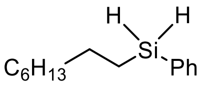


**Octyl(phenyl)silane (1i).**

Prepared according to the general procedure, using 0.1% mol of catalyst dissolved in methanol/water (1:1) or ethanol/water (1:1), 1-octene (100 mg, 0.89 mmol) and phenylsilane (96.3 mg, 0.89 mmol). The reaction was stirred for 6 hours at room temperature. $^1$H NMR (300 MHz, $CDCl_3$) δ = 7.62-7.60 (m, 2H), 7.41-7.39 (m, 3H), 4.33 (t, J = 3.6 Hz, 2H), 1.42-0.90 (m, 17H). $^{13}$C NMR (75 MHz, $CDCl_3$) δ = 135.62, 135.21, 129.45, 127.94, 32.81, 31.87, 29.21, 29.17, 25.06, 22.64, 14.07, 10.00. The spectra are in accordance with the literature.[1]

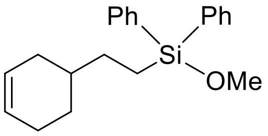


**(2-(cyclohex-3-en-1-yl)ethyl)(methoxy)diphenylsilane (2a).**

Prepared according to the general procedure, using 0.1% mol of catalyst dissolved in methanol, 4-vinylcyclohexene (96.3 mg, 0.89 mmol) and diphenylsilane (164 mg, 0.89 mmol). The reaction was stirred for 24 hours at room temperature. $^1$H NMR (300 MHz, $CDCl_3$): δ = 7.56-7.54 (m, 4H), 7.39-7.34 (m, 6H), 5.53 (s, 2H), 3.65 (s, 3H), 1.71-1.05 (m, 11H). $^{13}$C NMR (75 MHz, $CDCl_3$) δ = 135.29, 134.71, 129.67, 128.08, 127.15, 126.58, 50.92, 36.43, 31.64, 31.18, 28.51, 25.45, 9.33.

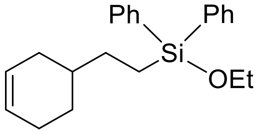


**(2-(cyclohex-3-en-1-yl)ethyl)(ethoxy)diphenylsilane (2b).**

Prepared according to the general procedure, using 0.1% mol of catalyst dissolved in ethanol, 4-vinylcyclohexene (96.3 mg, 0.89 mmol) and diphenylsilane (164 mg, 0.89 mmol). The reaction was stirred for 24 hours at room temperature. $^1$H NMR (300 MHz, $CDCl_3$): δ = 7.71-7.64 (m, 4H), 7.45-7.40 (m, 6H), 5.68 (s, 2H), 3.99 (q, 2H), 2.14-1.14 (m, 14H). $^{13}$C NMR (75 MHz, $CDCl_3$) δ = 135.32, 134.75, 129.71, 128.19, 127.23, 126.80, 58.06, 36.54, 31.71, 31.27, 28.59, 25.46, 18.34, 9.35.

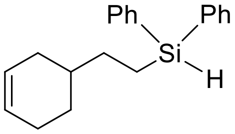


**(2-(cyclohex-3-en-1-yl)ethyl)diphenylsilane (2c).**

Prepared according the to general procedure, using 0.1% mol of catalyst in dissolved methanol/water (1:1) or ethanol/water (1:1), 4-vinylcyclohexene (96.3 mg, 0.89 mmol) and diphenylsilane (164 mg, 0.89 mmol). The reaction was stirred for 24 hours at room temperature. $^1$H NMR (300 MHz, $CDCl_3$): δ = 7.49-7.46 (m, 4H), 7.30-7.28 (m, 6H), 5.57 (s, 2H), 4.77 (t, J = 3.7 Hz, 1H), 1.71-1.09 (m, 11H). $^{13}$C NMR (75 MHz, $CDCl_3$) δ = 135.15, 134.63, 129.52, 127.99, 127.08, 126.58, 36.36, 31.57, 31.10, 28.44, 25.31, 9.26. The spectra are in accordance with the literature.[1]

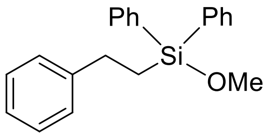


**Methoxy(phenethyl)diphenylsilane (2d).**

Prepared according to the general procedure, using 0.1% mol of catalyst dissolved in methanol, styrene (92.7 mg, 0.89 mmol) and diphenylsilane (164 mg, 0.89 mmol). The reaction was stirred for 24 hours at room temperature. $^1$H NMR (300 MHz, $CDCl_3$): δ = 7.69-7.66 (m, 4H), 7.49-7.45 (m, 6H), 7.35-7.33 (m, 2H), 7.29-7.25 (m, 3H), 3.54 (s, 3H), 2.89-2.84 (m, 2H), 1.65-1.58 (m, 2H). $^{13}$C NMR (75 MHz, $CDCl_3$) δ = 144.40, 135.23, 134.19, 129.74, 128.42, 128.15, 127.94, 125.80, 50.11, 30.53, 14.39. The spectra are in accordance with the literature. [18]

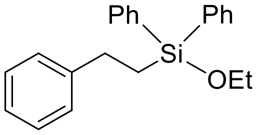


**Ethoxy(phenethyl)diphenylsilane (2e).**

Prepared according to the general procedure, using 0.1% mol of catalyst dissolved in ethanol, styrene (92.7 mg, 0.89 mmol) and diphenylsilane (164 mg, 0.89 mmol). The reaction was stirred for 24 hours at room temperature. $^1$H NMR (300 MHz, $CDCl_3$): δ = 7.72-7.69 (m, 4H), 7.52-7.48 (m, 6H), 7.38-7.35 (m, 2H), 7.31-7.28 (m, 3H), 3.98 (q, 2H), 2.92-2.86 (m, 2H), 1.67-1.60 (m, 2H), 1.38 (t, 3H). $^{13}$C NMR (75 MHz, $CDCl_3$) δ = 144.38, 135.20, 134.16, 129.70, 128.39, 128.10, 127.90, 125.77, 57.97, 30.49, 18.22, 14.34.

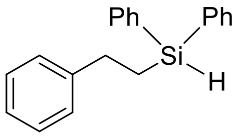


**Phenethyldiphenylsilane (2f).**

Prepared according to the general procedure, using 0.1% mol of catalyst dissolved in methanol/water (1:1) or ethanol/water (1:1), styrene (92.7 mg, 0.89 mmol) and diphenylsilane (164 mg, 0.89 mmol). The reaction was stirred for 24 hours at room temperature. $^1$H NMR (300 MHz, $CDCl_3$): δ = 7.51-7.47 (m, 4H), 7.32-7.27 (m, 6H), 7.20-7.15 (m, 2H), 7.10-7.07 (m, 3H), 4.82 (t, $J$ = 3.6 Hz, 1H), 2.71-2.65 (m, 2H), 1.46-1.39 (m, 2H). $^{13}$C NMR (75 MHz, $CDCl_3$) δ = 144.40, 135.21, 134.17, 129.69, 128.39, 128.10, 127,90, 125.77, 30.49, 14.32. The spectra are in accordance with the literature.[1]

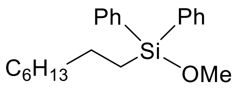


**Methoxy(octyl)diphenylsilane (2g).**

Prepared according to the general procedure, using 0.1% mol of catalyst dissolved in methanol, 1-octene (100 mg, 0.89 mmol) and diphenylsilane (164 mg, 0.89 mmol). The reaction was stirred for 24 hours at room temperature. $^1$H NMR (400 MHz, $CDCl_3$): δ = 7.74-7.71 (m, 4H), 7.47-7.41 (m, 6H), 3.69 (s, 3H,), 1.35-0.91 (m, 17H). $^{13}$C NMR (75 MHz, $CDCl_3$) δ = 135.19, 134.89, 129.51, 127.98, 50.98, 33.22, 31.93, 29.32, 29.25, 24.44, 22.70, 14.15, 12.19. The spectra are in accordance with the literature.[18]

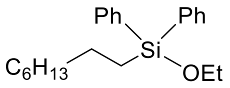


**Ethoxy(octyl)diphenylsilane (2h).**

Prepared according to the general procedure, using 0.1% mol of catalyst dissolved in ethanol, 1-octene (100 mg, 0.89 mmol) and diphenylsilane (164 mg, 0.89 mmol). The reaction was stirred for 24 hours at room temperature. $^{1}$H NMR (400 MHz, $CDCl_3$): δ = 7.78-7.63 (m, 4H), 7.49- 7.41 (m, 6H), 3.97 (q, 2H), 1.56-0.94 (m, 20H). $^{13}$C NMR (75 MHz, $CDCl_3$) δ = 135.21, 134.95, 129.53, 127.87, 58.99, 33.25, 31.95, 29.27, 29.25, 24.47, 22.73, 18.39, 14.17, 12.22. The spectra are in accordance with the literature.[18]

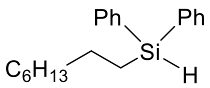


**Octyl(diphenyl)silane (2i).**

Prepared according to the general procedure, using 0.1% mol of catalyst dissolved in ethanol, 1-octene (100 mg, 0.89 mmol) and diphenylsilane (164 mg, 0.89 mmol). The reaction was stirred for 24 hours at room temperature. $^{1}$H NMR (400 MHz, $CDCl_3$): δ = 7.49-7.46 (m, 4H), 7.30-7.27 (m, 6H), 4.77 (t, J = 3.7 Hz, 1H,), 1.38-0.77 (m, 17H). $^{13}$C NMR (75 MHz, $CDCl_3$) δ = 135.15, 134.77, 129.46, 127.95, 33.17, 31.89, 29.72, 29.20, 24.40, 22.66, 14.09, 12.16. The spectra is in accordance with the literature.[1]

## III. In situ, kinetic and mechanistic studies.

### A. Kinetic studies

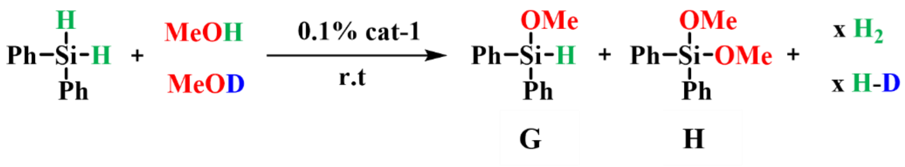


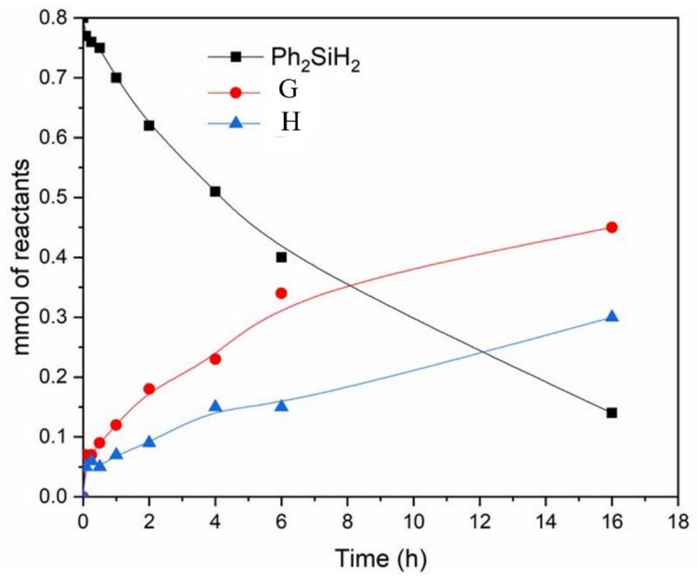


**Figure S3.** Alkoxysilylation reaction of diphenylsilane with methanol at 25ºC catalyzed by **cat-1**.

## B. Isotopic effect: NMR detection of $H_2$/HD

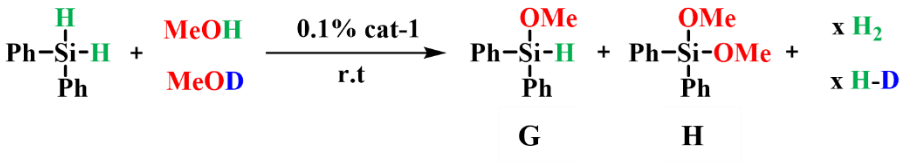


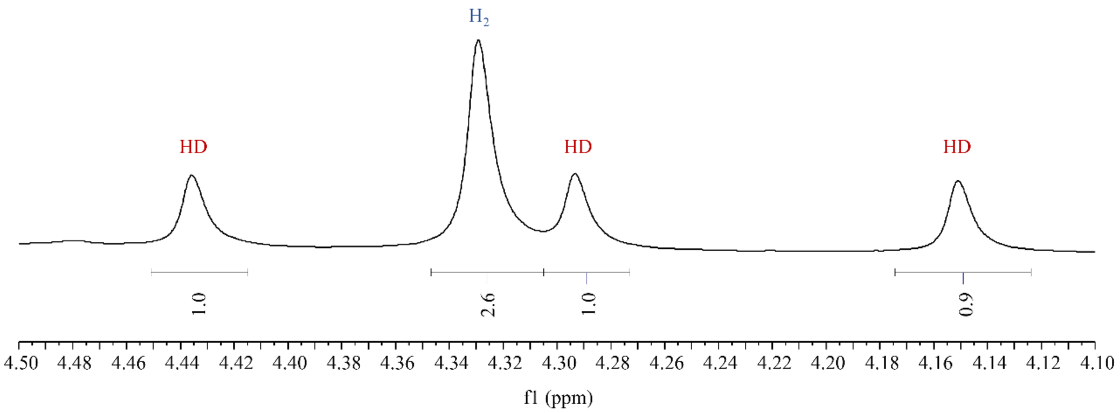


**Figure S4.** $^{1}$H-NMR spectrum obtained for the reaction of $Ph_2SiH_2$ and MeOD (6% of $CD_3OH$) catalyzed by **cat-1**.

## C. EPR Spectroscopy

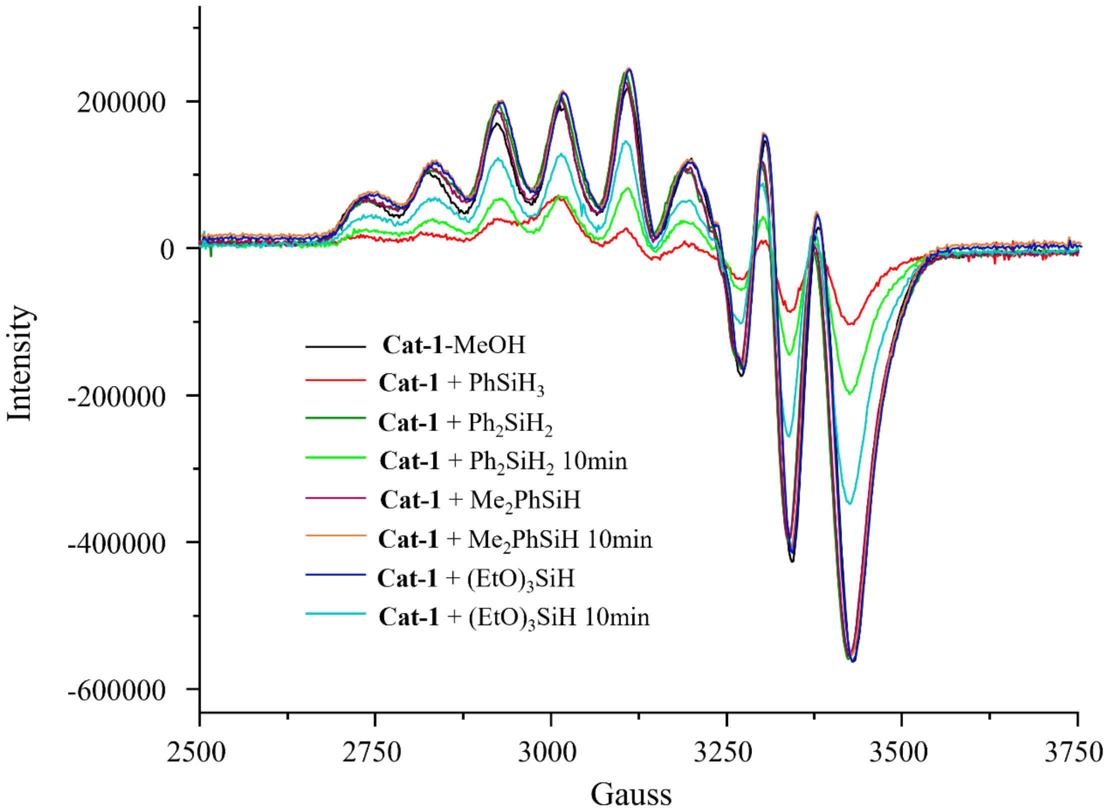


**Figure S5.** EPR spectra of **cat-1** in methanol and after the addition of different silane molecules and after 10 minutes of reaction.

## D. RMN Spectroscopy

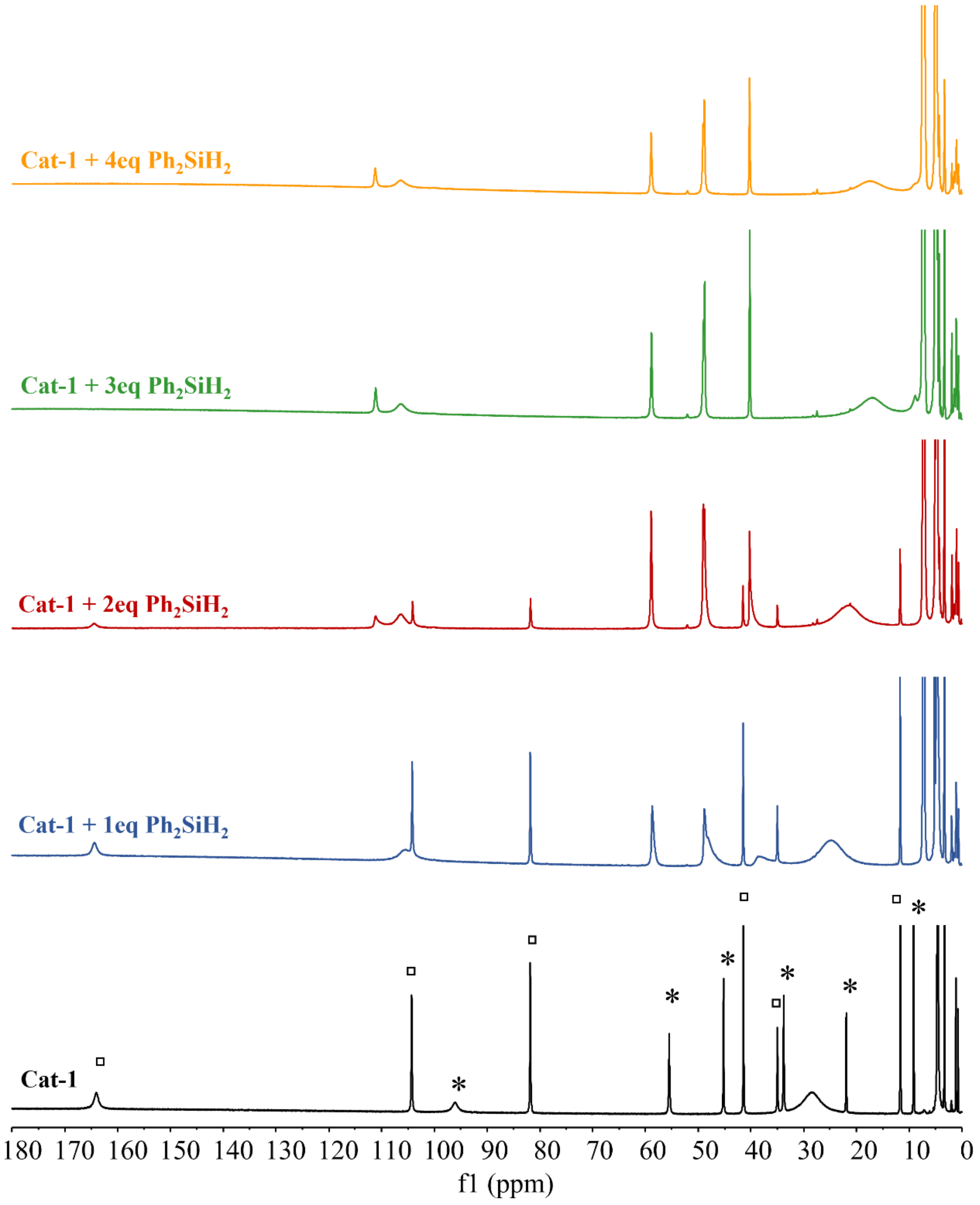


**Figure S6.** $^{1}$H-NMR of **cat-1** in $CD_3OD$ (black) where (*) represents the homoleptic complex with two tpy ligands [Co(tpy)$_2$] and (□) represents [Co(tpy)($H_2O$)OAc]$^+$ and after the addition of one (blue), two (red), three (green) and four (orange) equivalents of $Ph_2SiH_2$.

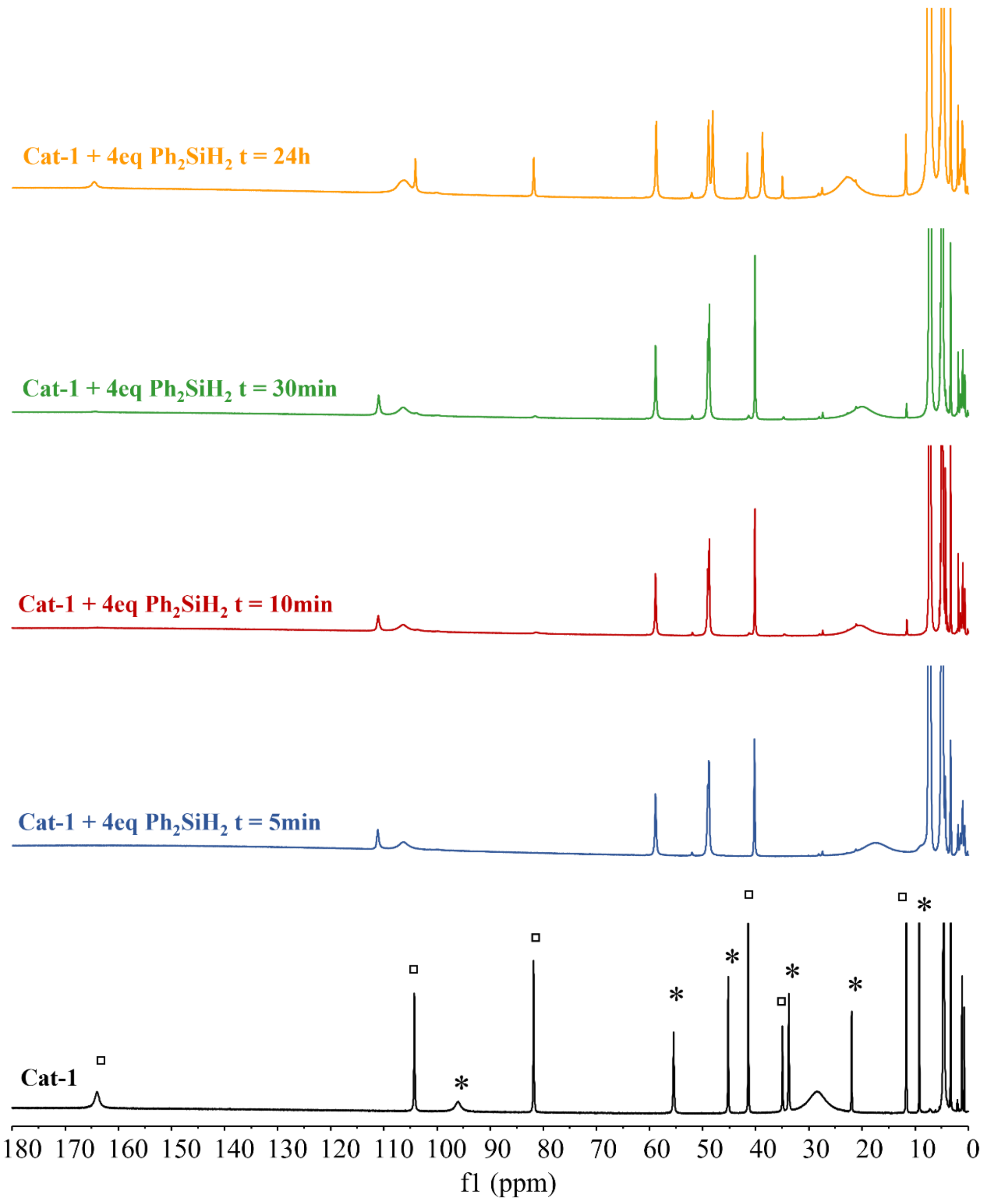


**Figure S7.** $^{1}$H-NMR of **cat-1** in $CD_3OD$ (black) where (*) represents the homoleptic complex with two tpy ligands $[Co(tpy)_2]$ and (□ ) represents $[Co(tpy)(H_2O)OAc]^+$ and after the addition of four equivalents of $Ph_2SiH_2$ at 5 minutes (blue), 10 minutes (red), 30 minutes (green) and 24 hours (orange).

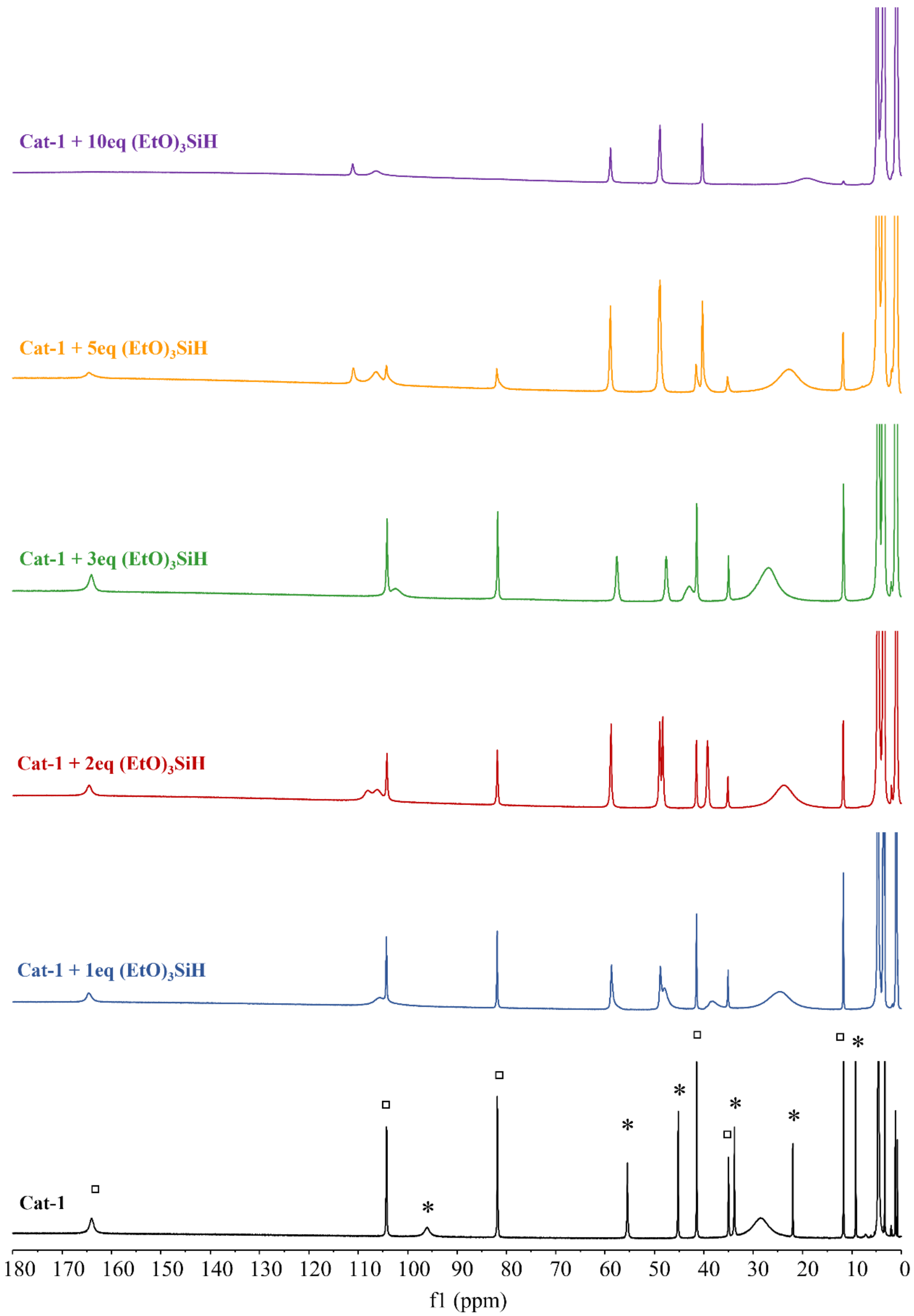


**Figure S8.** $^{1}$H-NMR of **cat-1** in $CD_3OD$ (black) where (*) represents the homoleptic complex with two tpy ligands $[Co(tpy)_2]$ and (□) represents $[Co(tpy)(H_2O)OAc]^+$ and after the addition of one (blue), two (red), three (green), five (orange) and ten (purple) equivalents of $EtO_3SiH$.

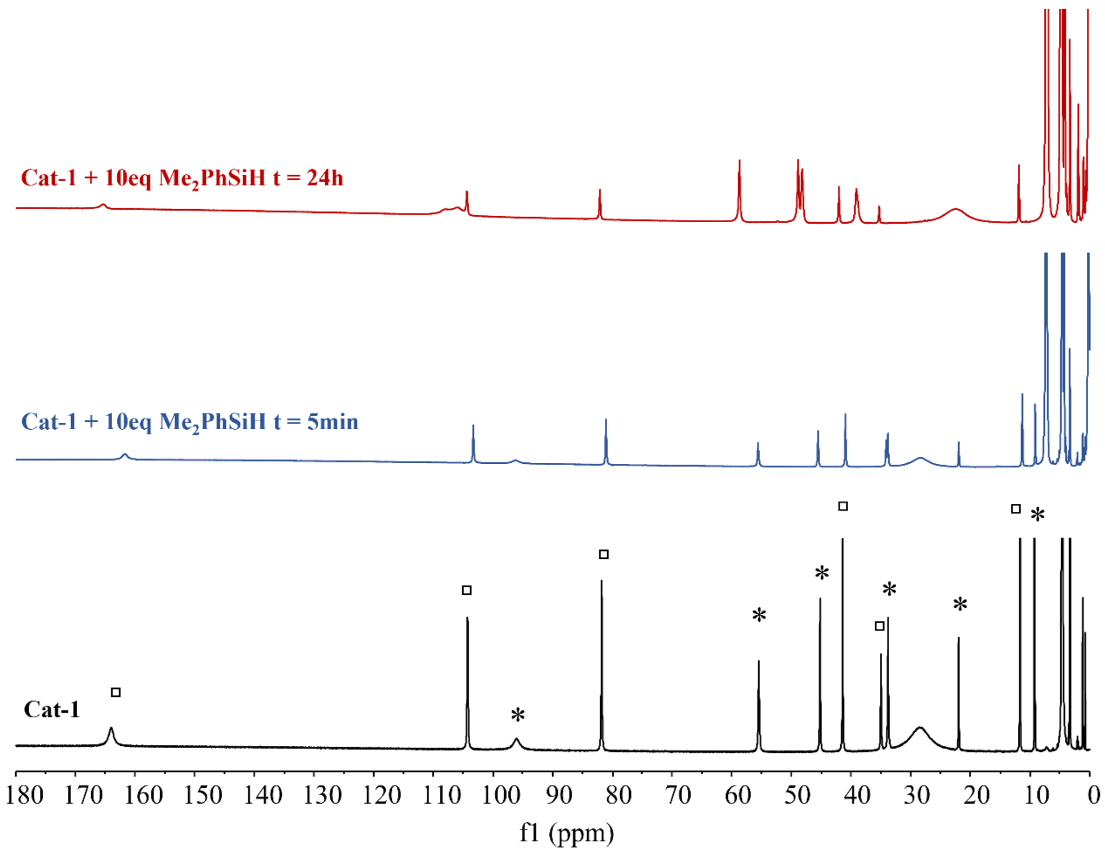


**Figure S9.** $^{1}$H-NMR of **cat-1** in $CD_3OD$ (black) where (*) represents the homoleptic complex with two tpy ligands $[Co(tpy)_2]$ and (□) represents $[Co(tpy)(H_2O)OAc]^+$ and after the addition of ten equivalents of $Me_2PhSiH$ at 5 minutes (blue) and 24 hours (red).

## E. ESI-MS

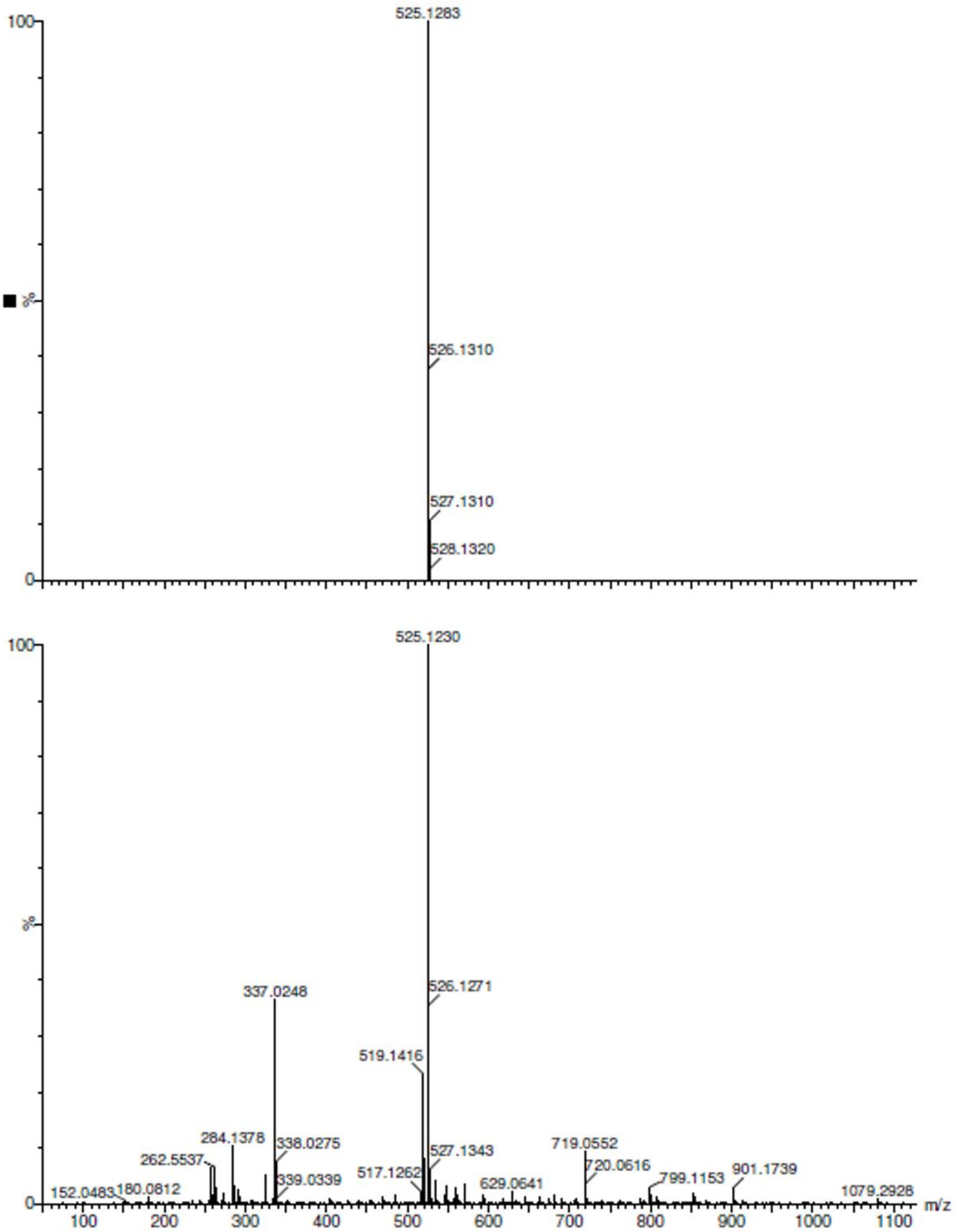


**Figure S10.** Theoric ESI spectrum for the formula $C_{28}H_{28}N_3O_2SiCo$ (above) and experimental ESI spectrum of **cat-1** in methanol solution after addition of 10 equivalents of $Ph_2SiH_2$ which correspond with the formula $C_{28}H_{28}N_3O_2SiCo$ (below).

## F. Raman Spectroscopy

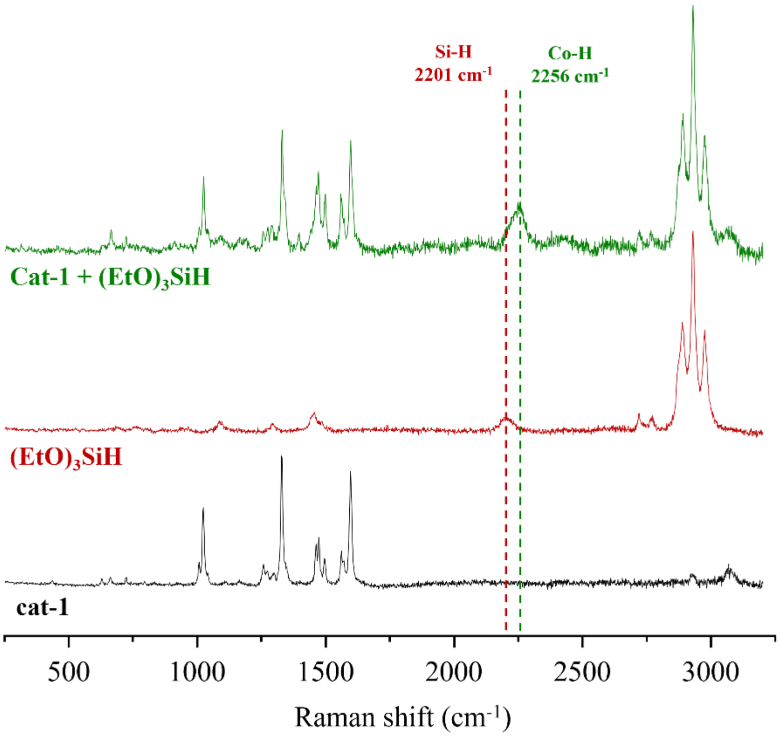


**Figure S11.** In situ Raman experiment. **Cat-1** in methanol (black), $EtO_3SiH$ (red) and the reaction mixture between **cat-1** and $EtO_3SiH$ (green).

To explore the shift of 55 $cm^{-1}$ of the Si-H band at 2256 $cm^{-1}$ (Figure S11), we calculated different coordination modes between a model $SiH(OMe)_3$ and the complex $[Co(tpy)(OMe)]^+$. The most relevant structures, Gibbs energies, and Si-H vibrations with respect to the bare silane ($\Delta\nu$) are shown in Scheme S1. It is clear that a σ-bond complex (**III**) can be ruled out due to the high energy as well as the decreasing Si-H stretching of -77 $cm^{-1}$. Instead, we propose a monodentate coordination of the silane to the Co via OMe (**I**), which presents a slight shift of +26 $cm^{-1}$. The bidentate version (**II**) could also be possible, but it is higher in energy.

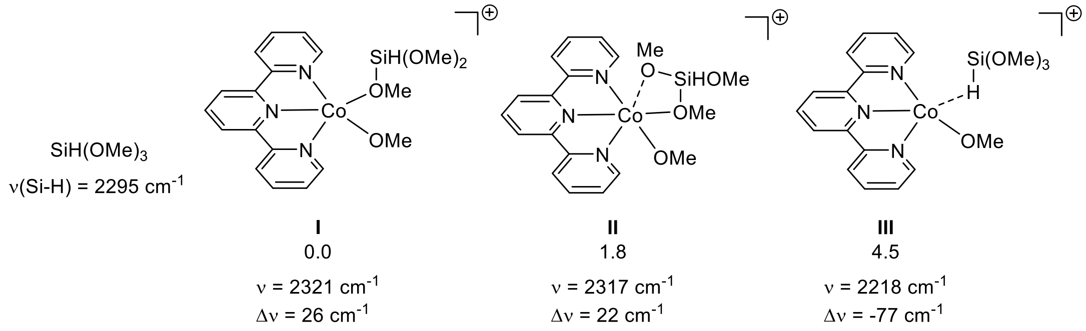


**Scheme S1.** Computed coordination modes between silane and Co complex.

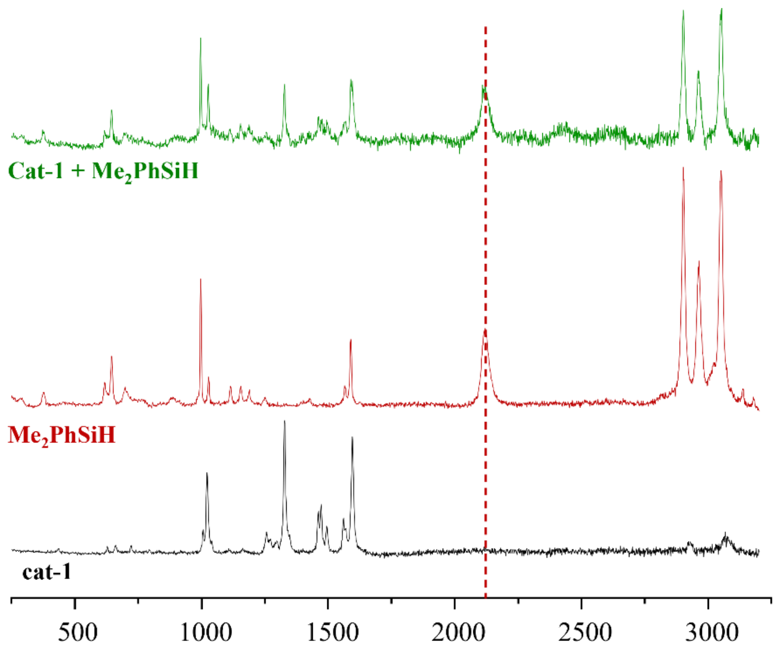


**Figure S12.** In situ Raman experiment. **Cat-1** in methanol (black), $Me_2PhSiH$ (red) and the reaction mixture between **cat-1** and $Me_2PhSiH$ (green).

## IV. NMR Spectra of Silane products.

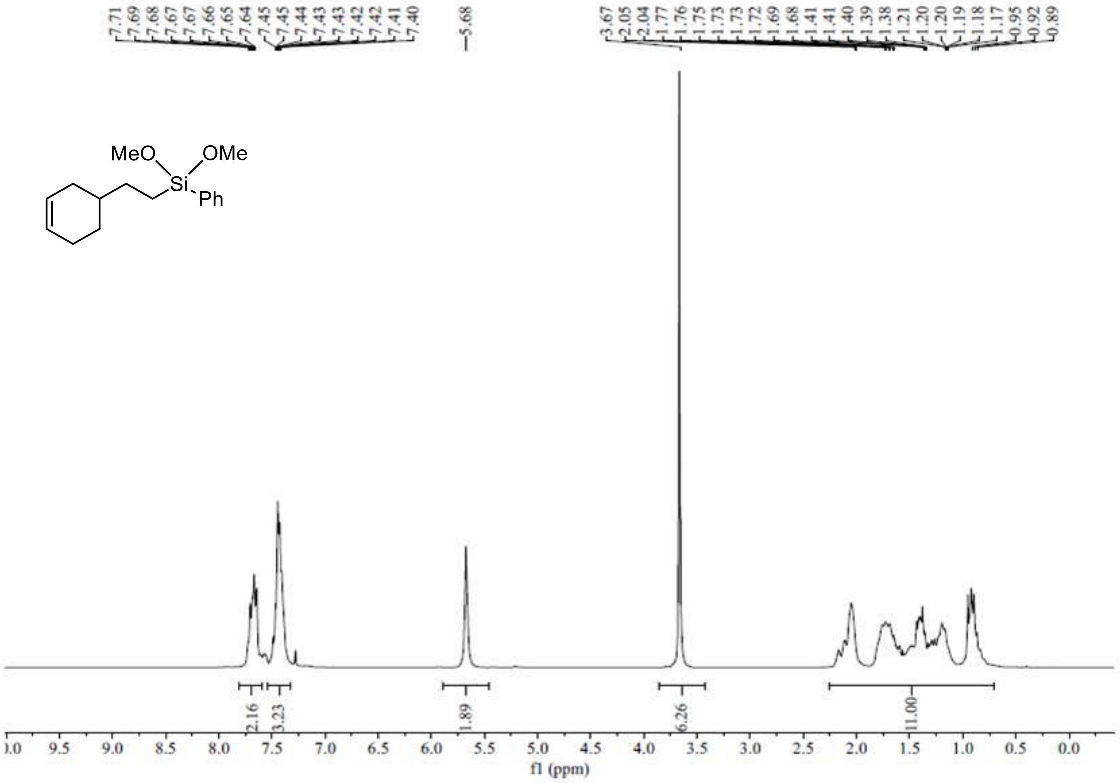


**Figure S13.** $^{1}$H-NMR spectrum of (2-(cyclohex-3-en-1-yl)ethyl)dimethoxy(phenyl)silane.

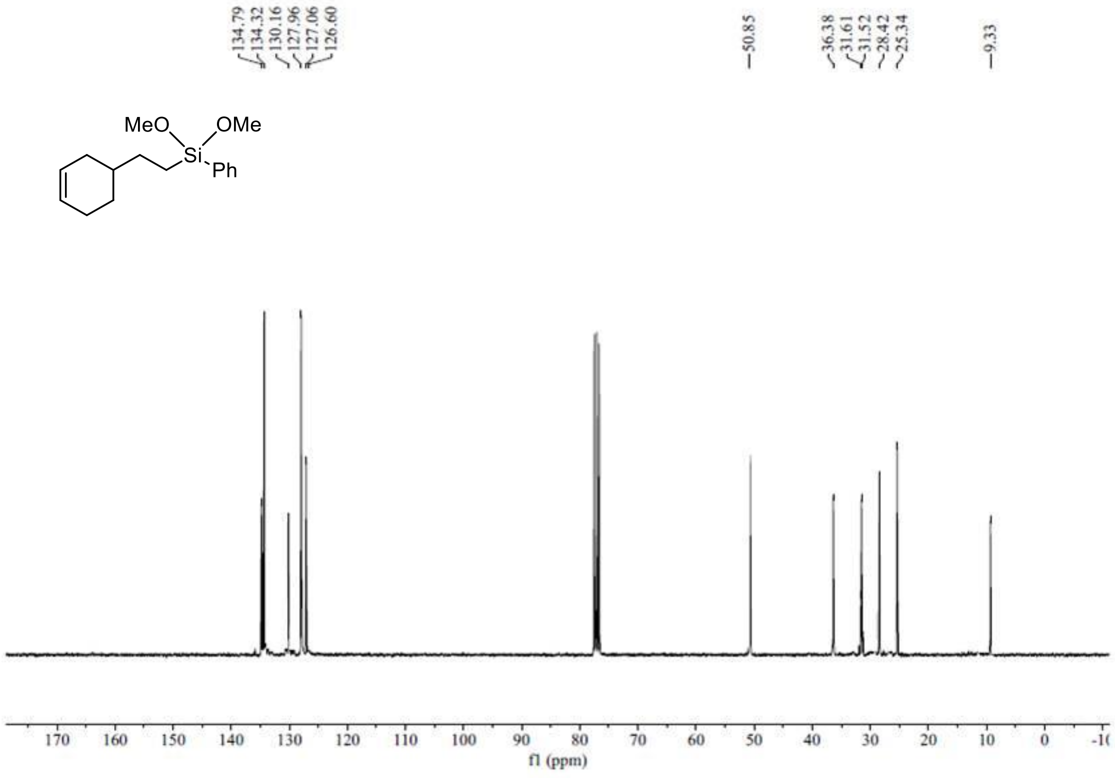


**Figure S14.** $^{13}$C-NMR spectrum of (2-(cyclohex-3-en-1-yl)ethyl)dimethoxy(phenyl)silane.

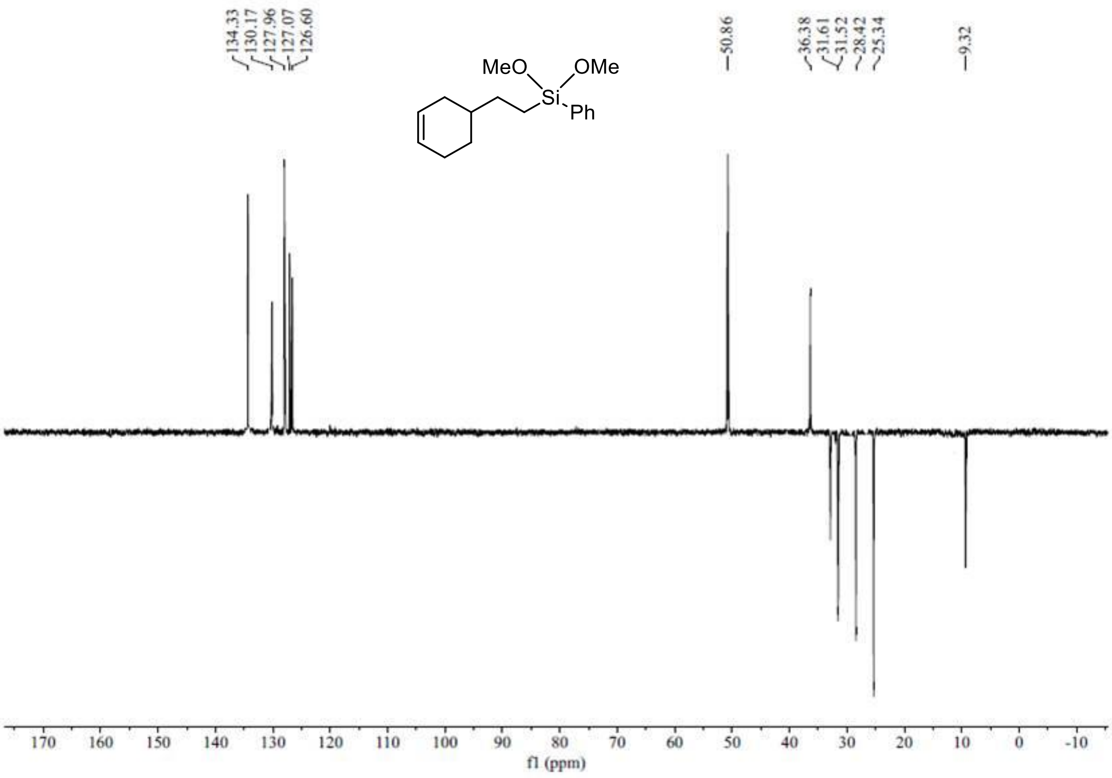


**Figure S15.** DEPT spectrum of (2-(cyclohex-3-en-1-yl)ethyl)dimethoxy(phenyl)silane.

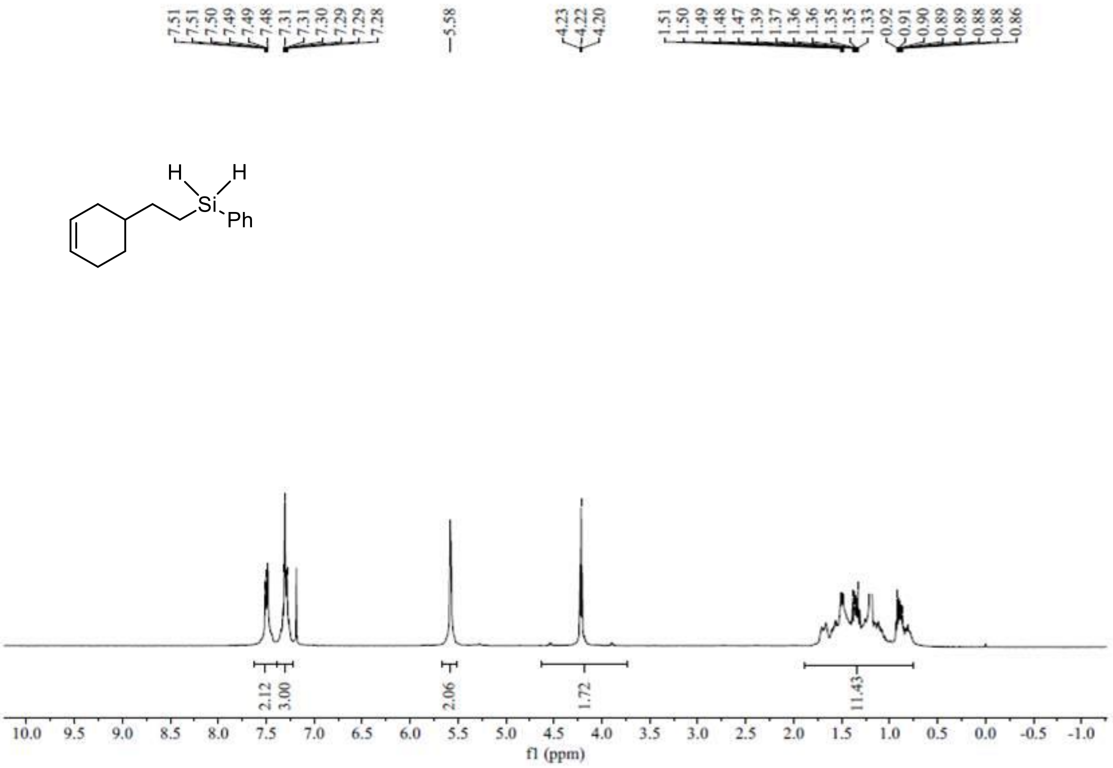


**Figure S16.** $^{1}$H-NMR spectrum of (2-(cyclohex-3-en-1-yl)ethyl)(phenyl)silane.

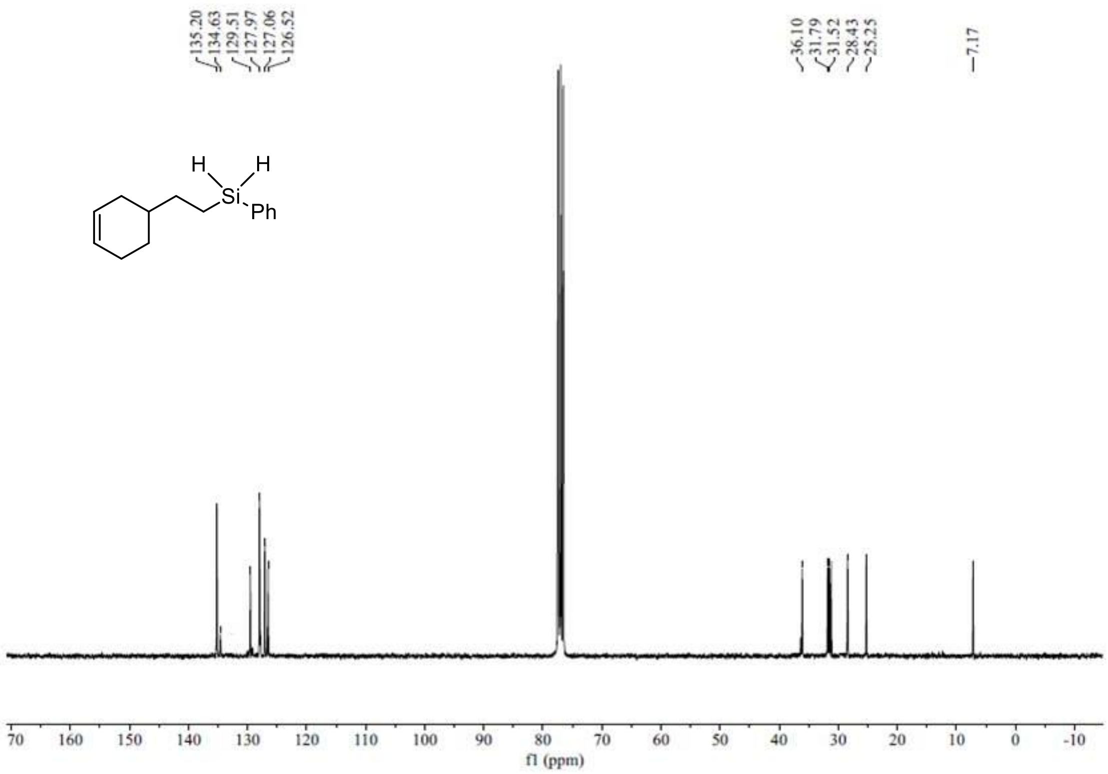


**Figure S17.** $^{13}$C-NMR spectrum of (2-(cyclohex-3-en-1-yl)ethyl)(phenyl)silane.

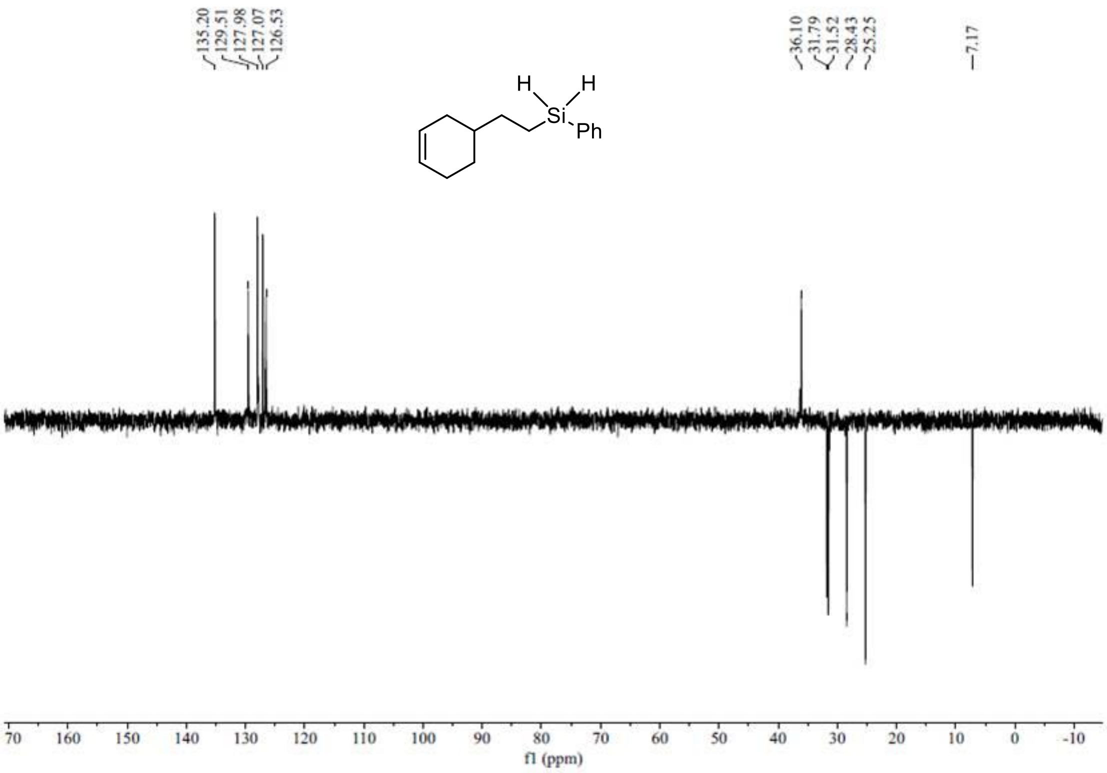


**Figure S18.** DEPT-NMR spectrum of (2-(cyclohex-3-en-1-yl)ethyl)(phenyl)silane.

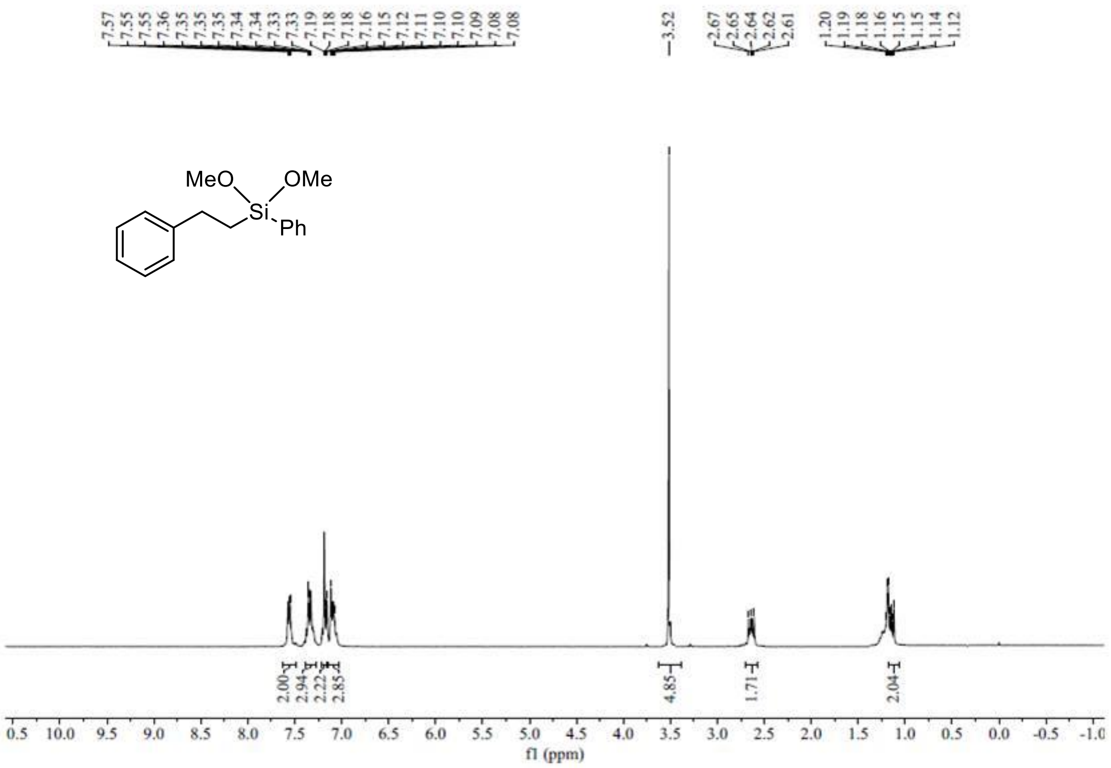


**Figure S19.** $^{1}H$-NMR spectrum of dimethoxy(phenethyl)(phenyl)silane

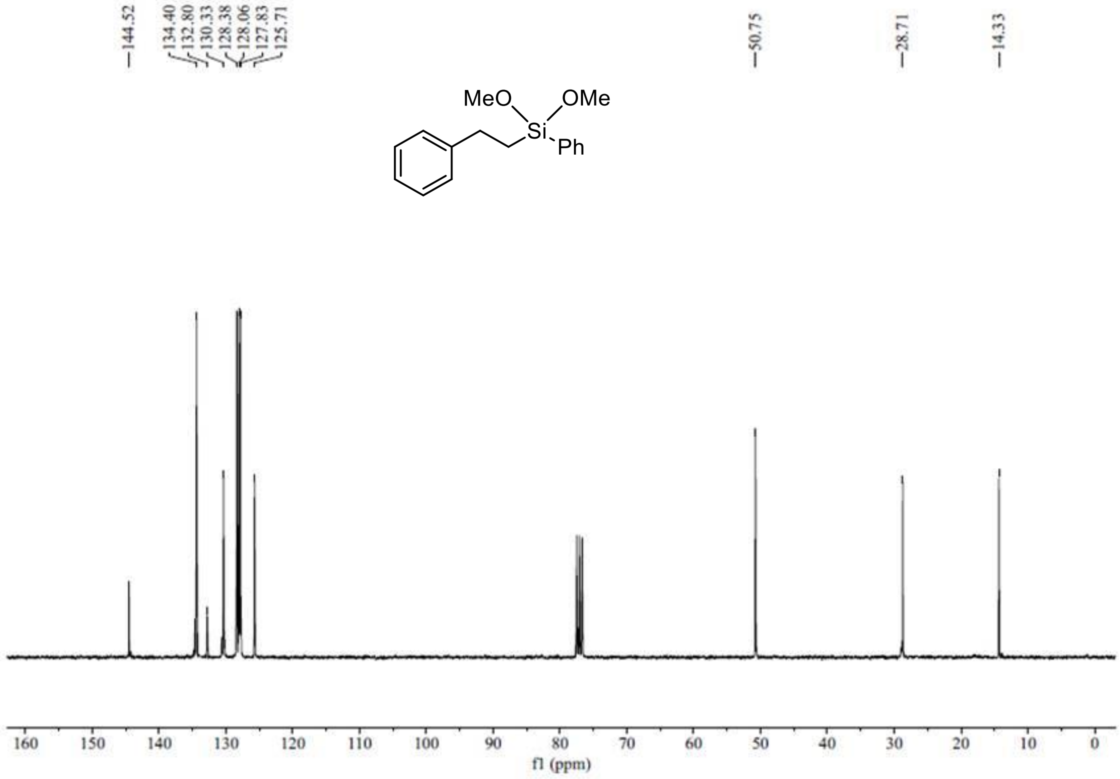


**Figure S20.** $^{13}C$-NMR spectrum of dimethoxy(phenethyl)(phenyl)silane

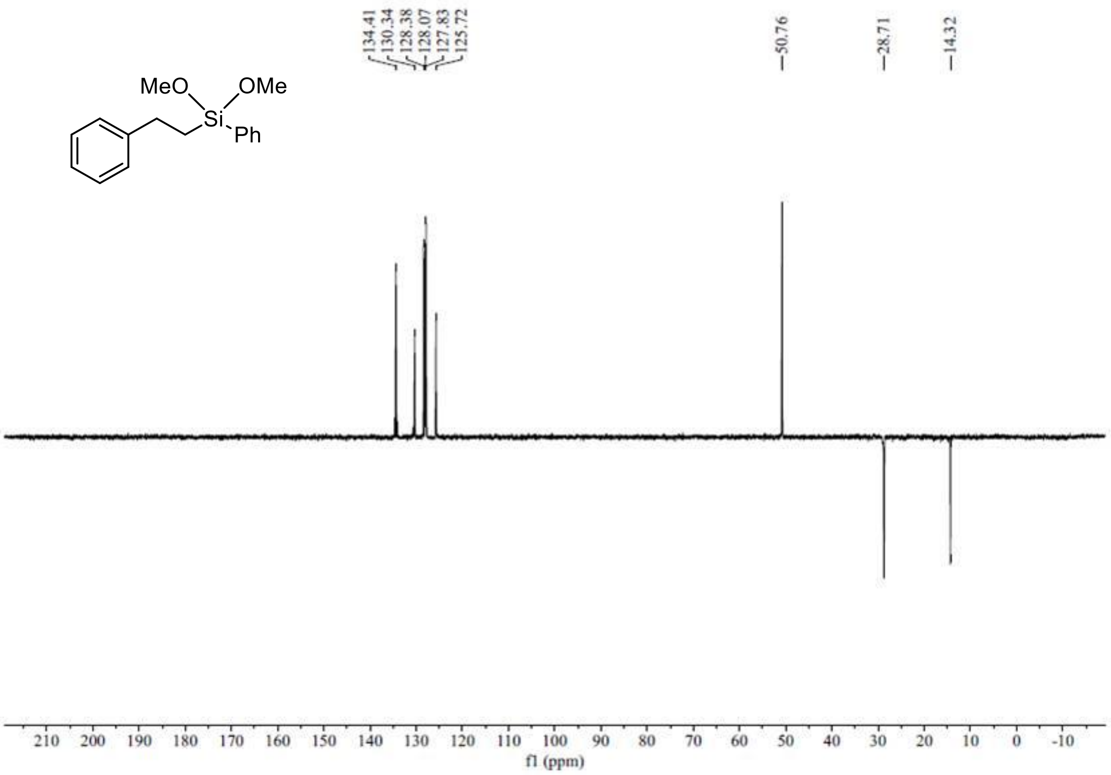


**Figure S21.** DEPT-NMR spectrum of dimethoxy(phenethyl)(phenyl)silane

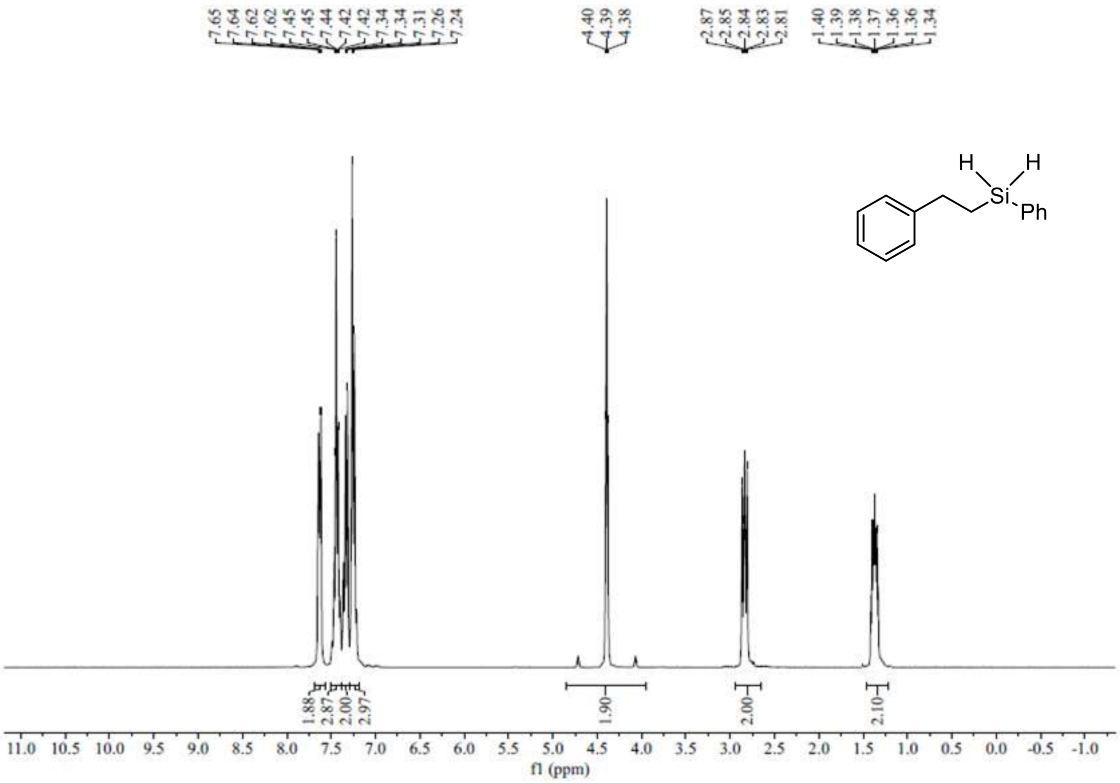


**Figure S22.** $^{1}$H-NMR spectrum of phenethyl(phenyl)silane.

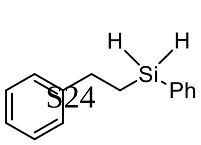

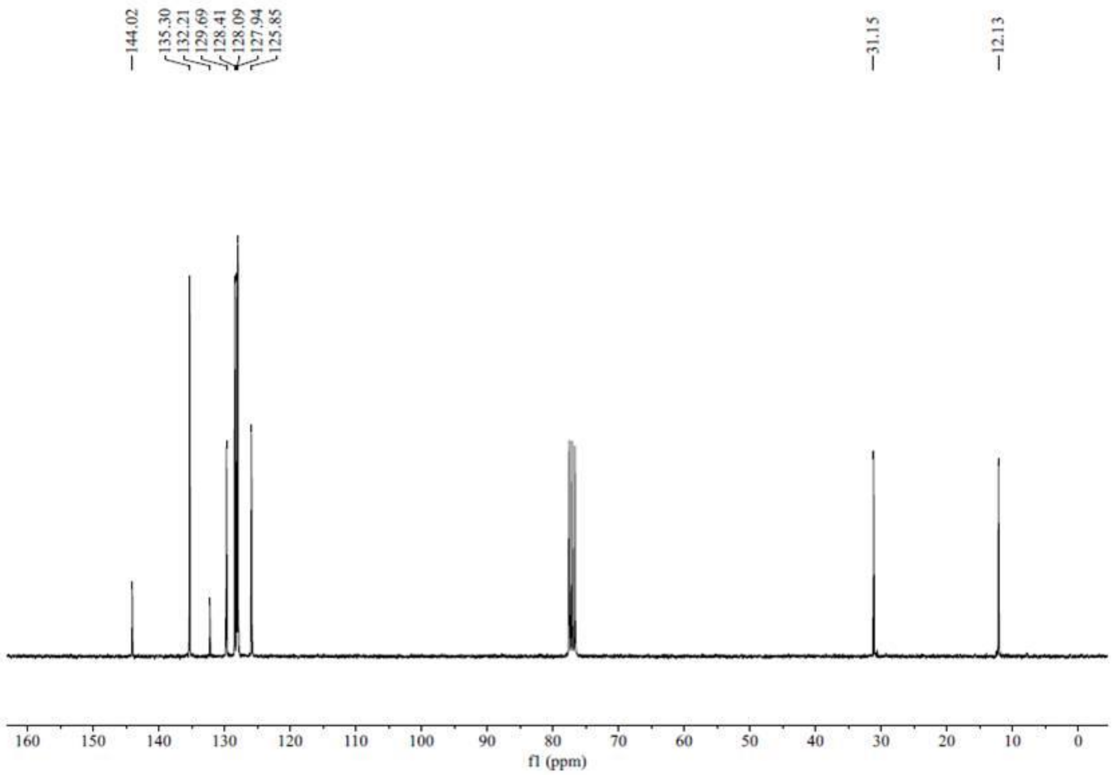


**Figure S23.** $^{13}$C-NMR spectrum of phenethyl(phenyl)silane.

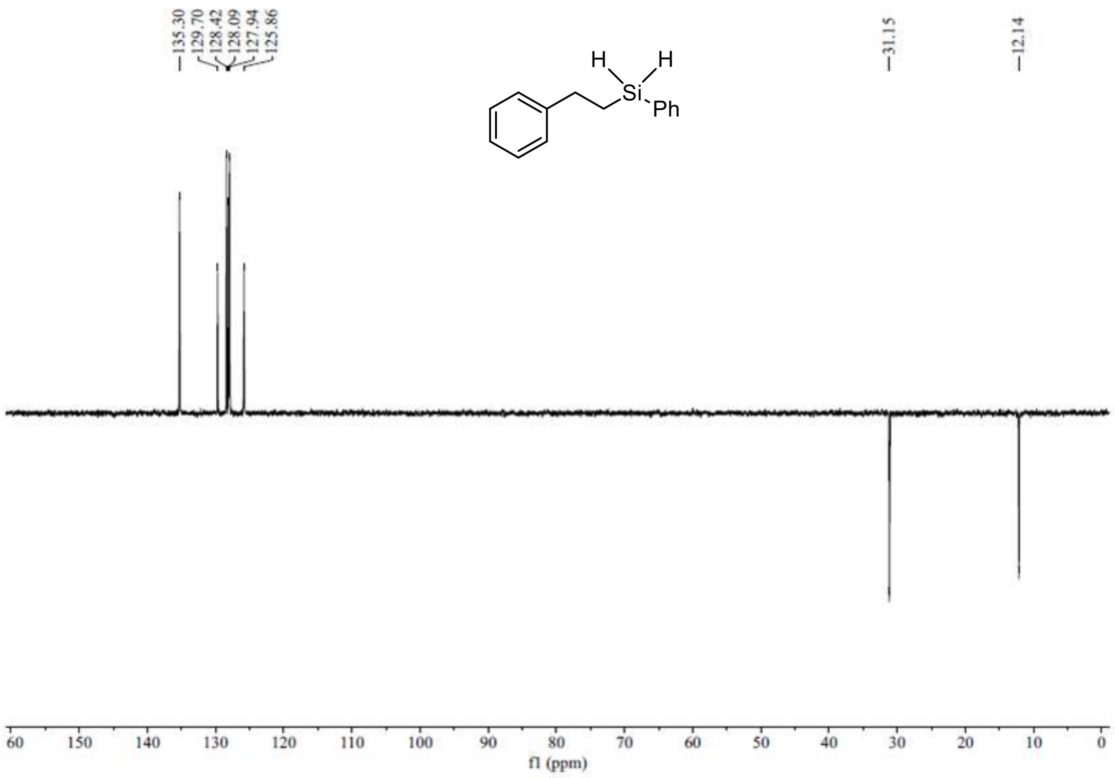


**Figure S24.** DEPT-NMR spectrum of phenethyl(phenyl)silane.

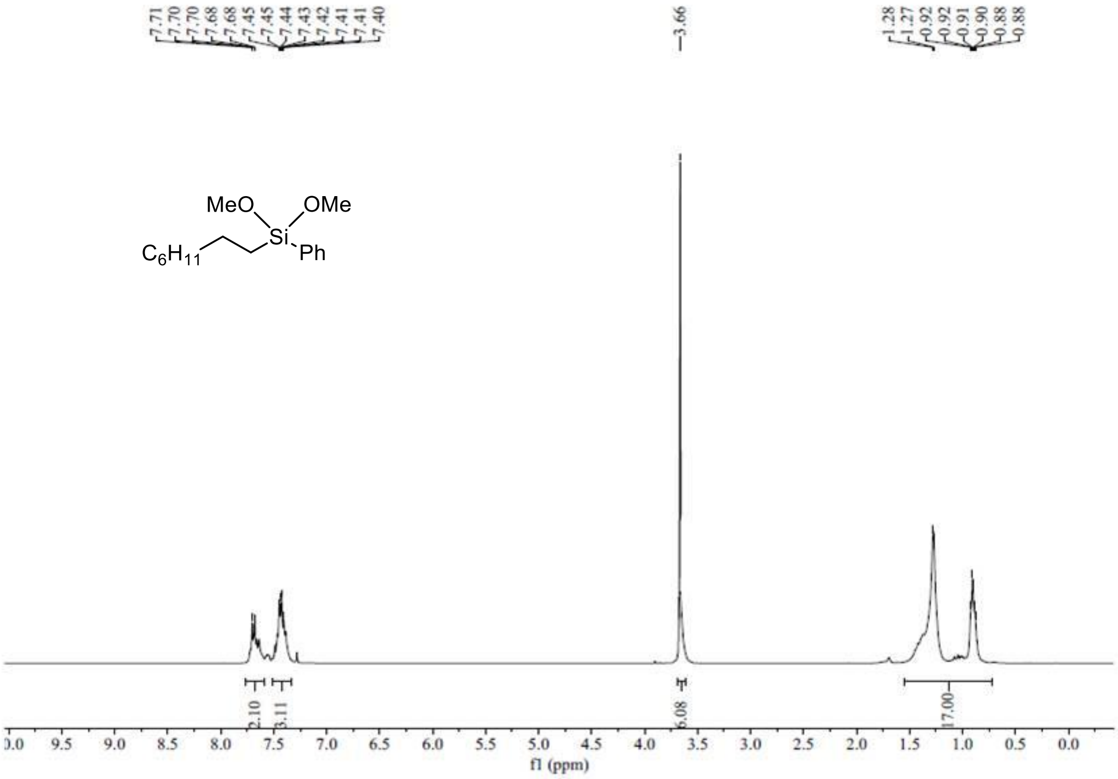


**Figure S25.** $^{1}$H-NMR spectrum of dimethoxy(octyl)(phenyl)silane.

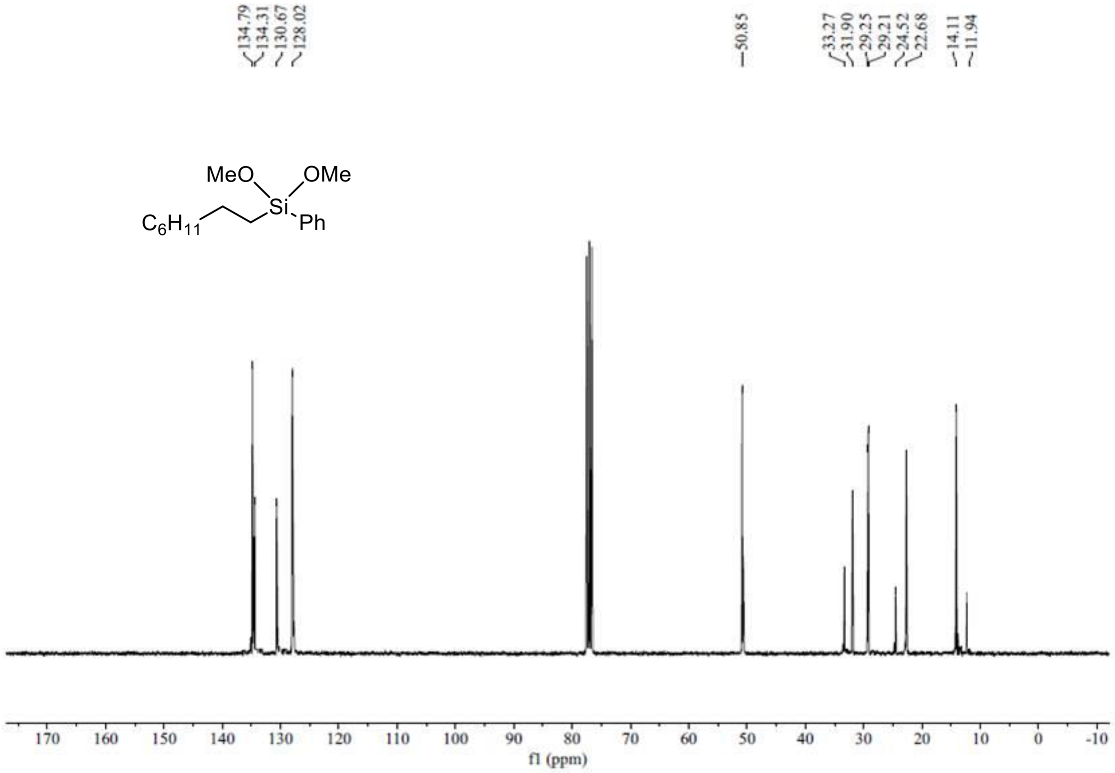


**Figure S26.** $^{13}$C-NMR spectrum of dimethoxy(octyl)(phenyl)silane.

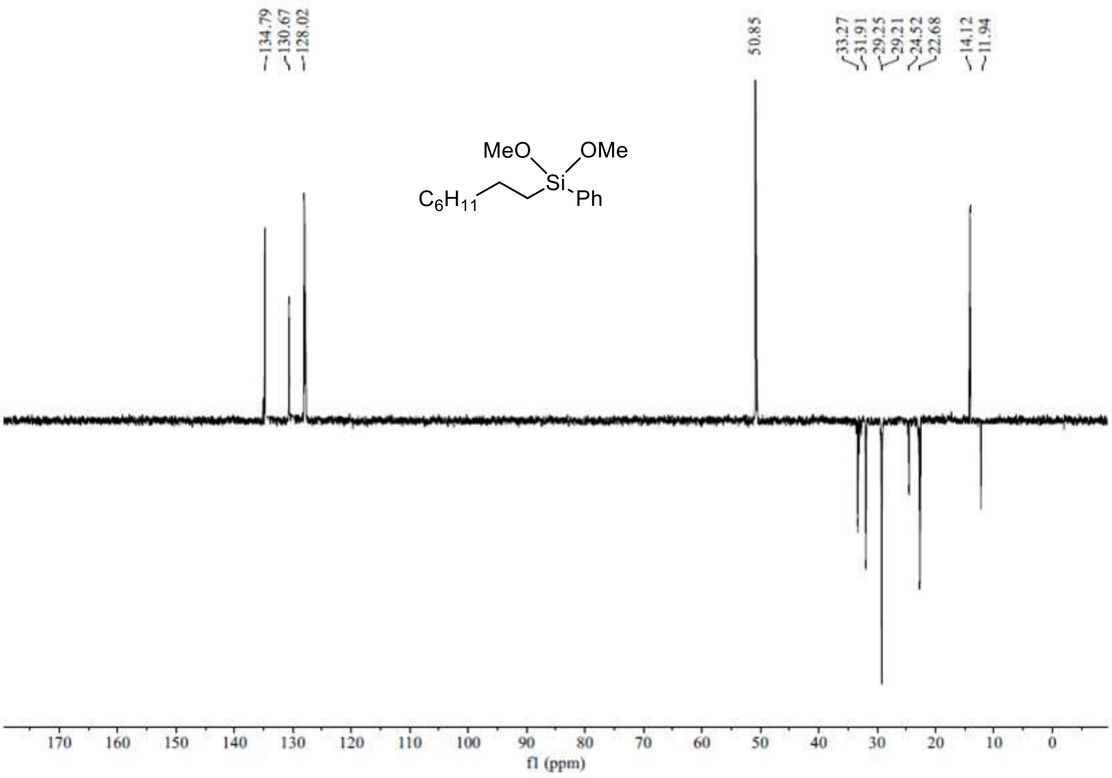


**Figure S27.** DEPT-NMR spectrum of dimethoxy(octyl)(phenyl)silane.

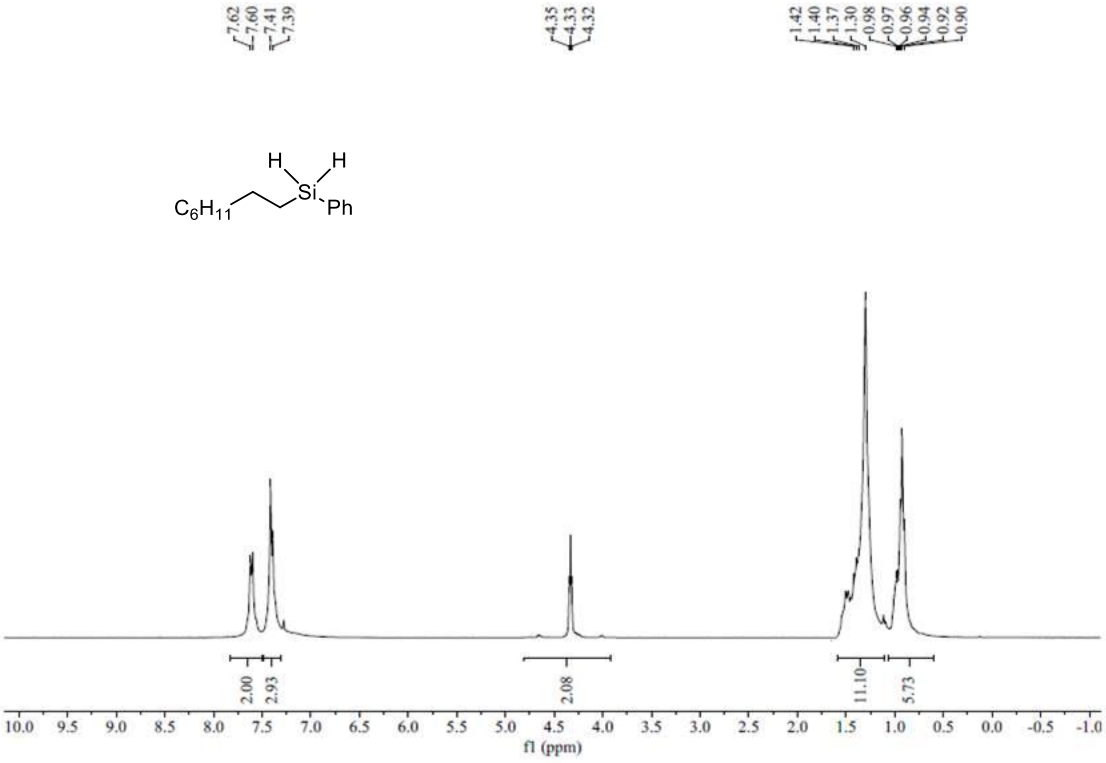


**Figure S28.** $^1$H-NMR spectrum of octyl(phenyl)silane.

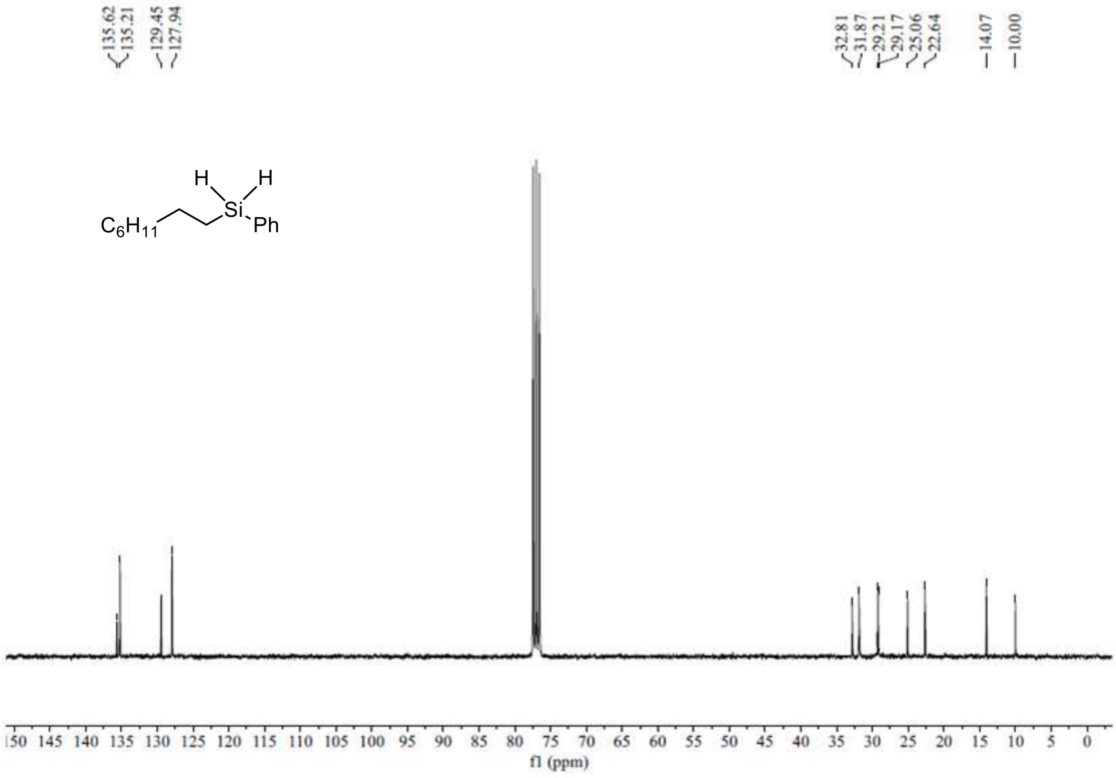


**Figure S29.** $^{13}$C-NMR spectrum of octyl(phenyl)silane.

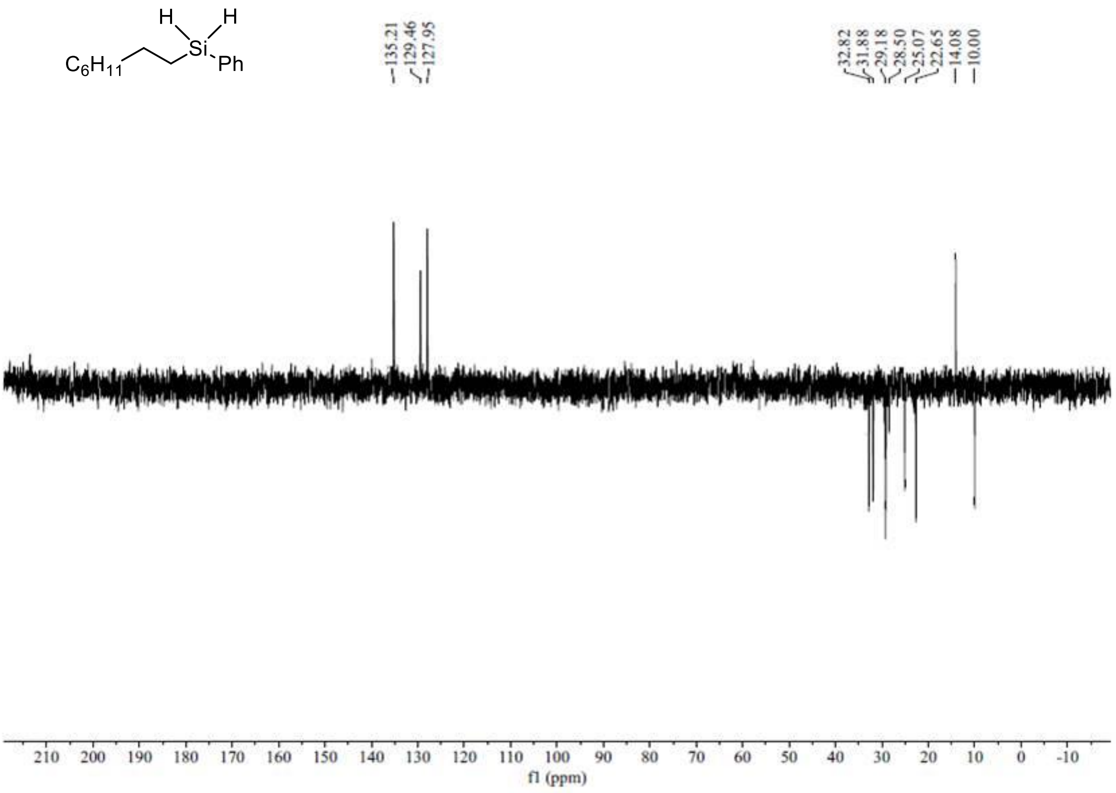


**Figure S30.** DEPT-NMR spectrum of octyl(phenyl)silane.

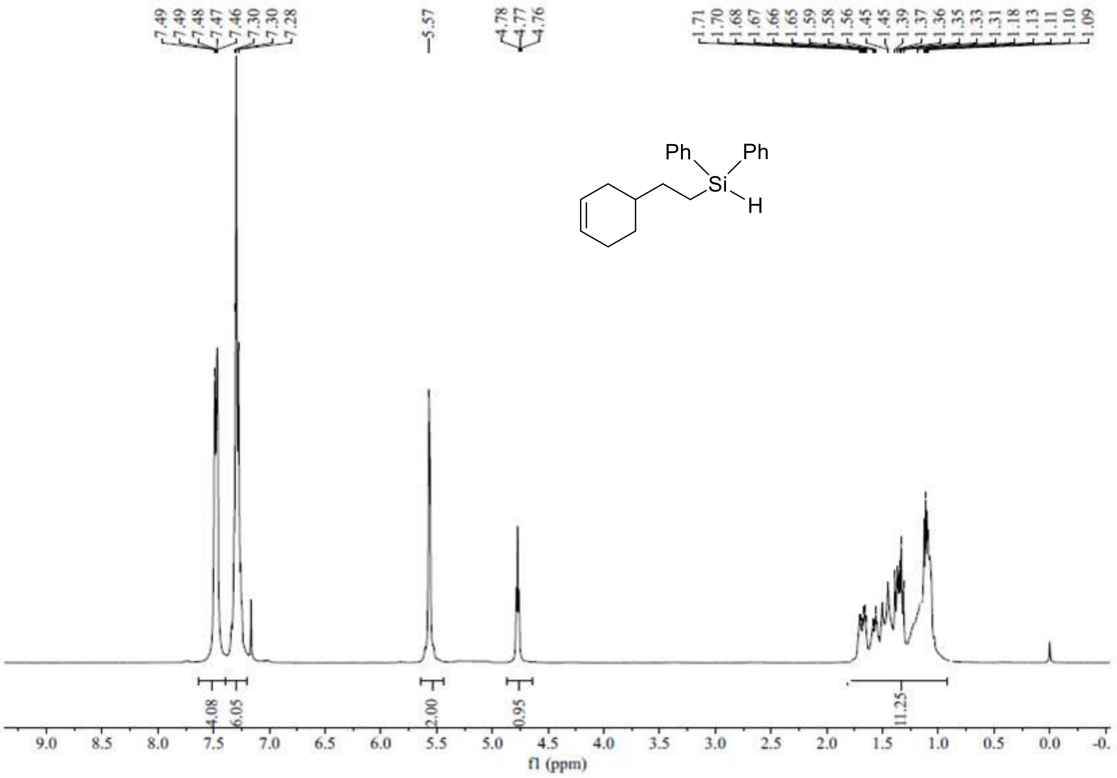

**Figure S31.** $^{1}$H-NMR spectrum of (2-(cyclohex-3-en-1-yl)ethyl)diphenylsilane.

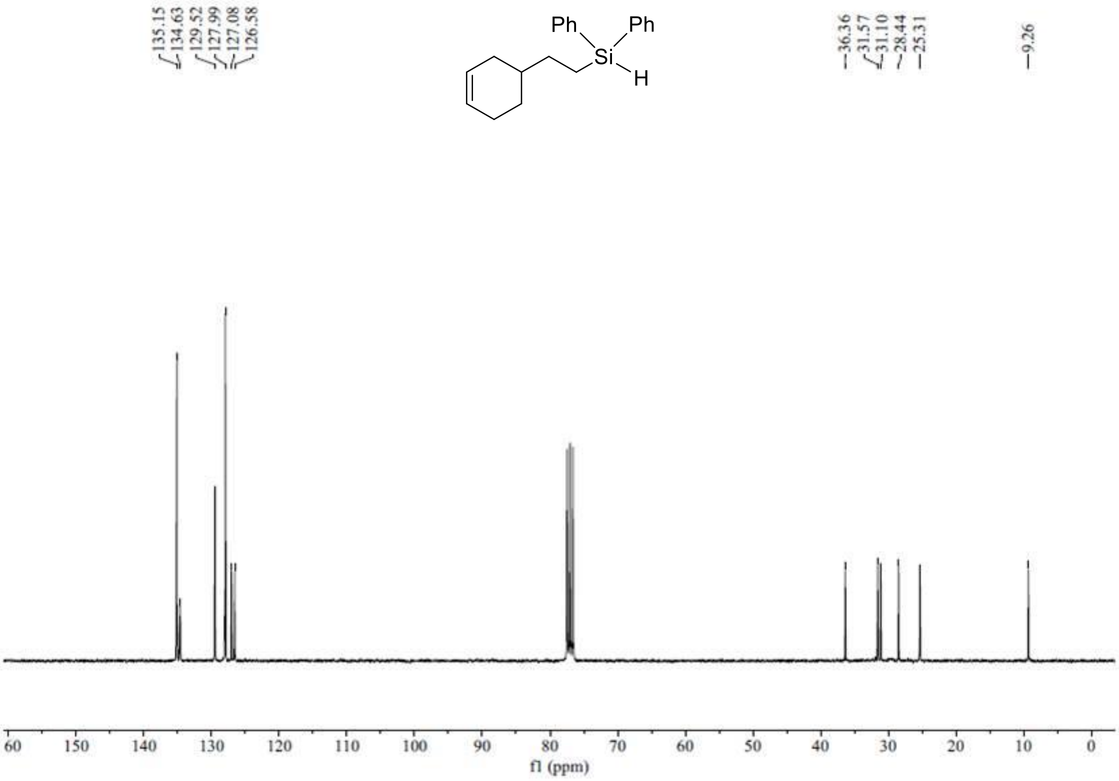

**Figure S32.** $^{13}$C-NMR spectrum of (2-(cyclohex-3-en-1-yl)ethyl)diphenylsilane.

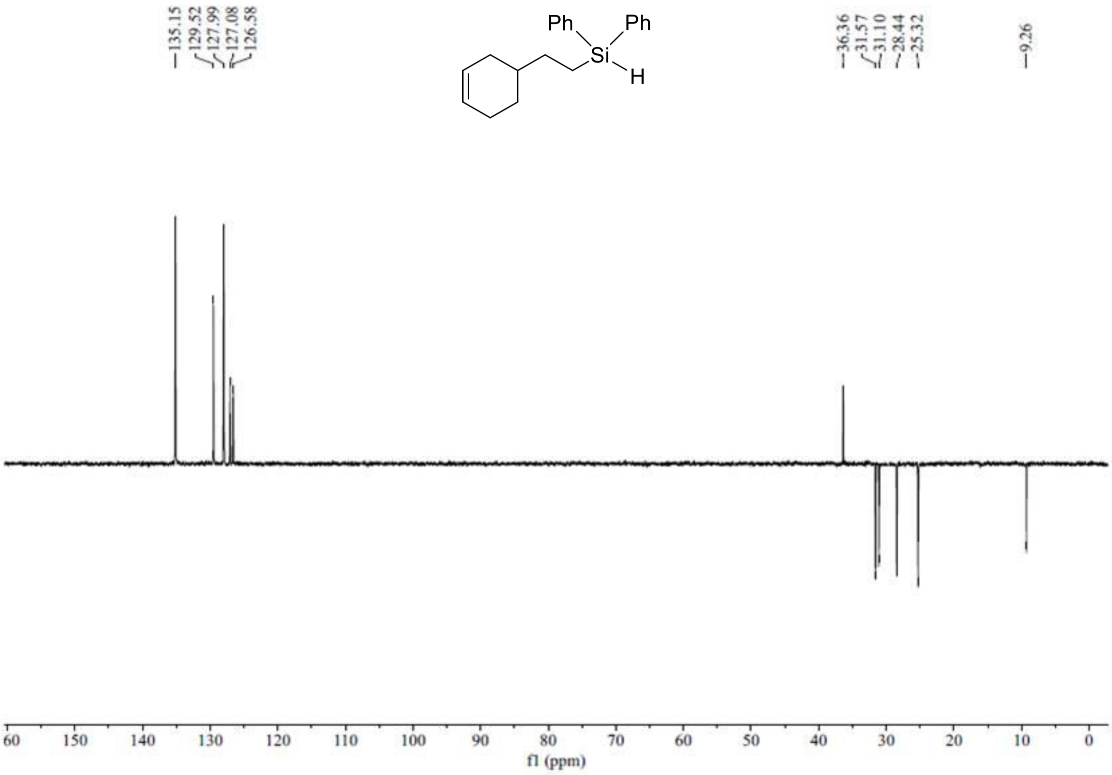


**Figure S33.** DEPT-NMR spectrum of (2-(cyclohex-3-en-1-yl)ethyl)diphenylsilane.

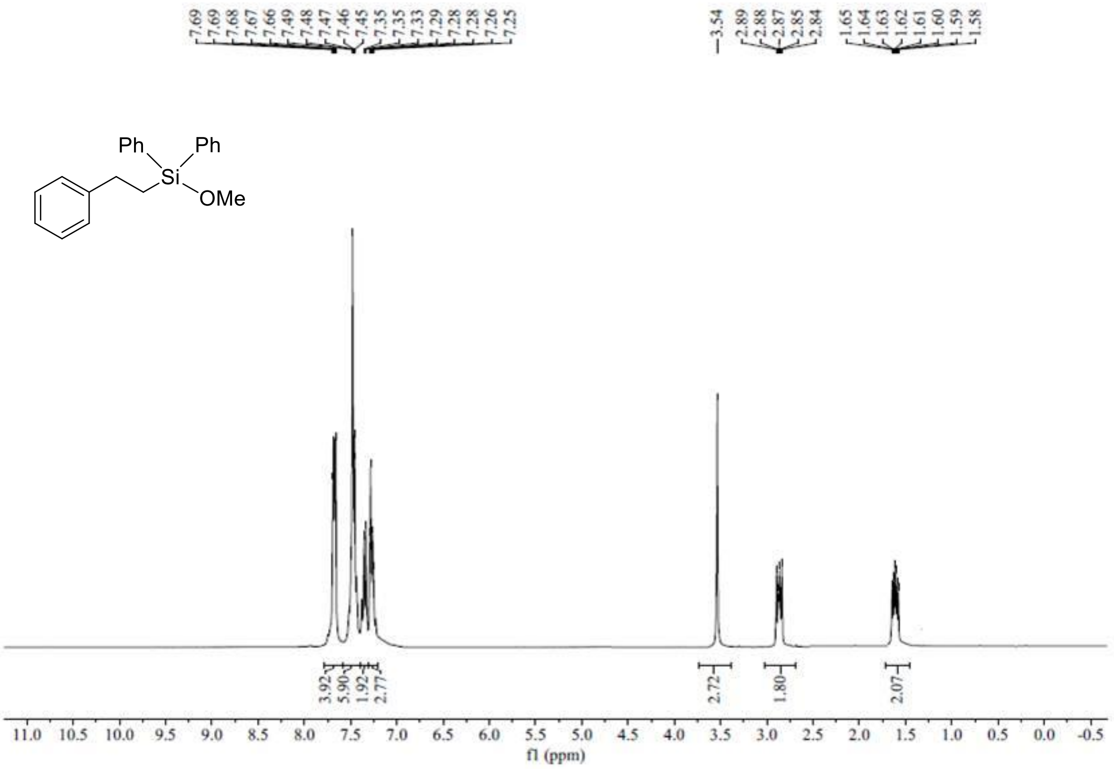


**Figure S34.** $^{1}$H-NMR spectrum of methoxy(phenethyl)diphenylsilane.

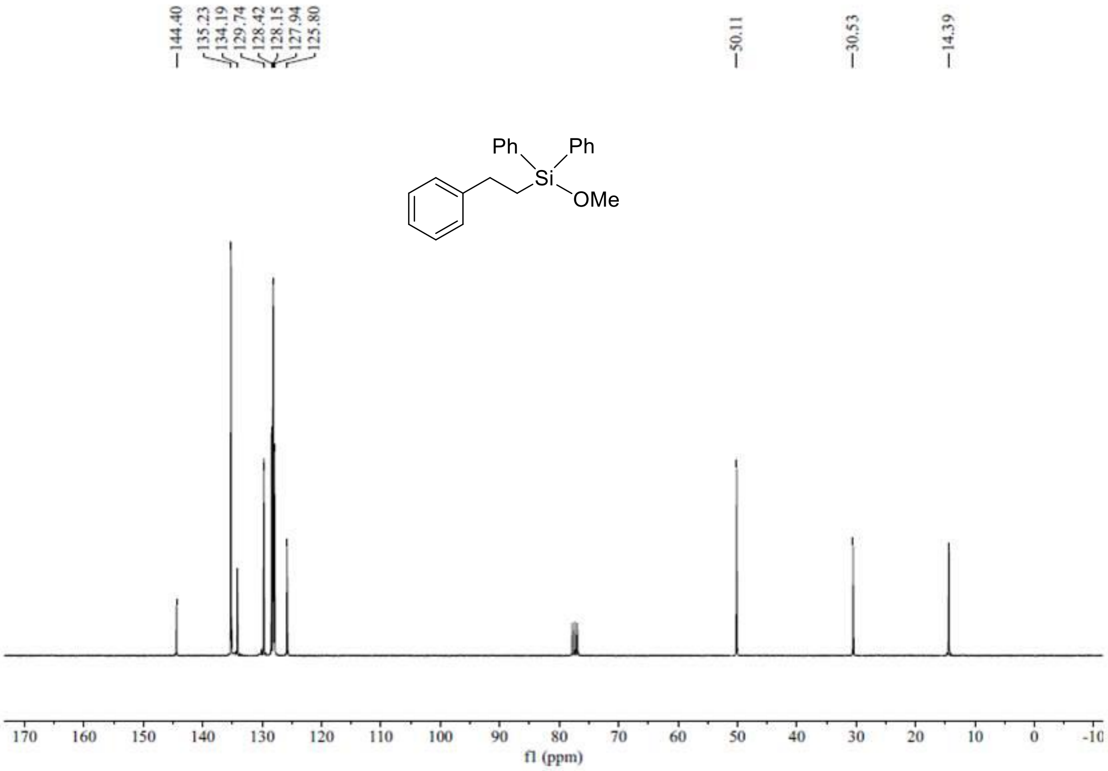


**Figure S35.** $^{13}$C-NMR spectrum of methoxy(phenethyl)diphenylsilane.

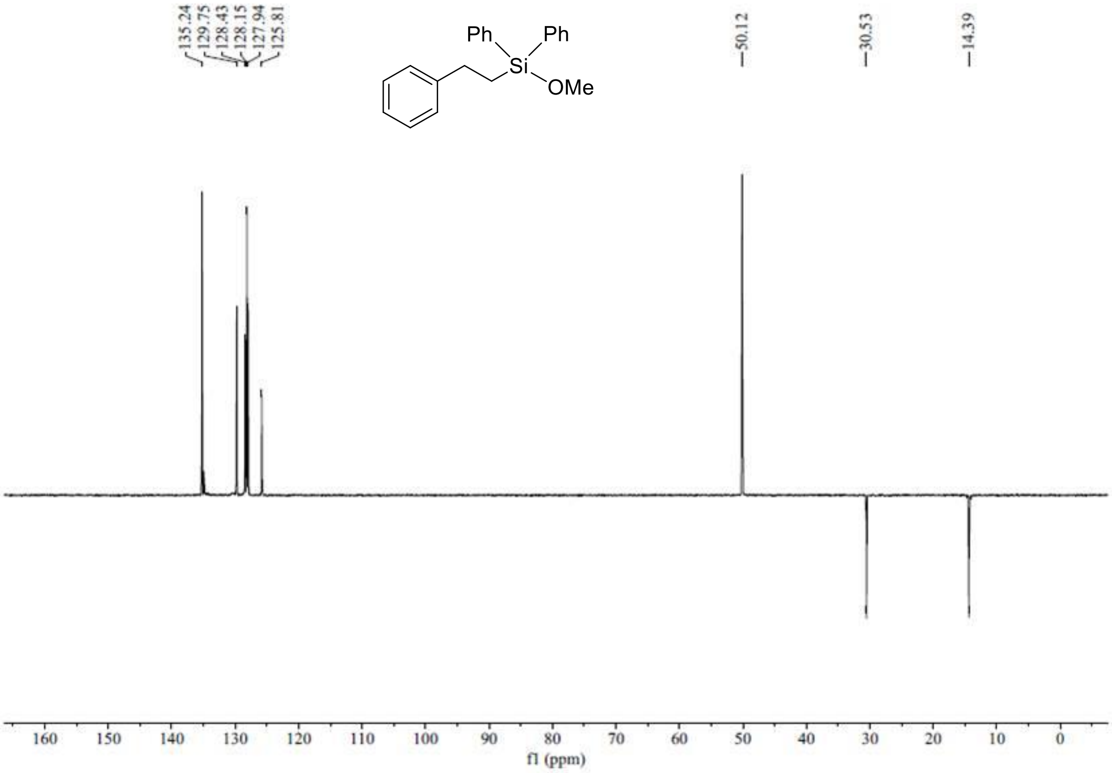


**Figure S36.** DEPT-NMR spectrum of methoxy(phenethyl)diphenylsilane.

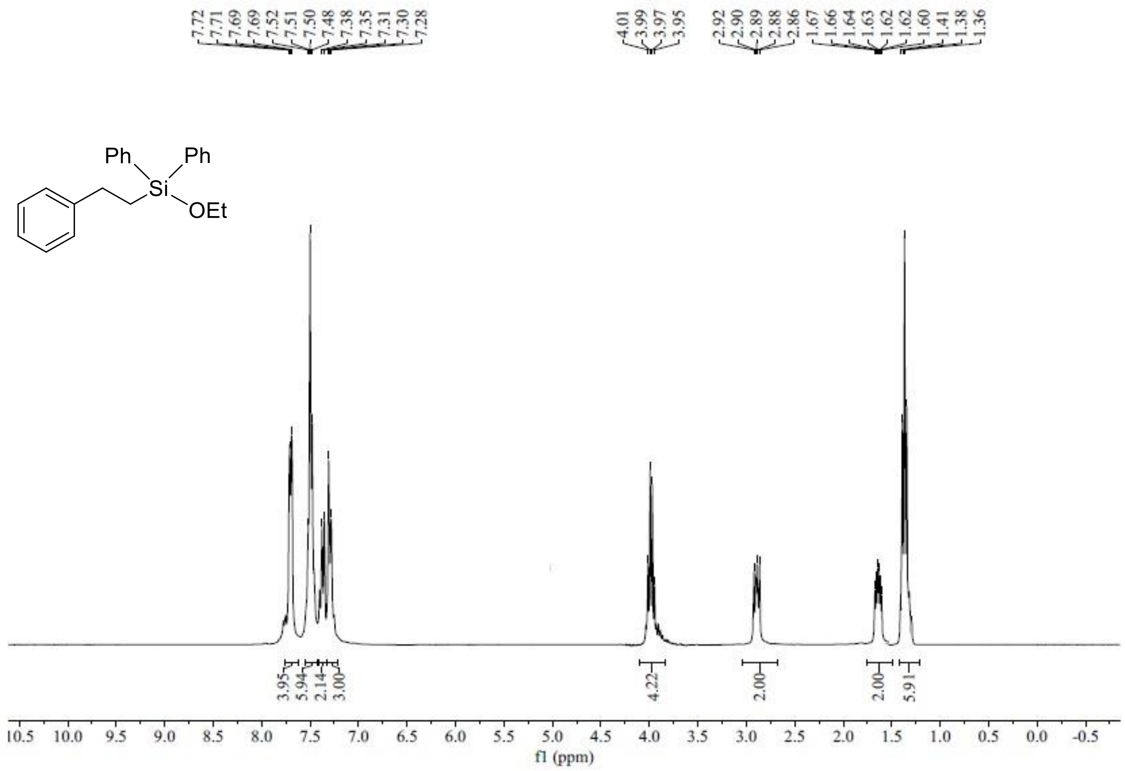


**Figure S37.** $^{1}$H-NMR spectrum of ethoxy(phenethyl)diphenylsilane.

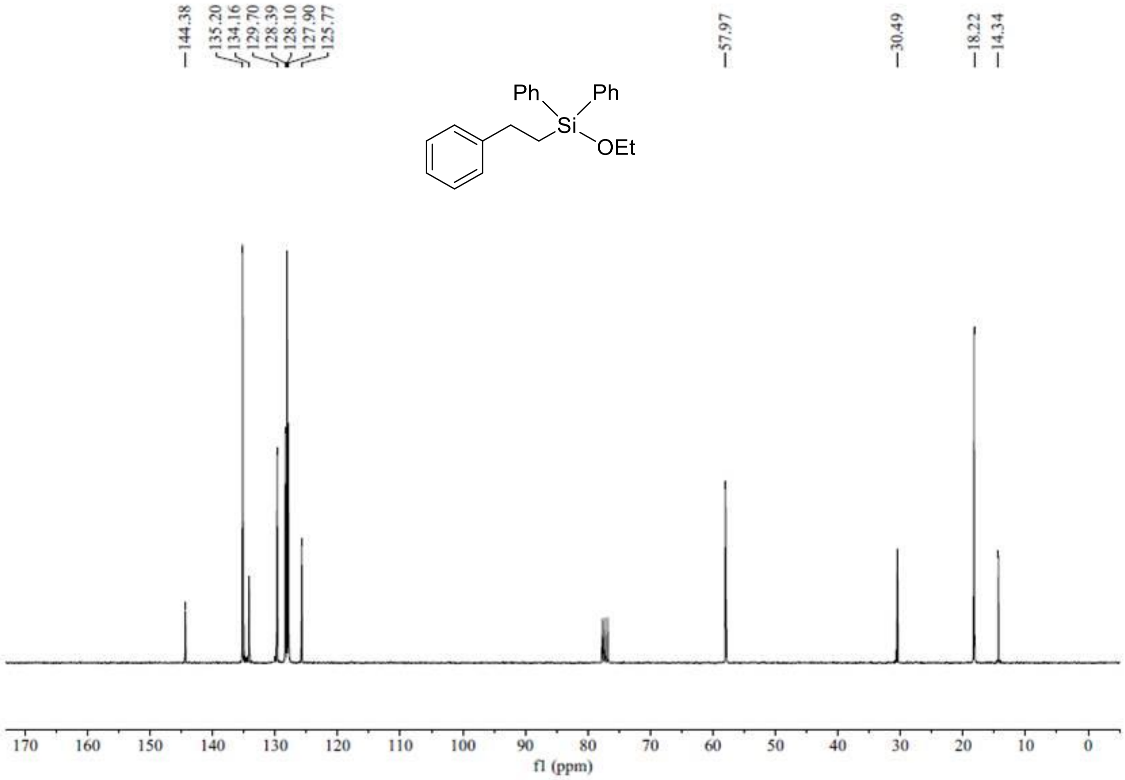


**Figure S38.** $^{13}$C-NMR spectrum of ethoxy(phenethyl)diphenylsilane.

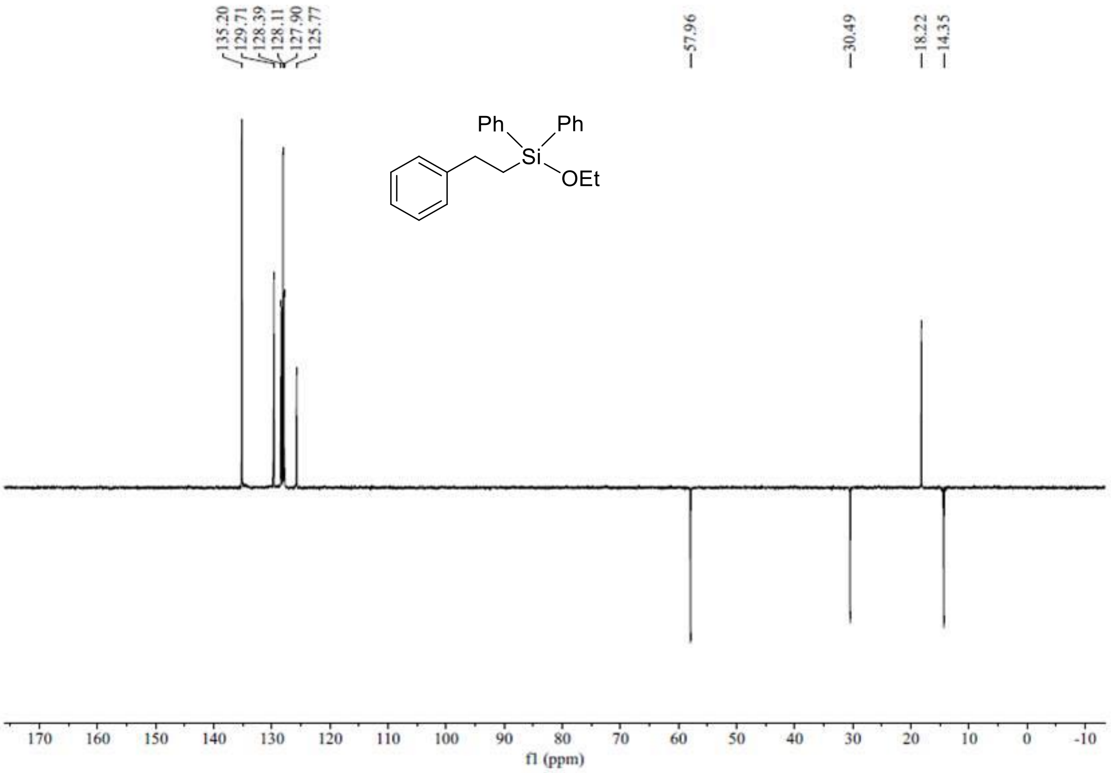


**Figure S39.** DEPT-NMR spectrum of ethoxy(phenethyl)diphenylsilane.

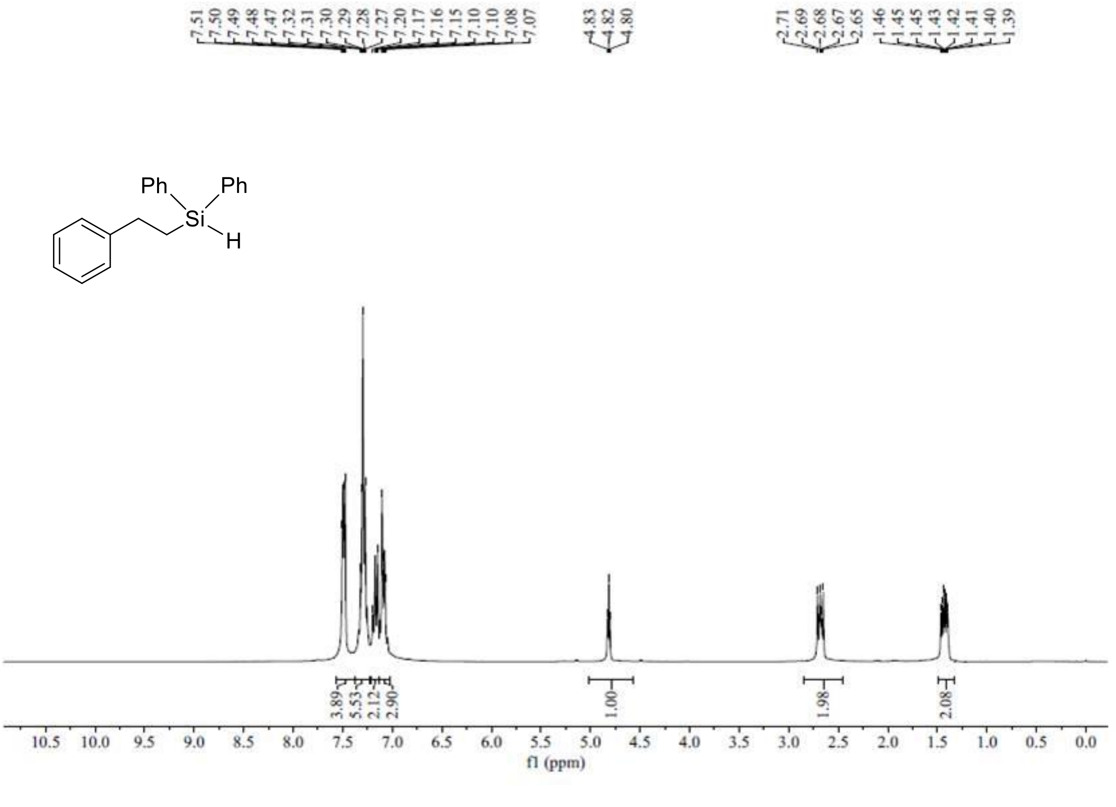


**Figure S40.** $^{1}$H-NMR spectrum of (phenethyl)diphenylsilane.

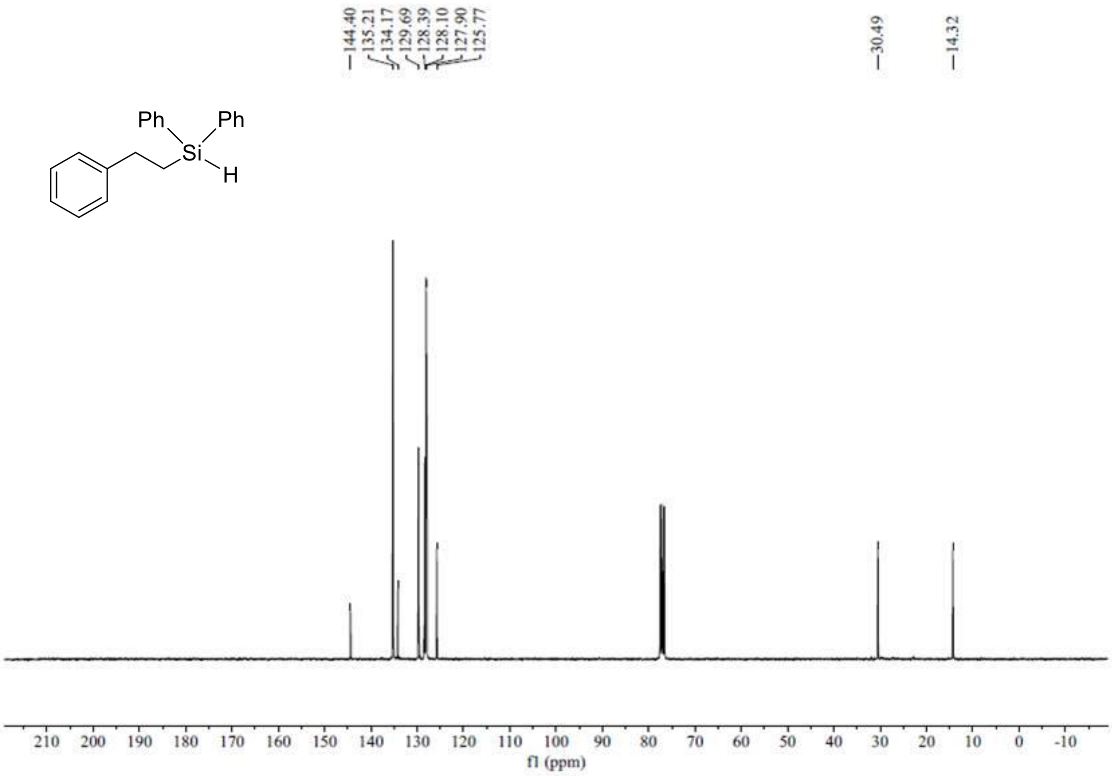


**Figure S41.** $^{13}$C-NMR spectrum of (phenethyl)diphenylsilane.

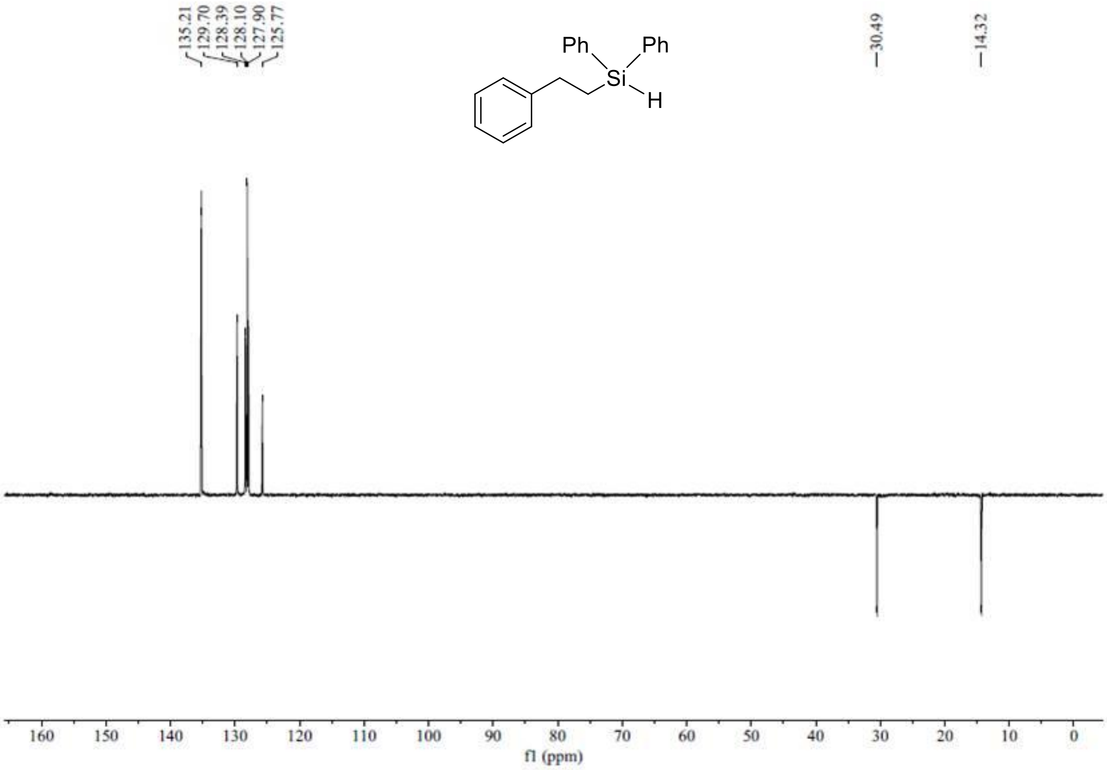


**Figure S42.** DEPT-NMR spectrum of (phenethyl)diphenylsilane.

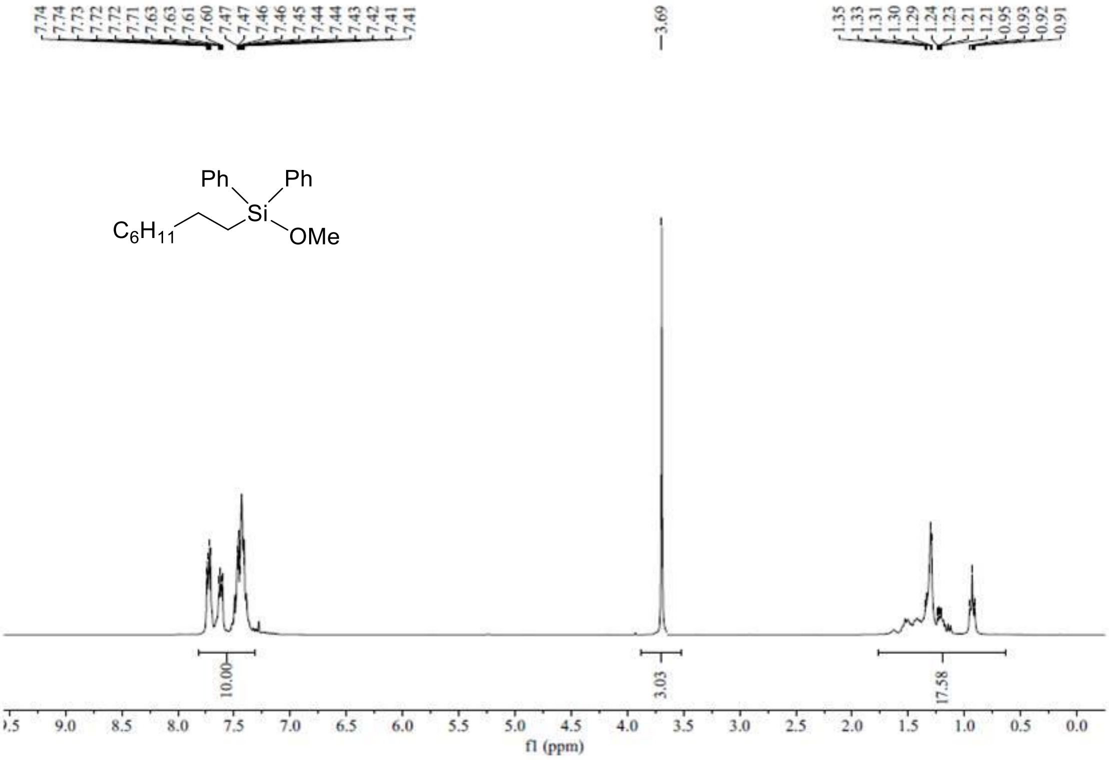


**Figure S43.** $^{1}$H-NMR spectrum of Methoxy(octyl)diphenylsilane.

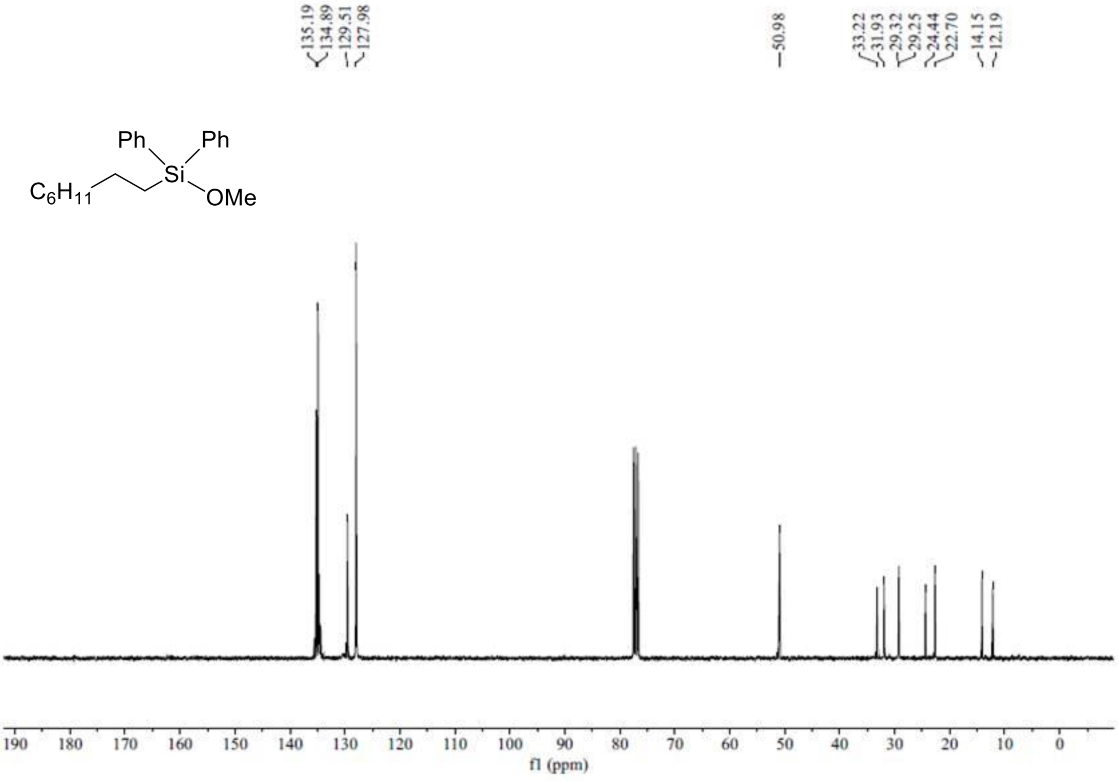


**Figure S44.** $^{13}$C-NMR spectrum of Methoxy(octyl)diphenylsilane.

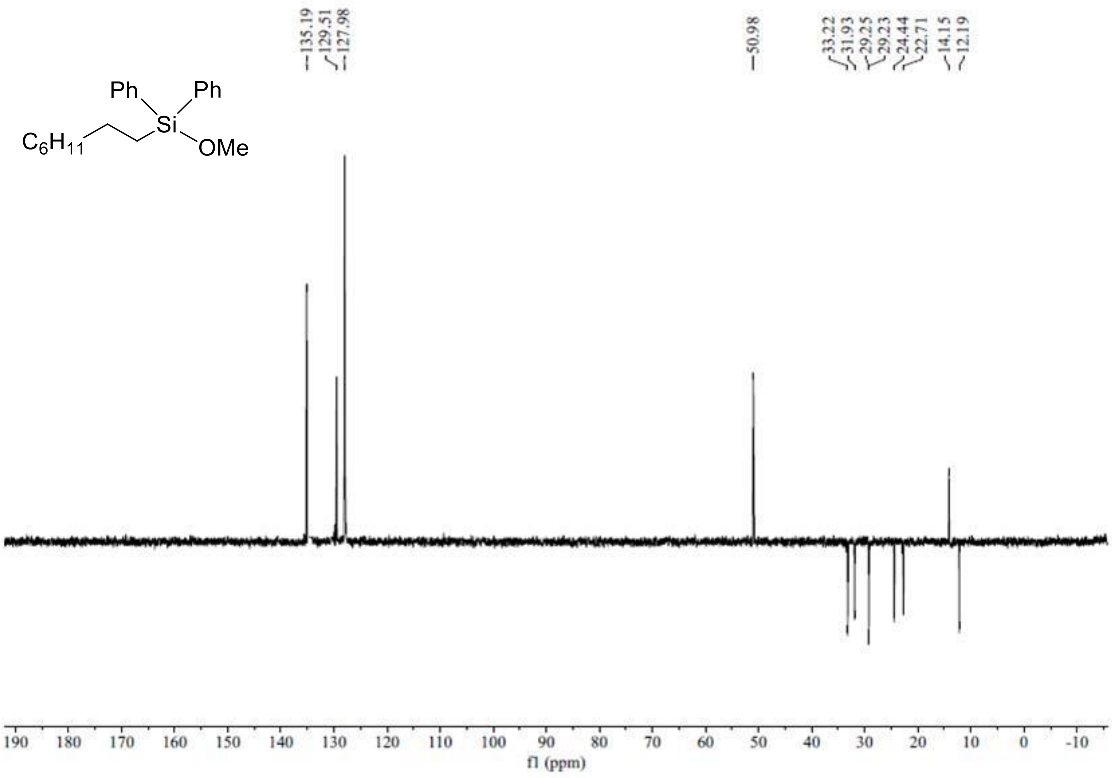


**Figure S45.** DEPT-NMR spectrum of methoxy(octyl)diphenylsilane.

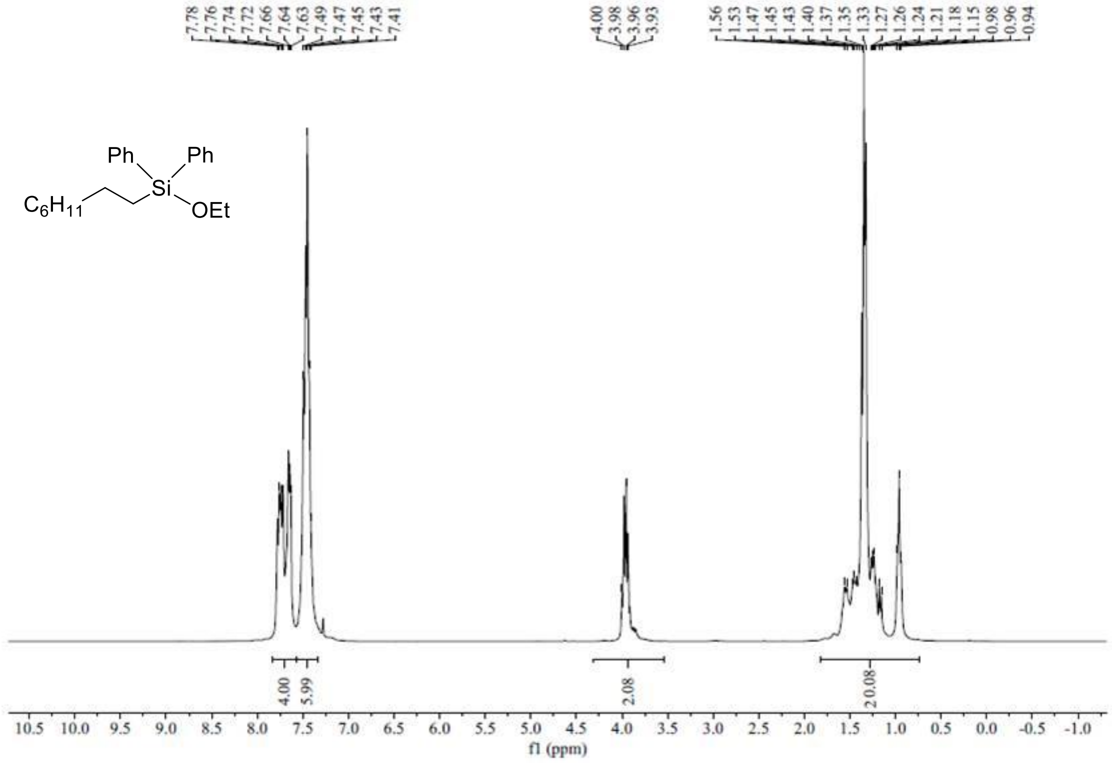


**Figure S46.** $^1$H-NMR spectrum of ethoxy(octyl)diphenylsilane.

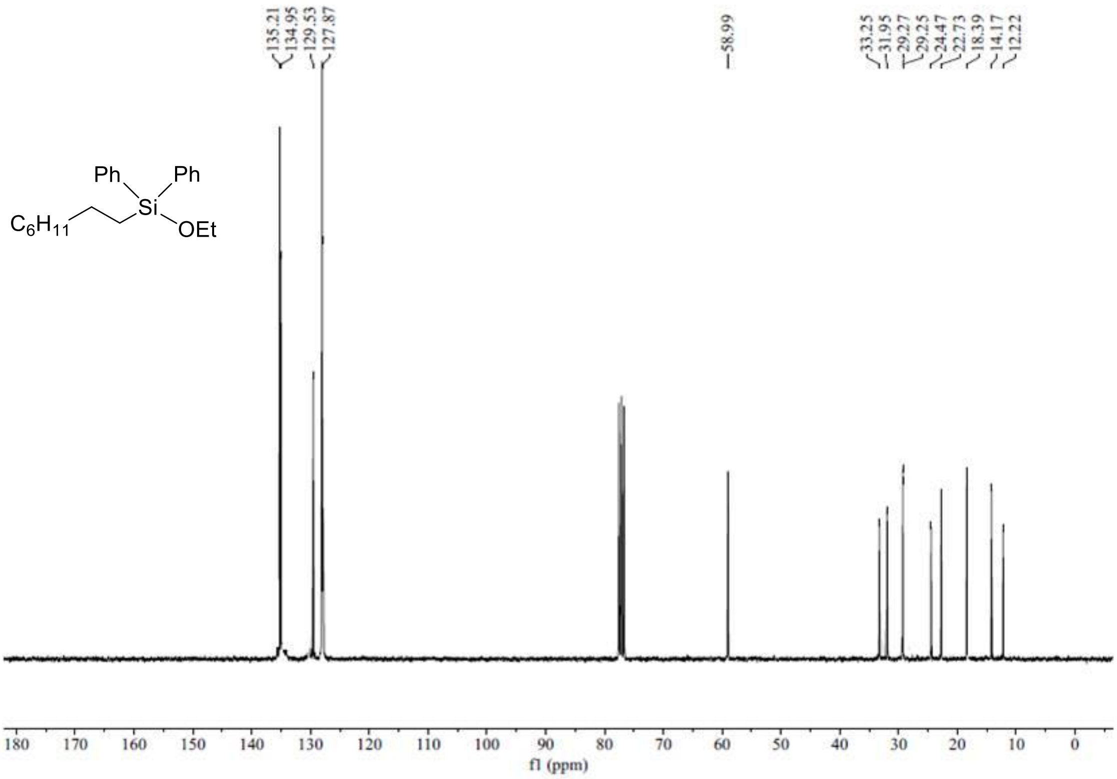


**Figure S47.** $^{13}$C-NMR spectrum of ethoxy(octyl)diphenylsilane

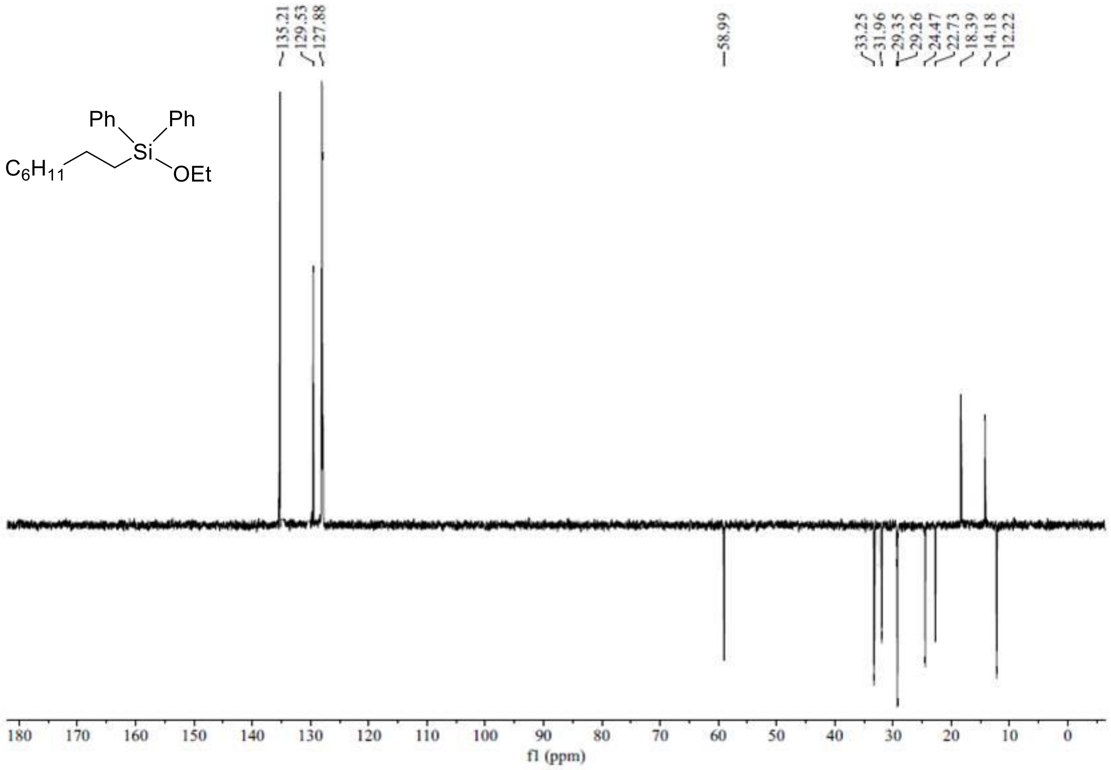


**Figure S48.** DEPT-NMR spectrum of ethoxy(octyl)diphenylsilane

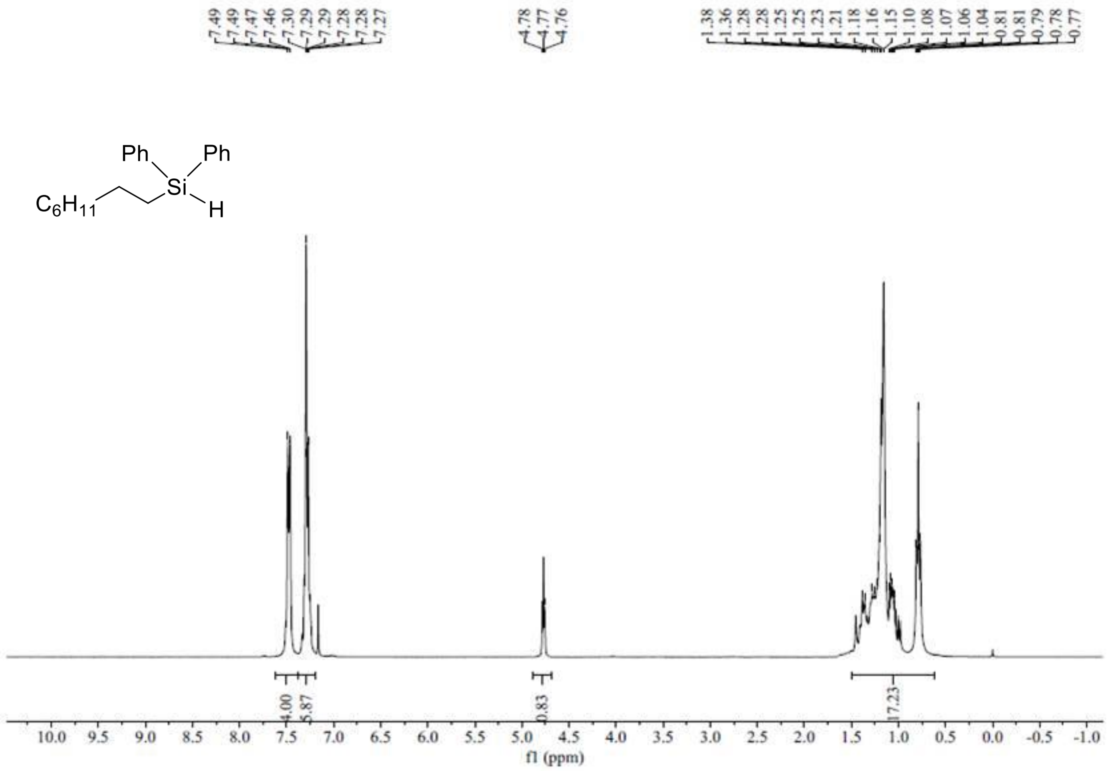

**Figure S49.** $^{1}$H-NMR spectrum of octyl(diphenyl)silane.

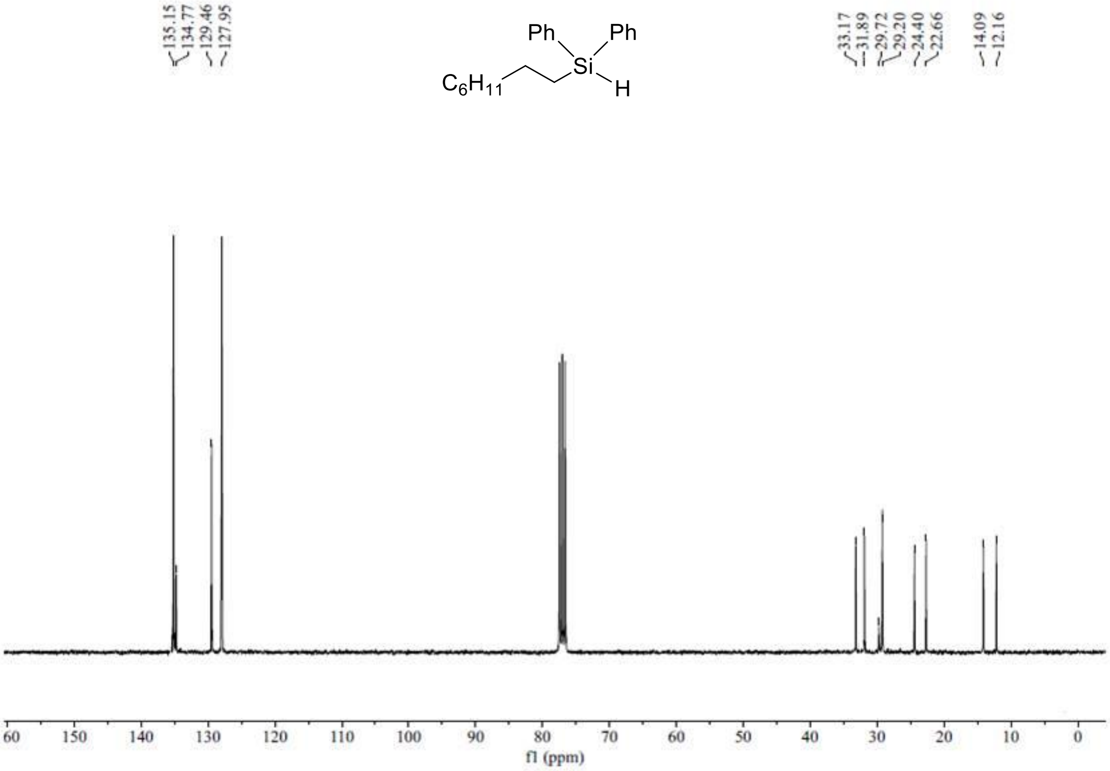

**Figure S50.** $^{13}$C-NMR spectrum of octyl(diphenyl)silane.

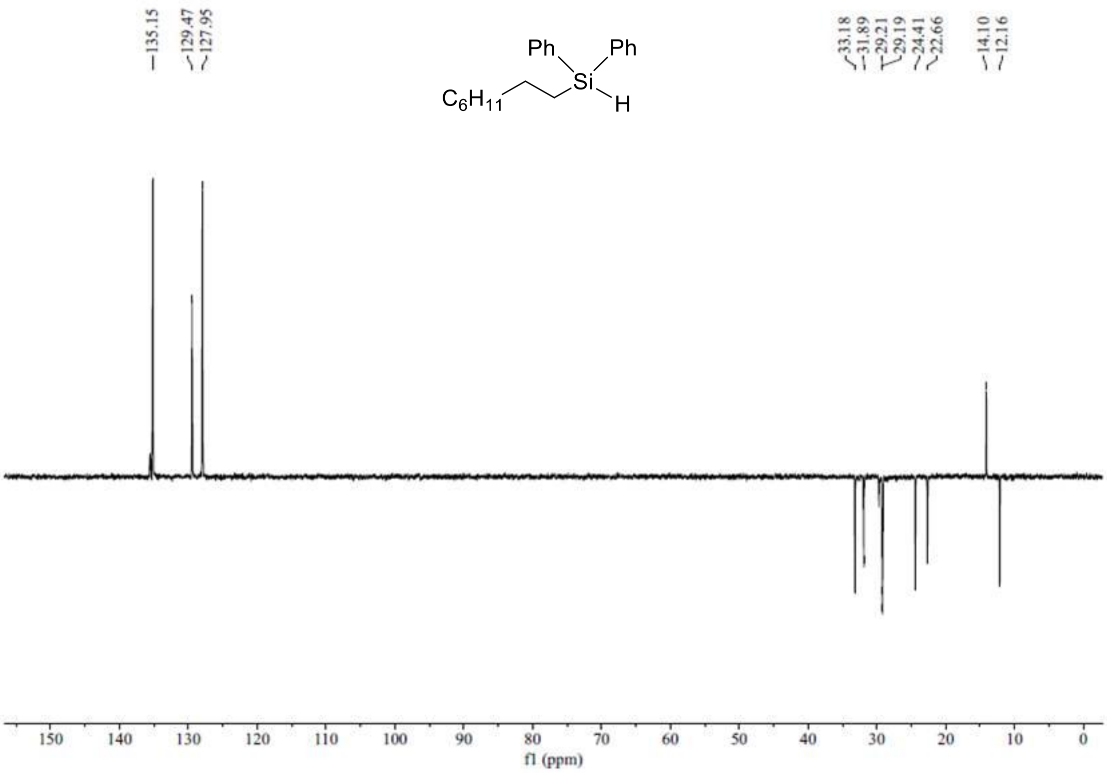


**Figure S51.** DEPT-NMR spectrum of octyl(diphenyl)silane.

## V. References


(1) Gutiérrez-Tarriño, S.; Concepción, P.; Oña-Burgos, P. Cobalt Catalysts for Alkene Hydrosilylation under Aerobic Conditions without Dry Solvents or Additives. *Eur. J. Inorg. Chem.* **2018**, *2018* (45), 4867–4874. https://doi.org/10.1002/ejic.201801068.

(2) Becke, A. D. The Limiting High Temperature Rotational Partition Function of Nonrigid Molecules: I. General Theory. II. CH4, C2H6, C3H8, CH(CH3)3, C(CH 3)4 and CH3(CH2)2CH 3. III. Benzene and Its Eleven Methyl Derivatives. *Phys. Rev. A* **1988**, *38* (6), 3098–3100. https://doi.org/10.1063/1.1749835.

(3) C. Lee, W. Yang, R. G. P. Maintainng a Heathy Rhythm. *Phys. Rev. B* **1988**, *37* (2), 785–789.

(4) Stephen, P. J.; Devlin, F. J.; Chabalowski, C. F.; Frisch, M. J. Ab Initio Calculation of Vibrational Absorption. *J. Phys. Chem.* **1994**, *98* (45), 11623–11627.

(5) Grimme, S.; Antony, J.; Ehrlich, S.; Krieg, H. A Consistent and Accurate Ab Initio Parametrization of Density Functional Dispersion Correction (DFT-D) for the 94 Elements H-Pu. *J. Chem. Phys.* **2010**, *132* (15). https://doi.org/10.1063/1.3382344.

(6) Tomasi, J.; Mennucci, B.; Cammi, R. Quantum Mechanical Continuum Solvation Models. *Chem. Rev.* **2005**, *105* (8), 2999–3093. https://doi.org/10.1021/cr9904009.

(7) Andrae, D.; Häußermann, U.; Dolg, M.; Stoll, H.; Preuß, H. Energy-Adjusted Ab Initio Pseudopotentials for the Second and Third Row Transition Elements. *Theor. Chim. Acta* **1990**, *77* (2), 123–141. https://doi.org/10.1007/BF01114537.

(8) Ehlers, A. W.; Böhme, M.; Dapprich, S.; Gobbi, A.; Höllwarth, A.; Jonas, V.; Köhler, K. F.; Stegmann, R.; Veldkamp, A.; Frenking, G. A Set of F-Polarization Functions for Pseudo-Potential Basis Sets of the Transition Metals ScCu, YAg and LaAu. *Chem. Phys. Lett.* **1993**, *208* (1–2), 111–114. https://doi.org/10.1016/0009-2614(93)80086-5.

(9) Francl, M. M.; Pietro, W. J.; Hehre, W. J.; Binkley, J. S.; Gordon, M. S.; DeFrees, D. J.; Pople, J. A. Self-Consistent Molecular Orbital Methods. XXIII. A Polarization-Type Basis Set for Second-Row Elements. *J. Chem. Phys.* **1982**, *77* (7), 3654–3665. https://doi.org/10.1063/1.444267.

(10) Hariharan, P. C.; Pople, J. A. The Influence of Polarization Functions on Molecular Orbital Hydrogenation Energies. *Theor. Chim. Acta* **1973**, *28* (3), 213–222. https://doi.org/10.1007/BF00533485.

(11) Grimme, S. Supramolecular Binding Thermodynamics by Dispersion-Corrected Density Functional Theory. *Chem. - A Eur. J.* **2012**, *18* (32), 9955–9964. https://doi.org/10.1002/chem.201200497.

(12) Luchini, G.; Alegre-Requena, J. V; Funes-Ardoiz, I.; Paton, R. S. GoodVibes: Automated Thermochemistry for Heterogeneous Computational Chemistry Data [Version 1; Peer

Review: 2 Approved with Reservations]. *F1000Research* **2020**, *9* (291), 1–14.

(13) Krishnan, R.; Binkley, J. S.; Seeger, R.; Pople, J. A. Self-Consistent Molecular Orbital Methods. XX. A Basis Set for Correlated Wave Functions. *J. Chem. Phys.* **1980**, *72* (1), 650–654. https://doi.org/10.1063/1.438955.

(14) McLean, A. D.; Chandler, G. S. Contracted Gaussian Basis Sets for Molecular Calculations. I. Second Row Atoms, Z=11-18. *J. Chem. Phys.* **1980**, *72* (10), 5639–5648. https://doi.org/10.1063/1.438980.

(15) Clark, T.; Chandrasekhar, J.; Spitznagel, G. W.; Schleyer, P. V. R. Efficient Diffuse Function-augmented Basis Sets for Anion Calculations. III. The 3-21+G Basis Set for First-row Elements, Li–F. *J. Comput. Chem.* **1983**, *4* (3), 294–301. https://doi.org/10.1002/jcc.540040303.

(16) Álvarez-Moreno, M.; De Graaf, C.; López, N.; Maseras, F.; Poblet, J. M.; Bo, C. Managing the Computational Chemistry Big Data Problem: The IoChem-BD Platform. *J. Chem. Inf. Model.* **2015**, *55* (1), 95–103. https://doi.org/10.1021/ci500593j.

(17) Manuel A. Ortuño, database: https://www.iochem-bd.org/handle/10/290044

(18) Yuan, W.; Orecchia, P.; Oestreich, M. Palladium-Catalyzed Three-Component Reaction of Dihydrosilanes and Vinyl Iodides in the Presence of Alcohols: Rapid Assembly of Silyl Ethers of Tertiary Silanes. *Chem. - A Eur. J.* **2018**, *24* (72), 19175–19178. https://doi.org/10.1002/chem.201805595.
